# The prognostic value of MEK pathway–associated estrogen receptor signaling activity for female cancers

**DOI:** 10.1038/s41416-024-02668-w

**Published:** 2024-04-06

**Authors:** Chun Wai Ng, Yvonne T. M. Tsang, David M. Gershenson, Kwong-Kwok Wong

**Affiliations:** Department of Gynecologic Oncology and Reproductive Medicine, The University of Texas MD Anderson Cancer Center, Houston, TX USA

**Keywords:** Gynaecological cancer, Prognostic markers

## Abstract

**Background:**

Other than for breast cancer, endocrine therapy has not been highly effective for gynecologic cancers. Endocrine therapy resistance in estrogen receptor positive gynecologic cancers is still poorly understood. In this retrospective study, we examined the estrogen receptor (ER) signaling pathway activities of breast, ovarian, endometrial, and cervical cancers to identify those that may predict endocrine therapy responsiveness.

**Methods:**

Clinical and genomic data of women with breast and gynecological cancers were downloaded from cBioPortal for Cancer Genomics. Estrogen receptor alpha (ESR1) expression level and sample-level pathway enrichment scores (EERES) were calculated to classify patients into four groups (low/high ESR1 and low/high EERES). Correlation between ESR1/EERES score and survival was further validated with RNAseq data from low-grade serous ovarian cancer. Pathway analyses were performed among different ESR1/EERES groups to identify genes that correlate with endocrine resistance, which are validated using Cancer Cell Line Encyclopedia gene expression and Genomics of Drug Sensitivity in Cancer data.

**Results:**

We identified a novel combined prognostic value of *ESR1* expression and the corresponding estrogen response signaling (EERES score) for breast cancer. The combined prognostic value (ESR1/EERES) may be applicable to other gynecologic cancers. More importantly, we discovered that ER signaling can cross-regulate MEK pathway activation. We identified downstream genes in the MEK pathway (*EPHA2*, *INAVA*, *MALL*, *MPZL2*, *PCDH1*, and *TNFRSF21*) that are potential endocrine therapy response biomarkers.

**Conclusion:**

This study demonstrated that targeting both the ER and the ER signaling activity related MEK pathway may aid the development of endocrine therapy strategies for personalized medicine.

## Introduction

Estrogen and estrogen receptor (ER) signaling pathways are involved in the development of female cancers [1–6]. Deregulated ER expression and ER signaling can lead to uncontrolled cell proliferation and cancer progression. During tumorigenesis, normal ER signaling functions to support benign cell differentiation and development are reprogrammed to support tumor cell proliferation [7, 8]. Endocrine therapy targeting this signaling pathway by blocking the activity of ERα has long been an option for the treatment of female cancers [9, 10]. The three main categories of hormonal therapeutics are selective ER modulators, aromatase inhibitors, and selective ER degraders. These ERα-targeting pharmaceutical agents are effective in treating ERα-positive breast cancer. Specifically, they can slow the growth of or shrink breast tumors and reduce the risk of recurrence after surgery [11]. Although gynecologic cancers have long been recognized to be caused by abnormal estrogen pathway activity, the effectiveness of endocrine therapy for gynecologic cancers other than breast cancer is still controversial, and biomarkers for precision treatment of them are needed [12].

In a retrospective study, hormonal maintenance therapy resulted in a lower risk of progression in patients with stage II-IV low-grade ovarian serous carcinoma than in patients who underwent observation only but did not result in a significant difference in overall survival (OS) [13]. Letrozole has been suggested to be valuable as a maintenance treatment of high-grade serous ovarian cancer, especially in patients with chemoresistance or residual disease [14]. Another retrospective study indicates that endocrine therapy could be a practical strategy to postpone subsequent chemotherapy for relapsed high-grade serous ovarian cancer [15]. From a phase II study of anastrozole in patients with estrogen receptor-positive recurrent/metastatic low-grade ovarian cancers and serous borderline ovarian tumors, partial responses were only observed in 14% patients (5/36) [16]. From systematic reviews and meta-analyses of over 50 phase II trials of endocrine therapies in epithelial ovarian cancer, a similar response rate between 10 to 15% was determined [12, 17]. Tamoxifen alone or in combination with progestin was suggested to be the preferred choice when selecting second-line hormonal treatment of endometrial cancer after first-line treatment with progestin [18]. Nevertheless, only about 10% of ovarian, endometrial, and cervical cancers respond to hormonal therapy [19–22]. Currently, hormone therapy is not routinely recommended in the adjuvant setting for endometrial cancer but could be an alternative for selected patients with specific molecular profiles [23]. Furthermore, tamoxifen is reported to stimulate the growth of endometrial cancer cell lines [24]. As a standard of practice, selection of patients for hormone therapy is based on ER/progesterone receptor status according to immunostaining. However, high ER expression may not always correlate with high ER signaling activity. Therefore, looking at both ER expression and ER signaling activity is important for improving the selection of women who would benefit most from hormone therapy.

Endocrine therapy resistance in estrogen receptor positive gynecologic cancers is still poorly understood. In this retrospective study, we analyzed data in The Cancer Genome Atlas (TCGA) Pan-Cancer Atlas for ER expression and downstream signaling activity in breast, ovarian, endometrial, and cervical cancer patients to elucidate the differences in ER cell signaling and survival among these four cancers [25, 26]. Such analyses could provide insight into endocrine resistance for therapeutic development. Researchers found ER signaling activity to be a prognostic factor for endocrine therapy for breast cancer [27]. We hypothesized that ER signaling activity would differ in other gynecologic cancers and that alternative signaling pathways are activated to bypass ER signaling in these cancer cells. Therefore, we analyzed ER and other oncogenic signaling pathways to understand the differential molecular activity among these four cancer types to discover potential therapeutic targets to overcome endocrine therapy resistance.

## Results

### ER signaling is prognostic for gynecologic cancers

We downloaded gene expression data from the TCGA Pan-Cancer Atlas data sets for breast invasive carcinoma (BRCA; *n* = 1095), ovarian serous cystadenocarcinoma (OV; *n* = 378), uterine corpus endometrial carcinoma (UCEC; *n* = 557), and cervical squamous cell carcinoma (CESC; *n* = 304) from the cBioPortal for Cancer Genomics (https://cbioportal.org). We determined the early estrogen response enrichment scores (EERESs) for each sample via gene set variation analysis (GSVA) [28] using the HALLMARK_ESTROGEN_RESPONSE_EARLY gene set from the Molecular Signatures Database (MSigDB). This is a set of 200 genes upregulated in breast cancer cells after estrogen stimulation [29–31]. The EERES for each sample of each cancer type is shown in Supplementary Table S1.

**Fig. 1:**
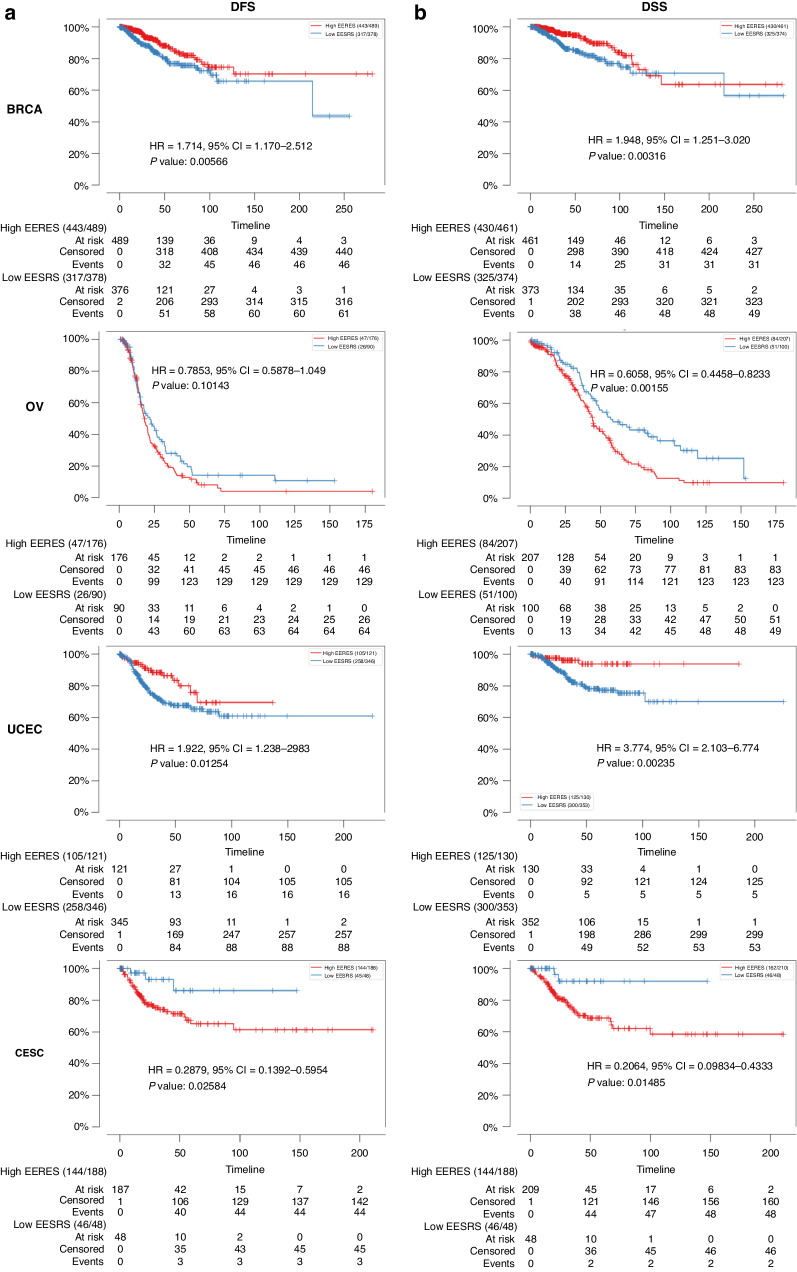
ER signaling is prognostic for gynecologic cancers. Samples of the breast, ovarian, endometrial, and cervical cancers from TCGA were designated as having a low EERES (blue curve, at or below threshold) or high EERES (red curve, greater than threshold) as described in the method section. Kaplan-Meier curves with log-rank HRs (Hazard Ratio), 95% CIs, and *P* values for (**a**) DFS and (**b**) DSS in the two groups over time are shown.

To determine whether ER signaling activity is a prognostic marker or a biomarker for endocrine therapy response, we first classified samples of breast, ovarian, endometrial, and cervical cancers in the TCGA data sets as having low or high ER signaling activity by determining the EERESs for each sample as described in the “Methods” section. The correlations of low or high ER activities with disease-free survival (DFS) and disease-specific survival (DSS) were presented in Fig. 1a, b. The EERES thresholds for classifying BRCA, OV, UCEC, and CESC as low or high ER activities were –0.0512, –0.184, 0.157, and –0.228, respectively. The DFS and DSS times in the BRCA patients (DFS: hazard ratio [HR], 1.714 [95% CI, 1.170–2.512]; DSS: HR, 1.948 [95% CI, 1.251–3.020]) and UCEC patients (DFS: HR, 1.922 [95% CI, 1.238–2.983]; DSS: HR, 3.774 [95% CI, 2.103–6.774]) with high EERESs were markedly longer than those in the low EERES group. On the other hand, the DFS and DSS times in the OV patients (DFS: HR, 0.7853 [95% CI, 0.5878–1.049]; DSS: HR, 0.6058 [95% CI, 0.4458–0.8233]) and CESC patients (DFS: HR, 0.2879 [95% CI, 0.1392–0.5954]; DSS: HR, 0.2064 [95% CI, 0.09834–0.4333]) with higher EERESs were considerably shorter than those in the lower EERES group. Similarly, we examined ER signaling activity in these four female cancer samples using the HALLMARK_ESTROGEN_RESPONSE_LATE gene set via GSVA to generate late estrogen response enrichment scores for samples of the four cancer types to classify these samples either high or low enrichment scores, but we found that the scores were less prognostic then EERESs (data not shown). Therefore, we determined that the HALLMARK_ESTROGEN_RESPONSE_EARLY pathway is a better predictive biomarker than the HALLMARK_ESTROGEN_RESPONSE_LATE pathway regarding survival of these patients with female cancers.

Next, we explored the correlation between ESR gene expression and EERES for the four female cancers (Supplementary Fig S1). The correlation of *ESR1* expression with EERES was moderate to high in breast (*r* = 0.641, *P* = 7.038e-128) and endometrial (*r* = 0.413, *P* = 2.142e-24) cancer patients but low in ovarian (*r* = 0.226, *P* = 8.776e-6) and cervical (*r* = 0.264, *P* = 3.182e-6) cancer patients. This demonstrated that in ovarian and cervical cancer patients, ERα expression may not activate the known early estrogen-responsive genes expressed in ER-positive breast cancer patients.

### Correlation between *ESR1* gene expression and EERES is important for the survival of patients given hormone therapy for breast cancer

**Fig. 2:**
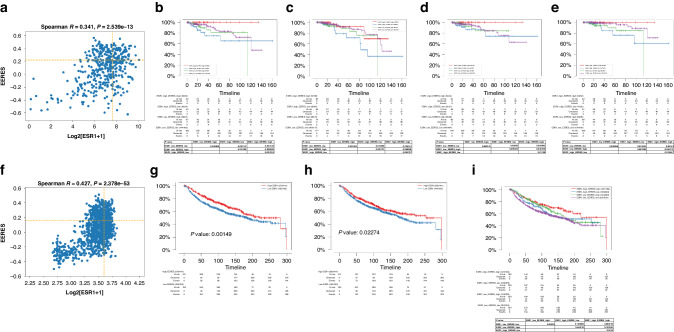
Correlation between *ESR1* gene expression and EERES is important for the survival of patients given hormone therapy for breast cancer. Patients with ER-positive/HER2-negative breast cancer in the TCGA Pan-Cancer Atlas and those given hormonal therapy in the METABRIC were placed in four groups based on low or high *ESR1* expression (quantile, ≤0.65 or >0.65) and low or high EERES (quantile, ≤0.65 or >0.65). Scatter plots and cutoff lines (yellow broken lines) of ESR1 and EERES for the (**a**) TCGA and (**f**) METABRIC samples are shown. Progression-free survival (**b**), OS (**c**), DFS (**d**), and DSS (**e**) times of the TCGA patients for the 4 groups. Relapse-free survival times of the METABRIC patients based on (**g**) *ESR1* expression and (**h**) EERES (quantile, ≤0.65 or >0.65). **i** The relapse-free survival times of the four patient groups in METABRIC.

To show for the first time that the correlation between *ESR1* expression and EERES is important for endocrine therapy responsiveness, we analyzed the survival of ER-positive/HER2-negative breast cancer patients (*n* = 436) using tumor sample data from the TCGA Pan-Cancer Atlas and developed an algorithm to select patients with better survival by selecting patients based on both their *ESR1* expression and EERES. The algorithm was then applied to other gynecologic cancers. This algorithm was further validated by using an independent tumor sample data of breast cancer patients given hormone therapy (*n* = 1174) from the Molecular Taxonomy of Breast Cancer International Consortium (METABRIC). The EERESs for all METABRIC patient tumor samples (*n* = 1904) are shown in Supplementary Table S2. We placed the breast cancer patient tumor samples in four groups according to their *ESR1* expression (up to or higher than quantile 0.65: 199.3 transcripts per million [TPM] for the TCGA Pan-Cancer Atlas patients and 11.3 normalized units for the METABRIC patients) and EERES (up to or higher than quantile 0.65: 0.219 for the TCGA Pan-Cancer Atlas patients and 0.185 for the METABRIC patients). The threshold for the ESR1 and EERES in TCGA dataset were determined with stepwise experiments. By examining the significance of the PFS duration between groups ESR1_low_EERES_low and ESR1_high_EERES_high with increasing threshold (quantile 0.1–0.9, 0.05 per step), the quantile 0.65 was the most significant with *p*-value = 0.031572. The quantile 0.65 was then applied on METABRIC dataset for validation. The correlation of *ESR1* expression and EERES of the hormonal therapy-treated samples from the TCGA Pan-Cancer Atlas and METABRIC patients are shown in Fig. 2a, f, respectively.

We also analyzed the survival of the patients in the four groups described above using Kaplan-Meier curves and log-rank test statistics, the results of which are shown in Fig. 2b–e (progression-free survival, OS, DFS, and DSS, respectively) for the TCGA data and Fig. 2g–i (relapse-free survival) based on *ESR1* expression and EERES for the METABRIC data. The progression-free survival, DFS, and DSS times in the high *ESR1* expression/high EERES group was the longest of the four groups in the TCGA-BRCA data set. In the METABRIC patients, the median relapse-free survival time for the high *ESR1* expression/high EERES patients was the longest (298.88 months), and the low *ESR1* expression/low EERES patients had shorter survival than did the low *ESR1* expression/high EERES and high *ESR1* expression/high EERES groups. These results demonstrated that the correlation of *ESR1* expression with EERES is important for the responsiveness of breast cancer to endocrine therapy.

To evaluate the association between other prognostic factors (i.e., age and stage at diagnosis, and histotype) with the *ESR1* expression/EERES groups, contingency chi-square tests were done (Supplemental Table S3). The distribution of the clinical stages at diagnosis was not significantly different between different ESR1/EERES groups. In contrast, the distribution of age at diagnosis was significantly different between different ESR1/EERES groups (*p* value = 1.47e–6). Notably, there was a higher percentage of patients in the group of the age 61–80 in the groups of ESR1_high_EERES_low (44.3%) and ESR1_high_EERES_high (61.4%) comparing with the groups of ESR1_low_EERES_high (26.5%) and ESR1_low_EERES_low (30.0%). The distribution of histotypes between *ESR1* expression/EERES groups was also significantly different (*p* value = 0.034). Particularly, the percentage of patients with infiltrating lobular carcinoma was lower in groups ESR1_high_EERES_low (13.3%) and ESR1_high_EERES_high (11.4%) when comparing with the groups ESR1_low_EERES_high (24.1%) and ESR1_low_EERES_low (31.0%).

**Fig. 3:**
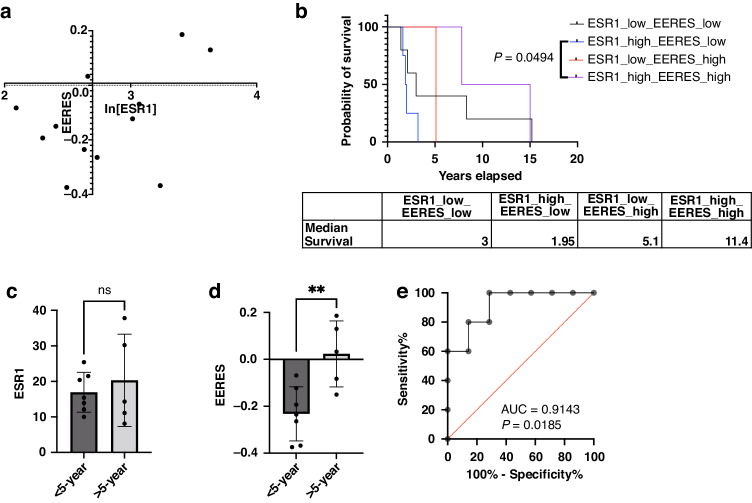
EERES is related to the prognosis for hormone therapy-treated low-grade serous ovarian cancer. The prognostic value of *ESR1* expression and EERES for low-grade serous ovarian cancer was analyzed by (**a**) clustering 12 patients with maintenance hormone therapy into four subgroups according to both *ESR1* expression (quantile, ≤0.5 or >0.5) and EERES (quantile, ≤0.75 or >0.75). **b** OS duration of the four subgroups (with method shown in (**a**)) of the low-grade serous cancer patients analyzed, with median survival and *P* values of log-rank tests shown. Bar charts of *ESR1* expression (**c**) and EERES (**d**) for less than 5-year and greater than 5-year survivors of the 12 low-grade serous cancer patients with unpaired *t* test statistics. **P < 0.01. ns, not significant. **e** The ROC, AUC, and *p* value based on EERES for predicting 5-year survival of the 12 low-grade serous cancer patients are shown. AUC area under the curve. The figures and statistics were generated and determined by GraphPad Prism version 9.4.1.

To further validate the algorithm described above (Fig. 2) to cluster the tumor patient samples based on *ESR1* expression and EERES to select hormonal therapy-treated patients with better responsiveness, we examined 12 patients from MD Anderson with low-grade serous ovarian cancer (LGSOC), who were treated with standard chemotherapy followed by maintenance hormonal therapy [32]. We examined the gene expression profiles using RNA sequencing (RNA-seq) data for these 12 tumors for ER signaling activity analysis by GSVA. Patients were classified according to *ESR1* expression and EERES into four subgroups as described above. The overall survival time, *ESR1* TPMs, and EERESs for the 12 tumor samples are shown in Supplementary Table S4. The results of the correlation of each group and survival are shown in Fig. 3. We found that the high *ESR1* expression/low EERES group had significantly shorter OS than did the high *ESR1* expression/high EERES group (*P* = 0.049) (Fig. 3a, b). However, classification of patients based on the expression of ESR1 alone did not demonstrate any difference in survival (Fig. 3c). On the other hand, classification of patients based on EERES alone were able to predict the long-term survival (>5 years) of patients with low-grade serous ovarian cancer (Fig. 3d) with high specificity and sensitivity (the receiver operating curve [ROC], 0.9143; *P* = 0.0185) (Fig. 3e). However, because of the limited number of patient samples, the predictive value of EERES should be further validated with a large cohort. A phase 3 trial of endocrine therapy for primary low-grade serous cancer of the ovary and peritoneum in a cohort of 450 patients is ongoing (NCT04095364). This trial will provide samples for validating our algorithm in predicting endocrine therapy responsiveness of LGSOC soon.

### ER signaling as a consequence of the development of female cancers

**Fig. 4:**
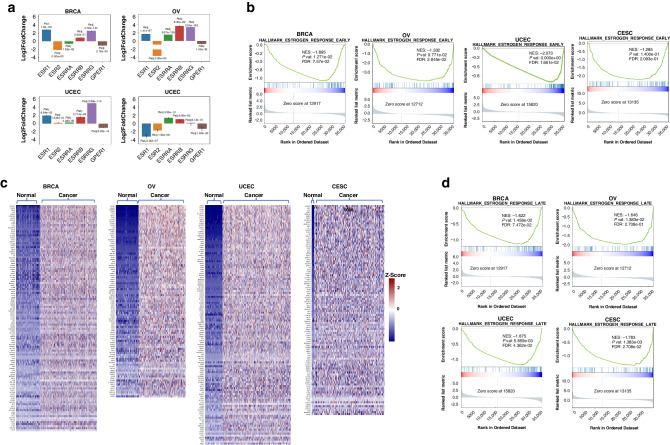
ER signaling as a consequence of the development of female cancers. The role of ER in the development of gynecologic cancers was determined according to the expression pattern for ER-related receptors in normal tissue and tumors. (**a**) Logarithm2 fold change (presented as log2FoldChange) (±SE) in the expression between tumor and normal tissues, and Benjamini-Hochberg adjusted *P* values (padj) for the ER-related receptors *ESR1*, *ESR2*, *ESRRA*, *ESRRB*, *ESRRG*, and *GPER1* as determined using DESeq2. The GSEA pathway enrichment analyses for the 50 MSigDB hallmark pathways were done. The HALLMARK_ESTROGEN_RESPONSE_EARLY pathway enrichment results (**b**) and the differentially regulated genes for the pathway enrichment between normal and tumor tissues in the four female cancers (**c**) are shown. (**d**) ER signaling enrichment in normal tissue and tumors were further revealed by the HALLMARK_ESTROGEN_RESPONSE_LATE pathway enrichment results.

Although we have shown that the correlation between *ESR1* expression and EERES is important for endocrine therapy responsiveness of breast cancer, given their correlation shown in Fig. 1, the reason for the unresponsiveness of other gynecologic cancers to endocrine therapy is unclear. We sought to identify the reason for this by trying to understand the role of ER signaling activity in the development of gynecologic cancers via examination of the difference of the ER signaling pathway activation between normal and tumor tissues. First, we compared the fold change in expression of the ER-related receptors *ESR1*, *ESR2*, *ESRRA*, *ESRRB*, *ESRRG*, and *GPER1* in the four female cancers (Fig. 4a). *ESR1* was upregulated in breast, ovarian, and endometrial tumors but downregulated in cervical tumors. *ESR2* was downregulated in breast, ovarian, and cervical tumors only. In addition, *ESRRA* was downregulated in breast tumors but upregulated in the other tumors, and *ESRRB* and *ESRRG* were upregulated in breast, ovarian, and endometrial tumors. *GPER1* was downregulated in all four female cancers. The role of these receptors in activation of ER signaling should be investigated further.

To examine the dependence of gynecologic oncogenesis on the ER signaling pathway, we performed gene set enrichment analysis (GSEA) of the normal tissue and tumors. The results for the HALLMARK_ESTROGEN_RESPONSE_EARLY pathway are shown in Fig. 4b, and the lead genes in HALLMARK_ESTROGEN_RESPONSE_EARLY pathway for each cancer type are shown in Fig. 4c. Breast and endometrial cancers were enriched in the HALLMARK_ESTROGEN_RESPONSE_EARLY pathway, but ovarian and cervical cancers were not. However, genes related to the HALLMARK_ESTROGEN_RESPONSE_LATE pathway were enriched in all cancer types (Fig. 4d). This suggests that the HALLMARK_ESTROGEN_RESPONSE_EARLY gene expression patterns in ovarian and cervical tumors were like those in their respective normal tissues. Also, enrichment of the HALLMARK_ESTROGEN_RESPONSE_LATE pathway in all four female cancers suggests that the HALLMARK_ESTROGEN_RESPONSE_EARLY pathway was activated in them, as well, as the former pathway follows the latter one. However, the gene expression for the HALLMARK_ESTROGEN_RESPONSE_EARLY pathway may have been interfered with by other pathways.

### MEK pathway activity is associated with ER signaling in patients with the gynecologic cancers

**Fig. 5:**
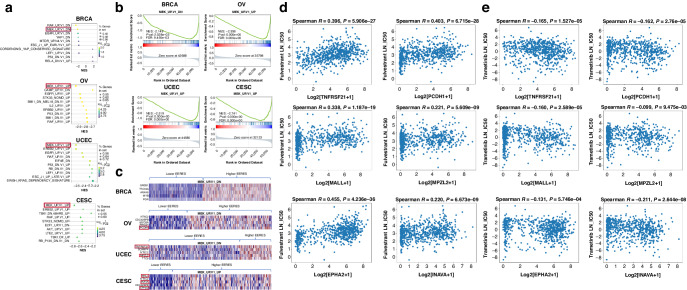
MEK pathway activity is associated with ER signaling in patients with gynecologic cancers. The GSEA pathway enrichment analyses were done using the 189 MSigDB oncogenic pathway gene sets with tumor samples with low and high EERESs identified as described above (Fig. 1a, b). **a** The 10 most significantly enriched oncogenic pathways in gynecologic cancers. The MEK pathways (highlighted) among these top 10 most significantly enriched pathways for each cancer type were selected for further analysis with their enrichment results (MEK_UP.V1_DN for BRCA and MEK_UP.V1.UP for OV, UCEC, and CESC). The pathway enrichment results (**b**) and top five most significantly upregulated genes (**c**) for samples with high EERES comparing with samples with low EERES in the four cancers. The top five most significantly upregulated genes for the MEK_UP.V1_UP enrichment results for OV, UCEC, and CESC were further analyzed to determine their effect on sensitivity to treatment with tamoxifen, fulvestrant, and trametinib using Cancer Cell Line Encyclopedia gene expression and Genomics of Drug Sensitivity in Cancer drug half-maximal inhibitory concentration data. The TPM for genes that were positively correlated with both tamoxifen and fulvestrant sensitivity but negatively correlated with trametinib sensitivity were highlighted. The results of the correlation for gene expression and drug IC50 (presented as natural logarithm of IC50 [LN_IC50]) for fulvestrant (**d**) and trametinib (**e**) are shown as scatter plot with Spearman statistics.

We have shown that the ER expression of ovarian, endometrial, and cervical cancers is correlated with EERES (Fig. 2), and the cancers are dependent on the ER signaling pathway oncogenesis (Fig. 4). This suggests that the gynecologic cancers should be responding to hormonal therapy like breast cancer but not in reality. We further investigated the insensitivity of gynecologic cancers to hormonal therapy by analyzing MSigDB oncogenic pathway enrichment using GSEA in the gynecologic tumor samples with low and high EERESs as shown in Fig. 1a, b. This demonstrated the cross-talks of other pathways enriched in ER signaling–enriched samples for the four female cancers we tested. The most notable results are that the MEK_UP.V1_DN pathway was significantly enriched in breast tumors, whereas the MEK_UP.V1_UP pathway was enriched in ovarian, endometrial, and cervical tumors (Fig. 5a, b). Genes in MEK_UP.V1_DN pathway are down-regulated when MEK is activated. However, several of the genes in the MEK_UP.V1_DN pathway were up-regulated in breast tumors. This means that the MEK pathway was downregulated in breast tumors with high EERESs but activated in ovarian, endometrial, and cervical tumors together with ER signaling. More interestingly, the top five most significantly upregulated genes of the MEK_UP.V1_DN pathway in breast tumors overlapped the significantly upregulated genes in breast tumors shown in Fig. 4c (*GREB1*, *TCC39A*, *ANXA9*, *MYB*, and *PGR*). This suggests that upregulation of ER signaling partly suppresses MEK pathway activity (30/200 genes [15%] in the HALLMARK_ESTROGEN_RESPONSE_EARLY pathway overlap genes in the MEK_UP.V1_DN pathway) but that MEK activation in tumors other than breast tumors might interfere with the activation of the ER-mediated HALLMARK_ESTROGEN_RESPONSE_EARLY pathway by downregulating gene expression in the MEK_UP.V1_DN pathway, thus affecting the EERES enrichment and reducing the correlation of *ESR1* expression with EERES.

To delineate the targets that are involved in the MEK pathway activation and affect endocrine therapy sensitivity of gynecologic cancers, we selected the top five most significantly differentially expressed MEK_UP.V1_UP target genes in the ovarian, endometrial, and cervical tumors combined (*NT5C2*, *CDC42BPB*, *MYOSA*, *PRKCH*, *PCDH1*, *TNFRSF21*, *SCAMP4*, *MPZL2*, *TTC9*, *MALL*, *INAVA*, and *EPHA2* [three genes overlapped in the three tumor types]) (Fig. 5c) for further investigation. Using data from the Cancer Cell Line Encyclopedia and Genomics of Drug Sensitivity in Cancer databases [33, 34], we analyzed the correlation of the individual expression of these genes in Cancer Cell Line Encyclopedia cell lines with the sensitivity of these cell lines to treatment with tamoxifen, fulvestrant, and trametinib. From the 12 selected genes, we performed correlative analysis to identify genes with expression that was significantly positively correlated with both tamoxifen and fulvestrant sensitivity but significantly negatively correlated with trametinib sensitivity. Six genes were identified (*MALL*, *TNFRSF21*, *EPHA2*, *PCDH1*, *MPZL2*, and *INAVA*) (Fig. 5d, e). The Spearman *r* values of the correlations for the six genes for fulvestrant ranged from 0.220 to 0.455, and the *P* values ranged from 6.673e–09 to 4.236e–36. In comparison, the Spearman *r* values of the correlations for the six genes for trametinib ranged from -0.099 to -0.211, and the *P* values ranged from 9.475e–03 to 2.644e–08.

**Fig. 6:**
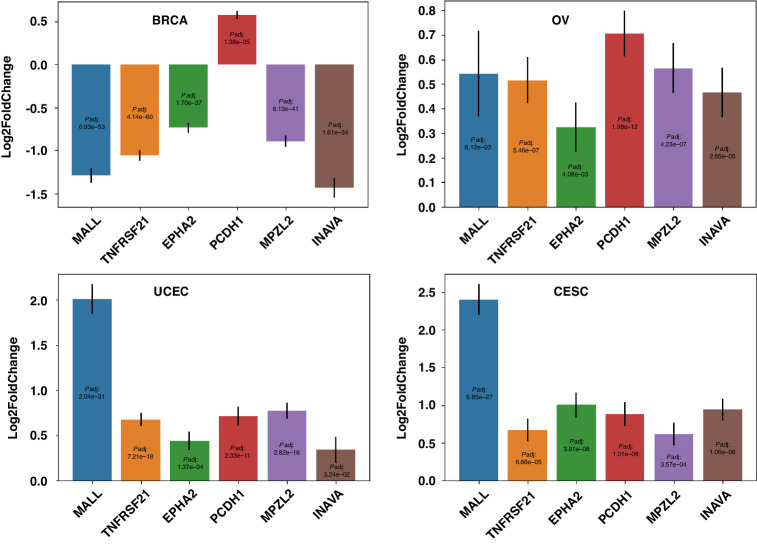
MEK-associated genes are differentially expressed together with ER signaling activity in the female cancers confirmed by DESeq2. The six genes associated with endocrine therapy resistance and MEK inhibitor sensitivity as shown in Fig. 5d were analyzed with their expression level in the four female cancers to confirm their MEK activity between low and high EERES patient tumor samples using DESeq2 as described in methods section. Bar charts of the DESeq2-determined logarithm2 fold change (±SE) (presented as log2FoldChange) and Benjamini-Hochberg adjusted *P* values (padj) for the *EPHA2*, *INAVA*, *MALL*, *MPZL2*, *PCDH1*, and *TNFRSF21* genes comparing the female cancer patients with low and high EERES are shown above.

Given the expression of the six genes shown to be associated with endocrine therapy resistance and MEK inhibitor sensitivity in cancer cell lines (Fig. 5d), we further confirmed the expression fold changes in the tumor samples with high EERES comparing to low EERES with R package DESeq2 for breast, ovarian, endometrial, and cervical cancers. It is because the expression level changes for the six genes comparing samples with low and high EERES in breast cancer is not known from the previous results and the results of ovarian, endometrial, and cervical cancers can also be confirmed by DESeq2. As shown in Fig. 6, five of the genes were significantly downregulated in breast cancer, whereas all six were significantly upregulated in the other three cancers. The downregulation of the five genes in breast cancer further suggests that these genes are the determining factors for hormone therapy responsiveness. This means that the expression of these MEK-associated genes was related to the ER signaling pathway level in patients with gynecologic cancers and may explain the limited responsiveness to endocrine therapy of ovarian, endometrial, and cervical cancer. These genes may be used together with *ESR1* expression and EERES to select patients with high *ESR1* expression and EERES and low MEK activity for endocrine therapy. This possibility warrants further investigation.

## Discussion

Intrinsic endocrine therapy resistance is a challenge in treating gynecologic cancers other than breast cancer [35–37]. In this study, we analyzed the genomic and clinical data on four female cancers (BRCA, OV, UCEC, and CESC) from the TCGA Pan-Cancer Atlas [26] to better understand the correlation of ER expression, ER pathway activation, and alternative pathway activation in patients with these cancers. The use of endocrine therapy for gynecologic cancers is usually based on ER expression according to immunohistochemistry. However, the intrinsic differences in these cancers with different ER expression and ER signaling activity levels remain unknown. This is the first study to decipher the relationship among *ESR1* expression, ER signaling activity, and other activated pathways in ovarian, endometrial, and cervical cancers. We found that the MEK pathway activation is correlated with ER signaling pathway activation in ovarian, endometrial, and cervical cancers.

The prognostic effects of the EERES score (high vs low EERESs) in the breast /endometrium, versus ovary/cervix cohorts are found to be opposite. This could be attributed to their differences in pathological and molecular characteristics. For endometrial cancer, ESR1 expression has been associated with lower grade and stage of the cancer [38]. For ovarian cancer, ESR1 expression has been found to be associated with the loss of chromosome 1p and 16q [39]. Estrogen signaling has also been found to induce genome instability in HPV-induced cervix and promote carcinogenesis in ovarian cancer [40]. Loss of *ESR1* expression has been shown to enhance cervical cancer invasion [41] but how HPV (Human papillomavirus) might dysregulate the expression ER signaling genes will need further investigation [42]. Further studies such as genome stability, mutational profiling, and pathway analysis could be done to identify the molecular aberrancies in different groups. Nevertheless, the activation of the ER signaling pathway and its association with other activated pathways in different female cancers gives us insight about the mechanisms of the carcinogenesis involving ER signaling pathway. Another explanation for the opposite prognostic effect of EERES scores is the difference in the standard of treatment for these patients. For ER+ breast/endometrium, they were treated with endocrine therapies. However, ovary/cervix cancer patients were primarily treated with chemotherapy after surgical resection. It is possible that ESR1/ER signaling could provide certain growth advantages for some of the ovary/cervix cohorts, but endocrine therapies are not commonly used [3].

As described herein, we first used the combination of ER expression and ER signaling (EERES) to predict survival of breast cancer. We chose breast cancer patients given endocrine therapy to determine whether high ER expression and/or high EERES predict improved survival. Improved survival is correlated with improved response to endocrine therapy for breast cancer. Using a small cohort of patients with low-grade serous ovarian carcinoma, we can demonstrate that high EERES could also predict better survival. We asked if any other signaling activity could affect ER signaling in all four female cancers to identify any major factors that contribute to endocrine therapy resistance. Our analyses identified six genes associated with both ER and MEK signaling pathways: five were downregulated in breast cancer, whereas all six were upregulated in ovarian, endometrial, and cervical cancers. More importantly, upregulation of these genes correlated with poor sensitivity to endocrine therapy (tamoxifen and fulvestrant) but good response to treatment with the MEK inhibitor trametinib in cancer cell lines (*n* = 1019). Expression of *EPHA2* had the highest Spearman’s correlation (*r* = 0.455) with fulvestrant resistance (Fig. 5d) and good correlation with tamoxifen resistance (*r* = 0.339, *P* = 1.127e–19). This agrees with results of a previous study demonstrating that overexpression of *EPHA2* can decrease estrogen dependence and tamoxifen sensitivity of the breast cancer cell line MCF7 [43]. Dual targeting of both *EPHA2* and ER has also been proposed for restoring tamoxifen sensitivity in ER/EPHA2-positive breast cancer [44].

Considering the dual activation of both MEK and ER signaling pathways in gynecologic cancers, inhibition of the MEK pathway is a potential approach to sensitizing gynecologic cancers to endocrine therapy. Several MEK inhibitors are approved by the U.S. Food and Drug Administration for treatment of cancers with MEK pathway gene aberrancies, such as melanoma, thyroid cancer, and non-small cell lung cancer. Combination therapies involving MEK inhibitors for cancer treatment are also under development [45–48]. However, as shown in our results, many pathways in addition to the MEK pathway are also upregulated in ovarian, endometrial, and cervical cancers with high ER signaling activity (Fig. 5a), so targeting of more than two pathways may be needed for cancer cell proliferation inhibition.

Previous studies have demonstrated the relationship between estrogen or MEK pathway signaling and prognosis in endometrial and ovarian cancers. The use of MEK inhibitor could reverse antiestrogen resistance in ER+ high grade serous ovarian cancer [49], and estrogen receptor pathway activity is associated with outcome in endometrial cancer [50]. Nevertheless, this is the first study to demonstrate a mechanism of how MEK pathway activation is associated with endocrine therapy resistance of gynecologic cancers by comparing with breast cancers to show the differences in the signaling pathway activation between breast cancers and the gynecologic cancers. The result is also supported by analyzing the EERES signaling, MEK pathway activity and endocrine therapy responses of cell lines used in the Cancer Cell Line Encyclopedia (CCLE). Even though *ESR1* expression might be silenced in many gynecologic cancer cell lines used in the Cancer Cell Line Encyclopedia (CCLE), correlation of genes involved in EERES signaling (which can be dependent or independent of the ESR1 expression) and MEK Pathway activity with endocrine response supports our tissue analysis. In addition, we demonstrated that combined *ESR1* expression and EERES is a good predictor for better survival for hormonal therapy-treated breast cancer patients. This will be helpful in stratifying patients for future endocrine therapy and analysis of response to it. These results provide insight into the development of endocrine therapy and other therapeutic development strategies for gynecologic cancers.

There are limitations regarding the analysis. One of the limitations is the lack of patient risk factor and treatment data. For example, certain risk factors could be associated with EERES. Also, other treatments in the endocrine therapy-treated patients could also complicated the patient outcomes. Another limitation includes the uncertainty in the amount of cancer cells in the tissue. As the estimation of EERES and *ESR1* expression relies on bulk tumor tissue, the proportion of cancer cell and stroma would affect the accuracy of the estimation. With the advancement of spatial transcriptomics, EERES estimation could be further investigated to improve accuracy. For the correlation analysis of anti-estrogen IC50s and gene expression in cell lines, there could also be drawbacks. *ESR1* expressions are commonly downregulated in cell line cultures due to mechanisms such as epigenetic changes and mutations, especially in ovarian cancer cell lines [51, 52]. The correlation analysis might not reflect the situation in the gynecologic cancers.

In conclusion, increased ER expression and ER signaling are both associated with improved hormone therapy responsiveness of breast cancer. MEK pathway activity may co-activate with the ER signaling activity in ovarian, endometrial, and cervical cancers, leading to endocrine therapy resistance.

## Methods

### Data sources

Patient sample data were obtained from the TCGA Pan-Cancer Atlas. Patient survival, reverse-phase protein array, mutation, clinical, and copy number alteration data were downloaded from cBioPortal for Cancer Genomics. For analysis of the Pan-Cancer Atlas patient tumor RNA-seq data, raw gene counts and TPM values were retrieved from the National Cancer Institute Genomic Data Commons Data Portal (https://portal.gdc.cancer.gov). Some data sets may have had missing data; therefore, missing data was assumed to be random, which would not affect the analysis. For analyses of normal tissue RNA-seq data, data generated by the Developmental Genotype-Tissue Expression (dGTEx) project were used. GTEx data release V8 raw gene counts and TPM values for the RNA-seq data were downloaded from the GTEx portal (https://gtexportal.org). Clinical and level 3 gene expression data for the BRCA data set were obtained from cBioPortal. Cancer Cell Line Encyclopedia gene expression TPM data and cell line metadata were downloaded from the DepMap portal (https://depmap.org/portal/download/all/; Public 22Q4 data set). Cell line half-maximal inhibitory concentration data (GDSC2 data set) were downloaded from the website of Genomics of Drug Sensitivity in Cancer (https://www.cancerrxgene.org/downloads/bulk_download).

### RNA-seq of low-grade ovarian tumor samples

All low-grade serous ovarian tumor samples were retrieved from the archives of the Department of Pathology at MD Anderson. Samples were collected, archived, and managed under research protocols approved by the MD Anderson Institutional Review Board. Total RNA was extracted from 12 frozen samples using a QIAGEN RNeasy Mini Kit, and sequencing libraries of the 12 samples were prepared using a KAPA Stranded RNA-Seq Kit (Roche Diagnostics). RNA-seq was performed using an Illumina HiSeq 4000 system. The sequences were aligned to the human reference genome GRCh38, and gene expression was estimated using CLC Genomics Workbench (version 20; QIAGEN).

### Differential gene expression analysis and statistics

Differential gene expression was analyzed using DESeq2 in R [53]. The gene count matrix and labels for two groups aimed for analysis were loaded into DESeq2, and the statistical differences in gene expression between the two groups of patient tumor samples were calculated using the Wald test in DESeq2. The results were presented as log2(fold change) ± SE between the two groups compared.

### RNA expression–based pathway enrichment between-group analysis and statistics

Pathway enrichment analysis was performed using the RNA expression profiles of the two groups patient tumor samples via GSEA with the Python package GSEApy [31, 54]. The raw gene count matrix of the tumor samples was normalized to the trimmed mean of M-values, which is recommended for GSEA analysis by the Broad Institute (https://software.broadinstitute.org/cancer/software/gsea/wiki/index.php/Using_RNA-seq_Datasets_with_GSEA; accessed on May 1, 2023). The trimmed mean of M-values matrix and label data were then loaded into GSEApy. Pathway enrichment analysis of the two groups was performed using gene sets downloaded from MSigDB [30]. The significantly differentially expressed genes between the two groups for the pathway gene sets tested were identified by GSEApy and they were ranked and plotted as heatmap. *P* value lower than 0.05 was considered statistically significant for the pathways tested between the two groups of patient tumor sample compared.

### RNA expression–based single-sample pathway enrichment analysis and statistics

The sample-level pathway enrichment score for the samples of each cancer tumor data set was calculated using R package GSVA [28]. In a pathway-centric manner, a new matrix of GSVA enrichment scores allows for the evaluation of pathway enrichment for each sample as well as application of standard analytical methods such as survival analysis, clustering, functional enrichment, and cross-tissue pathway analysis. To elaborate, a gene expression (log2[TPM + 1]) matrix of the samples and the gene list of the Hallmark gene signature from MSigDb (https://www.gsea-msigdb.org/gsea/msigdb/human/genesets.jsp?collection=H) for analysis were loaded into the R package GSVA for enrichment score estimation for each sample. The enrichment scores with gaussian distribution for the tested pathways for each sample were then determined by GSVA.

### Survival statistics

The survival data for the study patients with their labels were loaded into the Python package kaplanmeier to plot Kaplan-Meier survival curves unless otherwise specified. The statistics of survival time between two groups of patients were determined by a log-rank test. The optimal threshold for survival was determined by finding the threshold with the lowest average log-rank *P* value for DFS and DSS for the two groups of patients. *P* value lower than 0.05 was considered statistically significant for the survival time of the two groups of patients compared.

### Correlation statistics

The correlation between values such as gene expression, drug IC50, and pathway enrichment score was analyzed using the Spearman correlation test [55]. The correlation was determined using *r* values, and the significance was determined according to *P* values which are smaller than 0.05.

### Supplementary information

Supplemental Material

Figure S1

Table S1

Table S2

Table S3

Table S4

Codes used to generate the results

## Data Availability

Processed data are available from the authors upon reasonable request.

## Code availability

An HTML file of Jupyter notebook used to generate the results this study together with the versions of Python and R and their packages used are available.
