# Supplementary material for: The prognostic value of MEK pathway–associated estrogen receptor signaling activity for female cancers
Source: Br J Cancer. 2024 Apr 6;130(11):1875–84. doi: 10.1038/s41416-024-02668-w (PMC11130254; doi:10.1038/s41416-024-02668-w)
Supplement: Supplementary file 1 — Supplemental Material [file 41416_2024_2668_MOESM1_ESM.docx]

**Supplementary Information**

**Supplementary Table S1: The EERESs of the tumor samples for the four cancers analyzed in the TCGA Pan-Cancer Atlas**

**Supplementary Table S2: The EERESs of the breast tumor samples from METABRIC**

**Supplementary Table S3: The distribution of age and stage at diagnosis and histotype between ESR1/EERES groups of in the TCGA-BRCA Pan-Cancer Atlas**

**Supplementary Table S4: The *ESR1* TPMs, EERESs, and overall survival times of the 12 low-grade ovarian tumor samples from MD Anderson Cancer Center**

**Supplementary Fig S1: Scatter plots of the correlation of *ESR1* gene expression level with the corresponding EERES for the study patients.** The correlation between *ESR1* gene expression level and EERES in samples of the breast, ovarian, endometrial, and cervical cancers were determined using Spearman correlation test. The scatter plot and the statistics are shown.
