# Supplementary figures and images for: The prognostic value of MEK pathway–associated estrogen receptor signaling activity for female cancers
Source: Br J Cancer. 2024 Apr 6;130(11):1875–84. doi: 10.1038/s41416-024-02668-w (PMC11130254; doi:10.1038/s41416-024-02668-w)

**ESR1  
vs  
EERES**

**BRCA**

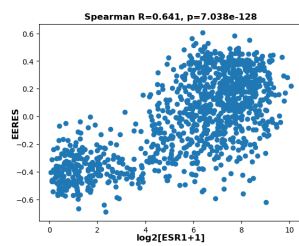

**OV**

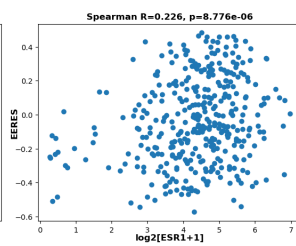

**UCEC**

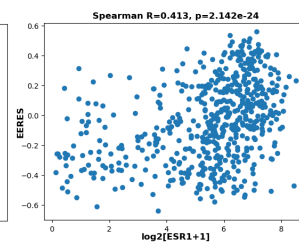

**CESC**

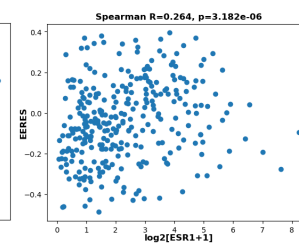

Supplement: Supplementary file 2 — Figure S1 [file 41416_2024_2668_MOESM2_ESM.pdf]
