# Supplementary material for: The prognostic value of MEK pathway–associated estrogen receptor signaling activity for female cancers
Source: Br J Cancer. 2024 Apr 6;130(11):1875–84. doi: 10.1038/s41416-024-02668-w (PMC11130254; doi:10.1038/s41416-024-02668-w)
Supplement: Supplementary file 7 — Codes used to generate the results [file 41416_2024_2668_MOESM7_ESM.html]

 

 
  
 
 Code  
 
 
 
 
 
 
 
 
  
 
  
 
 
 
 
 
 
  
  
 The prognostic value MEK pathway-associated estrogen receptor signaling activity in female cancers ¶   Chun Wai Ng, Yvonne T.M. Tsang, David M. Gershenson, Kwong-Kwok Wong 
 
 
 
  
 
 
 
 
 In [1]: 
 
 
     import   sys 
 print  (  "Python version:"  ,   sys  .  version  ) 
 ! pip   list    |    grep    'pandas\|numpy\|matplotlib\|rpy2\|sklearn\|scipy\|kaplanmeier\|seaborn\|gseapy' 
 import   pandas   as   pd 
 import   numpy   as   np 
 from   scipy   import   stats 
 import   matplotlib.pyplot   as   plt 
 import   kaplanmeier   as   km 
 from   statsmodels.stats.multitest   import   multipletests 
 from   scipy   import   stats 
 from   scipy.stats   import   chi2_contingency  ,   chisquare  ,   spearmanr  ,   ttest_ind 
 import   math 
 import   json 
 import   gseapy   as   gp 
 from   gseapy   import   gseaplot  ,   heatmap  ,   dotplot 
 import   seaborn   as   sns 
 from   sklearn.linear_model   import   LogisticRegression 
 from   sklearn.model_selection   import   train_test_split 
 import   rpy2.robjects   as   ro 
 from   rpy2.robjects.packages   import   importr 
 from   rpy2.robjects   import   pandas2ri 
 from   rpy2.robjects.conversion   import   localconverter 
 from   rpy2.robjects   import   Formula 
 import   rpy2.robjects   as   robjects 
 rinstalled   =   robjects  .  globalenv  .  find  (  "installed.packages"  ) 
 rversion   =   robjects  .  globalenv  .  find  (  "R.Version"  ) 
 rpkgs   =   rinstalled  () 
 rvers   =   rpkgs  .  rx  (  robjects  .  StrVector  ([  "GSVA"  ,  "DESeq2"  ]),   robjects  .  StrVector  ([  "Version"  ])) 
 print  (  rversion  ()  .  rx2  (  "version.string"  )) 
 print  (  "GSVA Version:"  ,   rvers  [  0  ]) 
 print  (  "DESeq2 Version:"  ,   rvers  [  1  ]) 
  
 
 
 
 
 
 
 
 
 
  
 
 Python version: 3.10.12 (main, Nov 20 2023, 15:14:05) [GCC 11.4.0]
geopandas                 0.13.2
gseapy                    1.0.4
kaplanmeier               0.1.9
matplotlib                3.8.1
matplotlib-inline         0.1.6
matplotlib-scalebar       0.8.1
numpy                     1.26.2
numpy-groupies            0.10.2
numpyro                   0.13.2
pandas                    2.1.3
rpy2                      3.5.5
scipy                     1.11.2
seaborn                   0.12.2
[1] "R version 4.3.2 (2023-10-31)"

GSVA Version: 1.48.3
DESeq2 Version: 1.40.2
 
 
 
 
 
  
 
 
 
 
 In [2]: 
 
 
     def   get_cBioportal_survival  (  pfs_path  ,   os_path  ,   dfs_path  ,   dss_path  ): 
     data_pfs   =   pd  .  read_table  (  pfs_path  ,   index_col  =  1  ,   header  =  0  )  .  drop_duplicates  ()  .  dropna  () 
     data_os   =   pd  .  read_table  (  os_path  ,   index_col  =  1  ,   header  =  0  )  .  drop_duplicates  ()  .  dropna  () 
     data_dfs   =   pd  .  read_table  (  dfs_path  ,   index_col  =  1  ,   header  =  0  )  .  drop_duplicates  ()  .  dropna  () 
     data_dss   =   pd  .  read_table  (  dss_path  ,   index_col  =  1  ,   header  =  0  )  .  drop_duplicates  ()  .  dropna  () 

     data_pfs   =   data_pfs  [  ~  data_pfs  .  index  .  duplicated  (  keep  =  "first"  )] 
     data_pfs  [  "PFS_STATUS"  ]   =   [  s  [  0  ]   for   s   in   data_pfs  [  "PFS_STATUS"  ]] 
     data_os  [  "OS_STATUS"  ]   =   [  s  [  0  ]   for   s   in   data_os  [  "OS_STATUS"  ]] 
     data_dfs  [  "DFS_STATUS"  ]   =   [  s  [  0  ]   for   s   in   data_dfs  [  "DFS_STATUS"  ]] 
     data_dss  [  "DSS_STATUS"  ]   =   [  s  [  0  ]   for   s   in   data_dss  [  "DSS_STATUS"  ]] 
     data_dfs   =   data_dfs  [  ~  data_dfs  .  index  .  duplicated  (  keep  =  "first"  )] 
     data_dss   =   data_dss  [  ~  data_dss  .  index  .  duplicated  (  keep  =  "first"  )] 
     data_pfs   =   data_pfs  [  ~  data_pfs  .  index  .  duplicated  (  keep  =  "first"  )] 
     data_os   =   data_os  [  ~  data_os  .  index  .  duplicated  (  keep  =  "first"  )] 
     return   data_pfs  ,   data_os  ,   data_dfs  ,   data_dss 

 def   load_gtex_gene  (  file_location  ): 
     df   =   pd  .  read_table  (  file_location  ,   skiprows  =  2  ,   header  =  0  ,   index_col  =  2  )  .  iloc  [:,   2  :] 
     return   df 

 def   get_gdc_tpm_genecount  (  file_path  ,   meta_file_path  ): 
     ov_gene_meta   =   pd  .  read_table  (  meta_file_path  ,   index_col  =  8  ,   header  =  0  ) 
     ov_gene_filenames   =   [  ov_gene_meta  .  iloc  [  r  ,   12  ]   +   "/"   +   ov_gene_meta  .  iloc  [  r  ,   11  ]   for   r   in   range  (  len  (  ov_gene_meta  ))] 
     ov_gene_caseid   =   ov_gene_meta  .  index 
     ov_gene   =   pd  .  DataFrame  () 
     ov_genecount   =   pd  .  DataFrame  () 
     for   i  ,   f   in   enumerate  (  ov_gene_filenames  ): 
         file   =   pd  .  read_table  (  f  "  {  file_path  }  /  {  f  }  "  ,   skiprows  =  1  ,   header  =  0  ,   index_col  =  1  )  .  iloc  [  4  :] 
         caseid   =   ov_gene_caseid  [  i  ] 
         print  (  caseid  ) 
         tpm   =   file  [[  "tpm_unstranded"  ]] 
         tpm  .  columns   =   [  caseid  ] 
         ov_gene   =   pd  .  concat  ([  ov_gene  ,   tpm  ],   axis  =  1  ) 
         gc   =   file  [[  "unstranded"  ]] 
         gc  .  columns   =   [  caseid  ] 
         ov_genecount   =   pd  .  concat  ([  ov_genecount  ,   gc  ],   axis  =  1  ) 
     ov_gene   =   ov_gene  .  groupby  (  level  =  0  )  .  mean  () 
     q   =   ~  ov_gene  .  columns  .  duplicated  (  keep  =  "first"  ) 
     ov_gene   =   ov_gene  .  loc  [:,  q  ] 
     ov_genecount   =   ov_genecount  .  groupby  (  level  =  0  )  .  mean  () 
     q   =   ~  ov_genecount  .  columns  .  duplicated  (  keep  =  "first"  ) 
     ov_genecount   =   ov_genecount  .  loc  [:,  q  ] 
     return   ov_gene  ,   ov_genecount 

 def   genecount_to_tmm_for_gsea  (  genecount_df  ):  
     import   conorm 
     nf    =   conorm  .  tmm_norm_factors  (  genecount_df  ) 
     gdc_gc_tmm   =   conorm  .  cpm  (  genecount_df  ,   norm_factors  =  nf  ) 
     gdc_gc_tmm  .  insert  (  0  ,   "NAME"  ,   None  ) 
     gdc_gc_tmm  .  reset_index  () 
     gdc_gc_tmm  .  rename  (  columns   =   {  'index'  :  'GENE'  }) 
     return   gdc_gc_tmm 

 def   gsea  (  tmm_df  ,   pheno  ,   genesets  ): 
     gs_res   =   gp  .  gsea  (  data  =  tmm_df  ,   # or data='./P53_resampling_data.txt' 
                      gene_sets  =  genesets  ,   # or enrichr library names 
                      cls  =  pheno  ,   # cls=class_vector 
                      # set permutation_type to phenotype if samples >=15 
                      permutation_type  =  'phenotype'  , 
                      permutation_num  =  1000  ,   # reduce number to speed up test 
                      outdir  =  None  ,    # do not write output to disk 
                      method  =  'signal_to_noise'  , 
                      threads  =  4  ,   seed  =  0  ) 
     return   gs_res 

 def   plotting_gsea  (  gsea_res  ,   geneset_name  ): 
     index   =   gsea_res  .  res2d  [  gsea_res  .  res2d  [  "Term"  ]  ==  geneset_name  ]  .  index  [  0  ] 
     terms   =   gsea_res  .  res2d  .  Term 
     gseaplot  (  gsea_res  .  ranking  ,   term  =  terms  [  index  ],   **  gsea_res  .  results  [  terms  [  index  ]]) 
     ax   =   heatmap  (  df   =   gsea_res  .  heatmat  .  loc  [  gsea_res  .  res2d  .  Lead_genes  [  index  ]  .  split  (  ";"  )],   z_score  =  0  ,   title  =  terms  [  index  ],   figsize  =  (  20  ,  45  ),   xticklabels  =  True  ) 
     ax   =   dotplot  (  gsea_res  .  res2d  , 
          column  =  "FDR q-val"  , 
 #              title='HALLMARK', 
          x  =  "Gene_set"  , 
          cmap  =  plt  .  cm  .  viridis  , 
          size  =  3  ,   # adjust dot size 
          figsize  =  (  4  ,  5  ),   cutoff  =  0.25  ,   show_ring  =  False  ) 
        
        
 def   deseq2_py2r  (  gc_matrix_df  ,   pheno_df  ,   formula  ,   contrast  ): 
     rbase   =   importr  (  'base'  ) 
     deseq2r   =   importr  (  "DESeq2"  ) 
     DESeqDataSetFromMatrix_r   =   robjects  .  globalenv  .  find  (  'DESeqDataSetFromMatrix'  ) 
     mode_r   =   robjects  .  globalenv  .  find  (  'mode'  ) 
     sapply   =   robjects  .  globalenv  .  find  (  'sapply'  ) 
     as_integer_r   =   robjects  .  globalenv  .  find  (  'as.integer'  ) 
     deseq_r   =   robjects  .  globalenv  .  find  (  'DESeq'  ) 
     results_r   =   robjects  .  globalenv  .  find  (  'results'  ) 
     print  (  "Converting df"  ) 
     with   localconverter  (  ro  .  default_converter   +   pandas2ri  .  converter  ): 
         geneExpressionProfile_r   =   ro  .  conversion  .  py2rpy  (  gc_matrix_df  ) 

     with   localconverter  (  ro  .  default_converter   +   pandas2ri  .  converter  ): 
         pheno_r   =   ro  .  conversion  .  py2rpy  (  pheno_df  ) 

     formula_r   =   Formula  (  formula  ) 
     dds_r   =   DESeqDataSetFromMatrix_r  (  countData   =   rbase  .  as_matrix  (  sapply  (  geneExpressionProfile_r  ,   as_integer_r  )), 
                                   colData   =   rbase  .  as_matrix  (  pheno_r  ), 
                                   design   =   formula_r  ) 
     dds_r   =   deseq_r  (  dds_r  ) 
     res_r   =   results_r  (  dds_r  ,   contrast  =  ro  .  StrVector  (  contrast  )) 

     return   pd  .  DataFrame  (  np  .  array  (  rbase  .  as_matrix  (  res_r  )),   index  =  gc_matrix_df  .  index  ,   columns  =  [  "baseMean"  ,  "log2FoldChange"  ,  "lfcSE"  ,  "stat"  ,  "pvalue"  ,   "padj"  ]) 

 def   grouping4  (  esr1_es_df  ,   esr1_threshold  ,   es_threshold  ): 
     groups_label   =   np  .  full  (  len  (  esr1_es_df  ),   "____________________"  ) 

     esr1low   =   esr1_es_df  [  "ESR1"  ]  <=  esr1_threshold 
     eslow   =   esr1_es_df  [  "EARLY"  ]  <=  es_threshold 

     esr1low_eslow_query   =   np  .  logical_and  (  esr1low  ,   eslow  ) 

     groups_label  [  esr1low_eslow_query  ]   =   "ESR1_low_EERES_low" 
 #     print("ESR1_low_EERES_low", len(groups_label[esr1low_eslow_query])) 
     eshigh   =   esr1_es_df  [  "EARLY"  ]  >  es_threshold 
     esr1low_eshigh_query   =   np  .  logical_and  (  esr1low  ,   eshigh  ) 

     groups_label  [  esr1low_eshigh_query  ]   =   "ESR1_low_EERES_high" 
 #     print("ESR1_low_EERES_high", len(groups_label[esr1low_eshigh_query])) 
     esr1high   =   esr1_es_df  [  "ESR1"  ]  >  esr1_threshold 
     esr1high_eslow_query   =   np  .  logical_and  (  esr1high  ,   eslow  ) 

     groups_label  [  esr1high_eslow_query  ]   =   "ESR1_high_EERES_low" 
 #     print("ESR1_high_EERES_low", len(groups_label[esr1high_eslow_query])) 
    
     esr1high_eshigh_query   =   np  .  logical_and  (  esr1high  ,   eshigh  ) 
 #     print(esr1high_eshigh_query.sum()) 
    
     groups_label  [  esr1high_eshigh_query  ]   =   "ESR1_high_EERES_high" 
     esr1_es_df  [  "group"  ]   =   groups_label 
    
     return   esr1_es_df 
    
 def   plotting_4groups  (  survival_esr1_es_df  ,   survivalType  ,   esr1_threshold  ,   es_threshold  ): 

     groups_label   =   np  .  full  (  len  (  survival_esr1_es_df  ),   "____________________"  ) 

     esr1low   =   survival_esr1_es_df  [  "ESR1"  ]  <=  esr1_threshold 
     eslow   =   survival_esr1_es_df  [  "EARLY"  ]  <=  es_threshold 

     esr1low_eslow_query   =   np  .  logical_and  (  esr1low  ,   eslow  ) 

     groups_label  [  esr1low_eslow_query  ]   =   "ESR1_low_EERES_low" 
 #     print("ESR1_low_EERES_low", len(groups_label[esr1low_eslow_query])) 
     eshigh   =   survival_esr1_es_df  [  "EARLY"  ]  >  es_threshold 
     esr1low_eshigh_query   =   np  .  logical_and  (  esr1low  ,   eshigh  ) 

     groups_label  [  esr1low_eshigh_query  ]   =   "ESR1_low_EERES_high" 
 #     print("ESR1_low_EERES_high", len(groups_label[esr1low_eshigh_query])) 
     esr1high   =   survival_esr1_es_df  [  "ESR1"  ]  >  esr1_threshold 
     esr1high_eslow_query   =   np  .  logical_and  (  esr1high  ,   eslow  ) 

     groups_label  [  esr1high_eslow_query  ]   =   "ESR1_high_EERES_low" 
 #     print("ESR1_high_EERES_low", len(groups_label[esr1high_eslow_query])) 
    
     esr1high_eshigh_query   =   np  .  logical_and  (  esr1high  ,   eshigh  ) 
 #     print(esr1high_eshigh_query.sum()) 
     groups_label  [  esr1high_eshigh_query  ]   =   "ESR1_high_EERES_high" 
 #     print("ESR1_high_EERES_high", len(groups_label[esr1high_eshigh_query])) 
    
 #     print(np.unique(groups_label, return_counts=True)) 
 #     print("ESR1_low_EERES_low", survival_esr1_es_df[esr1low_eslow_query][f"{survivalType}_MONTHS"].median()) 
 #     print("ESR1_low_EERES_high", survival_esr1_es_df[esr1low_eshigh_query][f"{survivalType}_MONTHS"].median()) 
 #     print("ESR1_high_EERES_low", survival_esr1_es_df[esr1high_eslow_query][f"{survivalType}_MONTHS"].median()) 
 #     print("ESR1_high_EERES_high", survival_esr1_es_df[esr1high_eshigh_query][f"{survivalType}_MONTHS"].median()) 
 #     kmf = KaplanMeierFitter(label="waltons_data") 
 #     kmf.fit(survival_esr1_es_df[esr1low_eslow_query][f"{survivalType}_MONTHS"], survival_esr1_es_df[esr1low_eslow_query][f"{survivalType}_STATUS"]) 
 #     print("ESR1_low_EERES_low",kmf.median_survival_time_) 
 #     kmf = KaplanMeierFitter(label="waltons_data") 
 #     kmf.fit(survival_esr1_es_df[esr1low_eshigh_query][f"{survivalType}_MONTHS"], survival_esr1_es_df[esr1low_eshigh_query][f"{survivalType}_STATUS"]) 
 #     print("ESR1_low_EERES_high", kmf.median_survival_time_) 
 #     kmf = KaplanMeierFitter(label="waltons_data") 
 #     kmf.fit(survival_esr1_es_df[esr1high_eslow_query][f"{survivalType}_MONTHS"], survival_esr1_es_df[esr1high_eslow_query][f"{survivalType}_STATUS"]) 
 #     print("ESR1_high_EERES_low", kmf.median_survival_time_) 
 #     kmf = KaplanMeierFitter(label="waltons_data") 
 #     kmf.fit(survival_esr1_es_df[esr1high_eshigh_query][f"{survivalType}_MONTHS"], survival_esr1_es_df[esr1high_eshigh_query][f"{survivalType}_STATUS"]) 
 #     print("ESR1_high_EERES_high", kmf.median_survival_time_) 
     result_df   =   pd  .  DataFrame  (  index  =  [  "ESR1_low_EERES_low"  ,   "ESR1_low_EERES_high"  ,   "ESR1_high_EERES_low"  ,   "ESR1_high_EERES_high"  ],   columns  =  [  "ESR1_low_EERES_low"  ,   "ESR1_low_EERES_high"  ,   "ESR1_high_EERES_low"  ,   "ESR1_high_EERES_high"  ]) 
     results1   =   km  .  fit  (  survival_esr1_es_df  [  esr1low_eshigh_query  |  esr1low_eslow_query  ][  f  "  {  survivalType  }  _MONTHS"  ],   survival_esr1_es_df  [  esr1low_eshigh_query  |  esr1low_eslow_query  ][  f  "  {  survivalType  }  _STATUS"  ],   groups_label  [  esr1low_eshigh_query  |  esr1low_eslow_query  ]) 
     print  (  "ESR1_low_EERES_low"  ,   "vs"  ,   "ESR1_low_EERES_high"  ,   results1  [  'logrank_P'  ]) 
     result_df  .  loc  [  "ESR1_low_EERES_low"  ,   "ESR1_low_EERES_high"  ]   =   results1  [  'logrank_P'  ] 
     results1   =   km  .  fit  (  survival_esr1_es_df  [  esr1high_eslow_query  |  esr1low_eslow_query  ][  f  "  {  survivalType  }  _MONTHS"  ],   survival_esr1_es_df  [  esr1high_eslow_query  |  esr1low_eslow_query  ][  f  "  {  survivalType  }  _STATUS"  ],   groups_label  [  esr1high_eslow_query  |  esr1low_eslow_query  ]) 
     print  (  "ESR1_low_EERES_low"  ,   "vs"  ,   "ESR1_high_EERES_low"  ,   results1  [  'logrank_P'  ]) 
     result_df  .  loc  [  "ESR1_low_EERES_low"  ,   "ESR1_high_EERES_low"  ]   =   results1  [  'logrank_P'  ] 
     results3   =   km  .  fit  (  survival_esr1_es_df  [  esr1high_eshigh_query  |  esr1low_eslow_query  ][  f  "  {  survivalType  }  _MONTHS"  ],   survival_esr1_es_df  [  esr1high_eshigh_query  |  esr1low_eslow_query  ][  f  "  {  survivalType  }  _STATUS"  ],   groups_label  [  esr1high_eshigh_query  |  esr1low_eslow_query  ]) 
     print  (  "ESR1_low_EERES_low"  ,   "vs"  ,   "ESR1_high_EERES_high"  ,   results3  [  'logrank_P'  ]) 
     result_df  .  loc  [  "ESR1_low_EERES_low"  ,   "ESR1_high_EERES_high"  ]   =   results3  [  'logrank_P'  ] 
     results2   =   km  .  fit  (  survival_esr1_es_df  [  esr1low_eshigh_query  |  esr1high_eslow_query  ][  f  "  {  survivalType  }  _MONTHS"  ],   survival_esr1_es_df  [  esr1low_eshigh_query  |  esr1high_eslow_query  ][  f  "  {  survivalType  }  _STATUS"  ],   groups_label  [  esr1low_eshigh_query  |  esr1high_eslow_query  ]) 
     print  (  "ESR1_low_EERES_high"  ,   "vs"  ,   "ESR1_high_EERES_low"  ,   results2  [  'logrank_P'  ]) 
     result_df  .  loc  [  "ESR1_low_EERES_high"  ,   "ESR1_high_EERES_low"  ]   =   results2  [  'logrank_P'  ] 
     results2   =   km  .  fit  (  survival_esr1_es_df  [  esr1low_eshigh_query  |  esr1high_eshigh_query  ][  f  "  {  survivalType  }  _MONTHS"  ],   survival_esr1_es_df  [  esr1low_eshigh_query  |  esr1high_eshigh_query  ][  f  "  {  survivalType  }  _STATUS"  ],   groups_label  [  esr1low_eshigh_query  |  esr1high_eshigh_query  ]) 
     print  (  "ESR1_low_EERES_high"  ,   "vs"  ,   "ESR1_high_EERES_high"  ,   results2  [  'logrank_P'  ]) 
     result_df  .  loc  [  "ESR1_low_EERES_high"  ,   "ESR1_high_EERES_high"  ]   =   results2  [  'logrank_P'  ] 
     results4   =   km  .  fit  (  survival_esr1_es_df  [  esr1high_eshigh_query  |  esr1high_eslow_query  ][  f  "  {  survivalType  }  _MONTHS"  ],   survival_esr1_es_df  [  esr1high_eshigh_query  |  esr1high_eslow_query  ][  f  "  {  survivalType  }  _STATUS"  ],   groups_label  [  esr1high_eshigh_query  |  esr1high_eslow_query  ]) 
     print  (  "ESR1_high_EERES_low"  ,   "vs"  ,   "ESR1_high_EERES_high"  ,   results4  [  'logrank_P'  ]) 
     result_df  .  loc  [  "ESR1_high_EERES_low"  ,   "ESR1_high_EERES_high"  ]   =   results4  [  'logrank_P'  ] 
     print  (  result_df  ) 
     results   =   km  .  fit  (  survival_esr1_es_df  [  f  "  {  survivalType  }  _MONTHS"  ],   survival_esr1_es_df  [  f  "  {  survivalType  }  _STATUS"  ],   groups_label  ) 
     km  .  plot  (  results  ,   full_ylim  =  True  ,   y_percentage  =  True  ,   fontsize  =  15  ) 
     plt  .  show  () 

 def   plotting_2groups  (  survival_esr1_es_df  ,   survivalType  ,   esr1_threshold  ,   es_threshold  ): 
     results   =   km  .  fit  (  survival_esr1_es_df  [  f  "  {  survivalType  }  _MONTHS"  ],   survival_esr1_es_df  [  f  "  {  survivalType  }  _STATUS"  ],   (  survival_esr1_es_df  [  "EARLY"  ]  >  es_threshold  )  .  apply  (  lambda   x  :   "High EERES"   if   x   else   "Low EERES"  )) 
     km  .  plot  (  results  ,   full_ylim  =  True  ,   y_percentage  =  True  ,   fontsize  =  15  ) 
     plt  .  show  () 
     results   =   km  .  fit  (  survival_esr1_es_df  [  f  "  {  survivalType  }  _MONTHS"  ],   survival_esr1_es_df  [  f  "  {  survivalType  }  _STATUS"  ],   (  survival_esr1_es_df  [  "ESR1"  ]  >  esr1_threshold  )  .  apply  (  lambda   x  :   "High ESR1"   if   x   else   "Low ESR1"  )) 
     km  .  plot  (  results  ,   full_ylim  =  True  ,   y_percentage  =  True  ,   fontsize  =  15  ) 
     plt  .  show  () 
    
 def   plotting_scatter_esr1_es  (  esr1_es_df  ,   esr1_threshold  ,   es_threshold  ):     
     plt  .  scatter  (  np  .  log2  (  esr1_es_df  [  "ESR1"  ]   +   1  ),   esr1_es_df  [  "EARLY"  ]) 
     plt  .  xlabel  (  "log2[ESR1+1]"  ,   weight  =  "bold"  ,   fontsize  =  12  ,   labelpad  =  0  ) 
     plt  .  ylabel  (  "EERES"  ,   weight  =  "bold"  ,   fontsize  =  12  ,   labelpad  =-  5  ) 

     x   =   (  np  .  log2  (  esr1_threshold  +  1  ),   np  .  log2  (  esr1_threshold  +  1  )) 
     y   =   (  esr1_es_df  [  "EARLY"  ]  .  min  ()   -   0.1  ,   esr1_es_df  [  "EARLY"  ]  .  max  ()   +   0.1  ) 
     plt  .  plot  (  x  ,   y  ,   "--"  ,   color  =  'orange'  ) 
     plt  .  plot  ([  np  .  log2  (  esr1_es_df  [  "ESR1"  ]  +  1  )  .  min  ()   -   0.5  ,   np  .  log2  (  esr1_es_df  [  "ESR1"  ]  +  1  )  .  max  ()   +   0.5  ],   [  es_threshold  ,   es_threshold  ],   "--"  ,   color  =  'orange'  ) 
     plt  .  ylim  ([  esr1_es_df  [  "EARLY"  ]  .  min  ()   -   0.1  ,   esr1_es_df  [  "EARLY"  ]  .  max  ()   +   0.1  ]) 
     plt  .  xlim  ([  np  .  log2  (  esr1_es_df  [  "ESR1"  ]  +  1  )  .  min  ()   -   0.5  ,   np  .  log2  (  esr1_es_df  [  "ESR1"  ]  +  1  )  .  max  ()   +   0.5  ]) 
     sr  ,   sp   =   stats  .  spearmanr  (  np  .  log2  (  esr1_es_df  [  "ESR1"  ]),   esr1_es_df  [  "EARLY"  ]) 
     plt  .  title  (  f  "Spearman R=  {  sr  :  .3f  }  , p=  {  sp  :  .3e  }  "  ,   weight  =  "bold"  ) 
     plt  .  show  () 


 def   groups_clinical  (  gdc_clinical_df  ,   stage_colname  ,   age_colname  ,   grouping  ): 
     clinical_grouping_df   =   gdc_clinical_df  .  join  (  grouping  ,   how  =  'inner'  ) 
     clinical_grouping_df  .  loc  [  clinical_grouping_df  [  stage_colname  ]  ==  "Stage I"  ,   stage_colname  ]   =   "T1" 
     clinical_grouping_df  .  loc  [  clinical_grouping_df  [  stage_colname  ]  ==  "Stage IB"  ,   stage_colname  ]   =   "T1" 
     clinical_grouping_df  .  loc  [  clinical_grouping_df  [  stage_colname  ]  ==  "Stage IC"  ,   stage_colname  ]   =   "T1" 
     clinical_grouping_df  .  loc  [  clinical_grouping_df  [  stage_colname  ]  ==  "Stage IA"  ,   stage_colname  ]   =   "T1" 
     clinical_grouping_df  .  loc  [  clinical_grouping_df  [  stage_colname  ]  ==  "Stage II"  ,   stage_colname  ]   =   "T2" 
     clinical_grouping_df  .  loc  [  clinical_grouping_df  [  stage_colname  ]  ==  "Stage IIA"  ,   stage_colname  ]   =   "T2" 
     clinical_grouping_df  .  loc  [  clinical_grouping_df  [  stage_colname  ]  ==  "Stage IIB"  ,   stage_colname  ]   =   "T2" 
     clinical_grouping_df  .  loc  [  clinical_grouping_df  [  stage_colname  ]  ==  "Stage III"  ,   stage_colname  ]   =   "T3" 
     clinical_grouping_df  .  loc  [  clinical_grouping_df  [  stage_colname  ]  ==  "Stage IIIA"  ,   stage_colname  ]   =   "T3" 
     clinical_grouping_df  .  loc  [  clinical_grouping_df  [  stage_colname  ]  ==  "Stage IIIB"  ,   stage_colname  ]   =   "T3" 
     clinical_grouping_df  .  loc  [  clinical_grouping_df  [  stage_colname  ]  ==  "Stage IIIC"  ,   stage_colname  ]   =   "T3" 
     clinical_grouping_df  .  loc  [  clinical_grouping_df  [  stage_colname  ]  ==  "Stage IV"  ,   stage_colname  ]   =   "T4" 
     clinical_grouping_df  .  loc  [  clinical_grouping_df  [  stage_colname  ]  ==  "Stage IVB"  ,   stage_colname  ]   =   "T4" 
     clinical_grouping_df  .  loc  [  clinical_grouping_df  [  stage_colname  ]  ==  "Stage IVD"  ,   stage_colname  ]   =   "T4" 
     clinical_grouping_df  .  loc  [  clinical_grouping_df  [  stage_colname  ]  ==  "Stage X"  ,   stage_colname  ]   =   "TX" 
     clinical_stage_grouped   =   clinical_grouping_df  .  groupby  ([  "group"  ,   stage_colname  ])  .  count  ()  .  iloc  [:,:  1  ] 
     clinical_stage_count   =   pd  .  DataFrame  (  np  .  zeros  ((  4  ,  4  )),   index   =   [  "ESR1_high_EERES_high"  ,  "ESR1_high_EERES_low"  ,  "ESR1_low_EERES_high"  ,  "ESR1_low_EERES_low"  ],   columns   =   [  "T1"  ,   "T2"  ,   "T3"  ,   "T4"  ]) 
     for   r   in   clinical_stage_grouped  .  iterrows  (): 
         clinical_stage_count  .  loc  [  r  [  0  ][  0  ],   r  [  0  ][  1  ]]   =   r  [  1  ]  .  values  [  0  ] 
        
        
     ages_tmp   =   clinical_grouping_df  [[  age_colname  ]] 
     ages_tmp  .  loc  [  ages_tmp  [  age_colname  ]  ==  "[Not Available]"  ,   age_colname  ]   =   "10000" 
     ages_tmp   =   ages_tmp  .  astype  (  int  ) 
     clinical_grouping_df  .  loc  [((  ages_tmp  [  age_colname  ]  >=  21  )  &  (  ages_tmp  [  age_colname  ]  <=  40  )),   age_colname  ]   =   "21-40" 
     clinical_grouping_df  .  loc  [((  ages_tmp  [  age_colname  ]  >=  41  )  &  (  ages_tmp  [  age_colname  ]  <=  60  )),   age_colname  ]   =   "41-60" 
     clinical_grouping_df  .  loc  [((  ages_tmp  [  age_colname  ]  >=  61  )  &  (  ages_tmp  [  age_colname  ]  <=  80  )),   age_colname  ]   =   "61-80" 
     clinical_grouping_df  .  loc  [((  ages_tmp  [  age_colname  ]  >=  81  )  &  (  ages_tmp  [  age_colname  ]  <=  100  )),   age_colname  ]   =   "81-100" 
     clinical_age_count   =   pd  .  DataFrame  (  np  .  zeros  ((  4  ,  4  )),   index   =   [  "ESR1_high_EERES_high"  ,  "ESR1_high_EERES_low"  ,  "ESR1_low_EERES_high"  ,  "ESR1_low_EERES_low"  ],   columns   =   [  "21-40"  ,   "41-60"  ,   "61-80"  ,   "81-100"  ]) 
     clinical_age_grouped   =   clinical_grouping_df  .  groupby  ([  "group"  ,   age_colname  ])  .  count  () 
     for   r   in   clinical_age_grouped  .  iterrows  (): 
         clinical_age_count  .  loc  [  r  [  0  ][  0  ],   r  [  0  ][  1  ]]   =   r  [  1  ]  .  values  [  0  ] 
     return   clinical_stage_count  ,   clinical_age_count 
    
 def   gsva_py2r  (  geneExpressionProfile  ,   gene_sets  ): 
     rbase   =   importr  (  'base'  ) 
     print  (  "Converting df"  ) 
     with   localconverter  (  ro  .  default_converter   +   pandas2ri  .  converter  ): 
         geneExpressionProfile_r   =   ro  .  conversion  .  py2rpy  (  geneExpressionProfile  ) 
     gene_sets_r   =   ro  .  ListVector  (  gene_sets  ) 
     gsvar   =   importr  (  "GSVA"  ) 
     es   =   gsvar  .  gsva  (  rbase  .  as_matrix  (  geneExpressionProfile_r  ),   gene_sets_r  ) 
     es_df   =   pd  .  DataFrame  (  np  .  array  (  es  .  transpose  ()),   index  =  es  .  colnames  ,   columns  =  es  .  rownames  ) 
     return   es_df 
    
 def   ERs_vs_EERES_table  (  ERs_vs_EERES_df  ): 
     df   =   pd  .  DataFrame  (  index  =  [  "ESR1"  ,  "ESR2"  ,  "ESRRA"  ,  "ESRRB"  ,  "ESRRG"  ,  "GPER1"  ],   columns  =  [  "R"  ,  "p"  ]) 
     for   er   in   [  "ESR1"  ,  "ESR2"  ,  "ESRRA"  ,  "ESRRB"  ,  "ESRRG"  ,  "GPER1"  ]: 
         s  ,   p   =   stats  .  spearmanr  (  np  .  log2  (  ERs_vs_EERES_df  [  er  ]  +  1  ),   ERs_vs_EERES_df  [  "EARLY"  ]) 
         p   =   f  "  {  p  :  .2e  }  " 
         df  .  loc  [  er  ,  "R"  ]   =   s 
         df  .  loc  [  er  ,  "p"  ]   =   p 
     return   df 
  
 
 
 
 
 
 
 
 
 
  
  
 Loading gene expression for GTEx (breast, ovary, uterus, cervix) and survival and gene expression for TCGA (BRCA, OV, UCEC, CESC) ¶  
 
 
 
  
 
 
 
 
 In [3]: 
 
 
     pfs_path   =   "Data/gdc/TCGA-BRCA/clinical/cBioportal/KM_Plot__Progression_Free__Survival_(months).txt" 
 os_path   =   "Data/gdc/TCGA-BRCA/clinical/cBioportal/KM_Plot__Overall_Survival__(months).txt" 
 dfs_path   =   "Data/gdc/TCGA-BRCA/clinical/cBioportal/KM_Plot__Disease_Free__Survival_(months).txt" 
 dss_path   =   "Data/gdc/TCGA-BRCA/clinical/cBioportal/KM_Plot__Disease-specific_Survival__(months).txt" 
 brca_pfs  ,   brca_os  ,   brca_dfs  ,   brca_dss   =   get_cBioportal_survival  (  pfs_path  ,   os_path  ,   dfs_path  ,   dss_path  ) 

 pfs_path   =   "Data/gdc/TCGA-OV/Clinical/cBioportal/KM_Plot__Progression_Free__Survival_(months).txt" 
 os_path   =   "Data/gdc/TCGA-OV/Clinical/cBioportal/KM_Plot__Overall_Survival__(months).txt" 
 dfs_path   =   "Data/gdc/TCGA-OV/Clinical/cBioportal/KM_Plot__Disease_Free__Survival_(months).txt" 
 dss_path   =   "Data/gdc/TCGA-OV/Clinical/cBioportal/KM_Plot__Disease-specific_Survival__(months).txt" 
 ov_pfs  ,   ov_os  ,   ov_dfs  ,   ov_dss   =   get_cBioportal_survival  (  pfs_path  ,   os_path  ,   dfs_path  ,   dss_path  ) 

 pfs_path   =   "Data/gdc/TCGA-UCEC/Clinical/cBioportal/KM_Plot__Progression_Free__Survival_(months).txt" 
 os_path   =   "Data/gdc/TCGA-UCEC/Clinical/cBioportal/KM_Plot__Overall_Survival__(months).txt" 
 dfs_path   =   "Data/gdc/TCGA-UCEC/Clinical/cBioportal/KM_Plot__Disease_Free__Survival_(months).txt" 
 dss_path   =   "Data/gdc/TCGA-UCEC/Clinical/cBioportal/KM_Plot__Disease-specific_Survival__(months).txt" 
 ucec_pfs  ,   ucec_os  ,   ucec_dfs  ,   ucec_dss   =   get_cBioportal_survival  (  pfs_path  ,   os_path  ,   dfs_path  ,   dss_path  ) 

 pfs_path   =   "Data/gdc/TCGA-CESC/Clinical/cBioportal/KM_Plot__Progression_Free__Survival_(months).txt" 
 os_path   =   "Data/gdc/TCGA-CESC/Clinical/cBioportal/KM_Plot__Overall_Survival__(months).txt" 
 dfs_path   =   "Data/gdc/TCGA-CESC/Clinical/cBioportal/KM_Plot__Disease_Free__Survival_(months).txt" 
 dss_path   =   "Data/gdc/TCGA-CESC/Clinical/cBioportal/KM_Plot__Disease-specific_Survival__(months).txt" 
 cesc_pfs  ,   cesc_os  ,   cesc_dfs  ,   cesc_dss   =   get_cBioportal_survival  (  pfs_path  ,   os_path  ,   dfs_path  ,   dss_path  ) 
  
 
 
 
 
  
 
 
 
 
 In [4]: 
 
 
     # Load GTEx and TCGA breast, ovary, uterine, cervix gene expression 

 brca_gdc_tpm  ,   brca_gdc_genecount   =   get_gdc_tpm_genecount  (  "Data/gdc/TCGA-BRCA/Gene Expression/download"  ,   "Data/gdc/TCGA-BRCA/Gene Expression/File_metadata.txt"  ) 
 brca_gtex_gc   =   load_gtex_gene  (  "Data/GTEx/gene_reads_2017-06-05_v8_breast_mammary_tissue.gct.txt"  )  .  groupby  (  level  =  0  )  .  mean  () 
 brca_gtex_gdc_gc   =   brca_gtex_gc  .  join  (  brca_gdc_genecount  ,   how  =  'inner'  ) 
 brca_gtex_gdc_pheno   =   np  .  array  ([  "Normal"   for   i   in   range  (  len  (  brca_gtex_gc  .  columns  ))]   +   [  "Cancer"   for   i   in   range  (  len  (  brca_gdc_genecount  .  columns  ))]) 
 brca_gtex_gdc_pheno_df   =   pd  .  DataFrame  (  brca_gtex_gdc_pheno  ,   index  =  brca_gtex_gdc_gc  .  columns  ,   columns  =  [  "type"  ]) 
 brca_gtex_gdc_tmm   =   genecount_to_tmm_for_gsea  (  brca_gtex_gdc_gc  ) 
 brca_gtex_tpm_location   =   "Data/GTEx/gene_tpm_2017-06-05_v8_breast_mammary_tissue.gct.txt" 
 brca_gtex_tpm   =   load_gtex_gene  (  brca_gtex_tpm_location  ) 

 ov_gdc_tpm  ,   ov_gdc_genecount   =   get_gdc_tpm_genecount  (  "Data/gdc/TCGA-OV/Gene Expression/download"  ,   "Data/gdc/TCGA-OV/Gene Expression/File_metadata.txt"  ) 
 ov_gtex_gc   =   load_gtex_gene  (  "Data/GTEx/gene_reads_2017-06-05_v8_ovary.gct.txt"  )  .  groupby  (  level  =  0  )  .  mean  () 
 ov_gtex_gdc_gc   =   ov_gtex_gc  .  join  (  ov_gdc_genecount  ,   how  =  'inner'  ) 
 ov_gtex_gdc_pheno   =   np  .  array  ([  "Normal"   for   i   in   range  (  len  (  ov_gtex_gc  .  columns  ))]   +   [  "Cancer"   for   i   in   range  (  len  (  ov_gdc_genecount  .  columns  ))]) 
 ov_gtex_gdc_pheno_df   =   pd  .  DataFrame  (  ov_gtex_gdc_pheno  ,   index  =  ov_gtex_gdc_gc  .  columns  ,   columns  =  [  "type"  ]) 
 ov_gtex_gdc_tmm   =   genecount_to_tmm_for_gsea  (  ov_gtex_gdc_gc  ) 
 ov_gtex_tpm_location   =   "Data/GTEx/gene_tpm_2017-06-05_v8_ovary.gct.txt" 
 ov_gtex_tpm   =   load_gtex_gene  (  ov_gtex_tpm_location  ) 

 ucec_gdc_tpm  ,   ucec_gdc_genecount   =   get_gdc_tpm_genecount  (  "Data/gdc/TCGA-UCEC/Gene Expression/download"  ,   "Data/gdc/TCGA-UCEC/Gene Expression/File_metadata.txt"  ) 
 ucec_gtex_gc   =   load_gtex_gene  (  "Data/GTEx/gene_tpm_2017-06-05_v8_uterus.gct.txt"  )  .  groupby  (  level  =  0  )  .  mean  () 
 ucec_gtex_gdc_gc   =   ucec_gtex_gc  .  join  (  ucec_gdc_genecount  ,   how  =  'inner'  ) 
 ucec_gtex_gdc_pheno   =   np  .  array  ([  "Normal"   for   i   in   range  (  len  (  ucec_gtex_gc  .  columns  ))]   +   [  "Cancer"   for   i   in   range  (  len  (  ucec_gdc_genecount  .  columns  ))]) 
 ucec_gtex_gdc_pheno_df   =   pd  .  DataFrame  (  ucec_gtex_gdc_pheno  ,   index  =  ucec_gtex_gdc_gc  .  columns  ,   columns  =  [  "type"  ]) 
 ucec_gtex_gdc_tmm   =   genecount_to_tmm_for_gsea  (  ucec_gtex_gdc_gc  ) 
 ucec_gtex_tpm_location   =   "Data/GTEx/gene_tpm_2017-06-05_v8_uterus.gct.txt" 
 ucec_gtex_tpm   =   load_gtex_gene  (  ucec_gtex_tpm_location  ) 

 cesc_gdc_tpm  ,   cesc_gdc_genecount   =   get_gdc_tpm_genecount  (  "Data/gdc/TCGA-CESC/Gene Expression/download"  ,   "Data/gdc/TCGA-CESC/Gene Expression/File_metadata.txt"  ) 
 cesc_gtex_gc   =   load_gtex_gene  (  "Data/GTEx/gene_reads_2017-06-05_v8_cervix_endocervix.gct.txt"  )  .  groupby  (  level  =  0  )  .  mean  () 
 cesc_gtex_gdc_gc   =   cesc_gtex_gc  .  join  (  cesc_gdc_genecount  ,   how  =  'inner'  ) 
 cesc_gtex_gdc_pheno   =   np  .  array  ([  "Normal"   for   i   in   range  (  len  (  cesc_gtex_gc  .  columns  ))]   +   [  "Cancer"   for   i   in   range  (  len  (  cesc_gdc_genecount  .  columns  ))]) 
 cesc_gtex_gdc_pheno_df   =   pd  .  DataFrame  (  cesc_gtex_gdc_pheno  ,   index  =  cesc_gtex_gdc_gc  .  columns  ,   columns  =  [  "type"  ]) 
 cesc_gtex_gdc_tmm   =   genecount_to_tmm_for_gsea  (  cesc_gtex_gdc_gc  ) 
 cesc_gtex_tpm_location   =   "Data/GTEx/gene_tpm_2017-06-05_v8_cervix_endocervix.gct.txt" 
 cesc_gtex_tpm   =   load_gtex_gene  (  cesc_gtex_tpm_location  ) 
  
 
 
 
 
 
 
 
 
 
  
 
 TCGA-E2-A1L7
TCGA-E2-A1L7
TCGA-AR-A0U0
TCGA-BH-A28O
TCGA-A2-A0D4
TCGA-E9-A1R4
TCGA-AO-A1KQ
TCGA-AC-A62V
TCGA-D8-A143
TCGA-A2-A0SV
TCGA-AN-A0XW
TCGA-D8-A1XV
TCGA-A2-A4RW
TCGA-A7-A0CD
TCGA-E2-A1IG
TCGA-D8-A1XB
TCGA-C8-A134
TCGA-BH-A0BS
TCGA-AR-A2LE
TCGA-A2-A0CO
TCGA-E9-A1NA
TCGA-AN-A0AK
TCGA-E9-A1NA
TCGA-A7-A0DA
TCGA-E2-A572
TCGA-A2-A259
TCGA-BH-A28Q
TCGA-E2-A1IO
TCGA-AQ-A7U7
TCGA-AN-A0FD
TCGA-A8-A07G
TCGA-AO-A0JL
TCGA-B6-A0IM
TCGA-B6-A0IP
TCGA-GM-A2DF
TCGA-A2-A25B
TCGA-BH-A0B0
TCGA-AO-A0JD
TCGA-AN-A0FL
TCGA-E2-A14V
TCGA-AN-A0FF
TCGA-C8-A138
TCGA-E2-A14R
TCGA-AC-A2BM
TCGA-A1-A0SP
TCGA-A2-A0CQ
TCGA-A8-A08J
TCGA-BH-A6R8
TCGA-E9-A1QZ
TCGA-A8-A0AB
TCGA-BH-A0H9
TCGA-AC-A3W7
TCGA-B6-A0IE
TCGA-A8-A07I
TCGA-BH-A0BQ
TCGA-LD-A9QF
TCGA-BH-A18T
TCGA-A7-A26G
TCGA-BH-A0H7
TCGA-D8-A1XG
TCGA-BH-A0E0
TCGA-E2-A14U
TCGA-BH-A0E0
TCGA-S3-AA10
TCGA-BH-A0B7
TCGA-A8-A076
TCGA-B6-A0RN
TCGA-E9-A244
TCGA-E2-A1LK
TCGA-LL-A5YL
TCGA-A8-A06Y
TCGA-BH-A0AZ
TCGA-B6-A0X0
TCGA-EW-A1P4
TCGA-BH-A0BG
TCGA-D8-A1JD
TCGA-BH-A18K
TCGA-D8-A27F
TCGA-A8-A09I
TCGA-A2-A0ST
TCGA-BH-A1FH
TCGA-A7-A0D9
TCGA-B6-A0IN
TCGA-E2-A15K
TCGA-E2-A15K
TCGA-E2-A14Y
TCGA-LD-A7W5
TCGA-B6-A0I5
TCGA-A2-A3XV
TCGA-A2-A0SX
TCGA-EW-A1OY
TCGA-AN-A0FJ
TCGA-AR-A24Z
TCGA-D8-A1XT
TCGA-B6-A0RL
TCGA-E9-A22E
TCGA-BH-A18P
TCGA-E2-A15H
TCGA-E9-A1NG
TCGA-D8-A1JK
TCGA-AC-A7VB
TCGA-AO-A0J2
TCGA-BH-A0B8
TCGA-D8-A27N
TCGA-AC-A8OS
TCGA-BH-A0DO
TCGA-E2-A14Z
TCGA-E2-A1BC
TCGA-AR-A256
TCGA-E2-A1BC
TCGA-A7-A426
TCGA-A8-A08B
TCGA-E2-A15C
TCGA-AC-A5XS
TCGA-OL-A6VR
TCGA-BH-A18Q
TCGA-AR-A24R
TCGA-BH-A0DG
TCGA-EW-A1PG
TCGA-A8-A08H
TCGA-BH-A0DD
TCGA-E2-A1B4
TCGA-D8-A27H
TCGA-E2-A1BD
TCGA-AO-A03T
TCGA-B6-A1KN
TCGA-A7-A5ZV
TCGA-BH-A2L8
TCGA-A2-A0YH
TCGA-D8-A1JI
TCGA-AR-A1AH
TCGA-BH-A0HW
TCGA-EW-A1J3
TCGA-A7-A5ZX
TCGA-EW-A423
TCGA-A2-A25E
TCGA-E2-A153
TCGA-C8-A26Y
TCGA-OL-A66H
TCGA-AQ-A04L
TCGA-BH-A0WA
TCGA-BH-A1EX
TCGA-A8-A08C
TCGA-BH-A0C3
TCGA-BH-A0DE
TCGA-AN-A0AS
TCGA-BH-A0GY
TCGA-AR-A1AN
TCGA-BH-A18S
TCGA-AC-A23H
TCGA-AC-A23H
TCGA-OL-A5RZ
TCGA-E2-A15M
TCGA-E9-A1RC
TCGA-GM-A2DN
TCGA-B6-A0I1
TCGA-EW-A1IW
TCGA-BH-A42V
TCGA-AR-A251
TCGA-AC-A8OP
TCGA-D8-A1XU
TCGA-BH-A18L
TCGA-B6-A0IK
TCGA-BH-A18L
TCGA-BH-A0B1
TCGA-A8-A06Q
TCGA-D8-A27R
TCGA-E9-A22G
TCGA-AO-A03N
TCGA-E2-A1LE
TCGA-3C-AAAU
TCGA-E2-A1IG
TCGA-LL-A9Q3
TCGA-AR-A2LM
TCGA-BH-A0HA
TCGA-AR-A0TS
TCGA-BH-A0HA
TCGA-AR-A24T
TCGA-D8-A1JP
TCGA-A8-A06U
TCGA-PE-A5DC
TCGA-BH-A0DI
TCGA-D8-A1XL
TCGA-S3-AA11
TCGA-AO-A0JJ
TCGA-B6-A1KI
TCGA-A1-A0SB
TCGA-3C-AALJ
TCGA-A2-A0CR
TCGA-D8-A1JG
TCGA-AN-A0FZ
TCGA-BH-A0BZ
TCGA-A8-A095
TCGA-BH-A1FE
TCGA-BH-A1FE
TCGA-A8-A07F
TCGA-BH-A1EN
TCGA-A2-A0EO
TCGA-A2-A3XT
TCGA-D8-A1Y2
TCGA-BH-A1FB
TCGA-BH-A1FB
TCGA-A7-A26F
TCGA-E9-A5UP
TCGA-A2-A1G0
TCGA-BH-A0H0
TCGA-BH-A0H9
TCGA-PL-A8LV
TCGA-BH-A209
TCGA-AO-A1KS
TCGA-BH-A0C0
TCGA-A8-A0A7
TCGA-BH-A0BF
TCGA-E9-A1RI
TCGA-BH-A0DP
TCGA-AC-A62X
TCGA-A8-A083
TCGA-A8-A08L
TCGA-D8-A146
TCGA-E2-A1IJ
TCGA-A1-A0SG
TCGA-D8-A1XC
TCGA-AO-A0J5
TCGA-BH-A0B5
TCGA-BH-A0B5
TCGA-3C-AALK
TCGA-AO-A0J9
TCGA-A2-A3KD
TCGA-C8-A12Q
TCGA-A8-A0A2
TCGA-C8-A1HK
TCGA-A2-A0CV
TCGA-D8-A27I
TCGA-EW-A424
TCGA-EW-A6S9
TCGA-D8-A1X6
TCGA-BH-A1F6
TCGA-BH-A0BL
TCGA-EW-A1J5
TCGA-AN-A046
TCGA-EW-A1IX
TCGA-E9-A1R5
TCGA-E2-A1L6
TCGA-AO-A0JC
TCGA-E2-A1LB
TCGA-E9-A54Y
TCGA-GM-A3XG
TCGA-EW-A2FW
TCGA-AR-A24S
TCGA-OL-A97C
TCGA-AR-A2LL
TCGA-D8-A1XA
TCGA-E2-A1B5
TCGA-E2-A15A
TCGA-E2-A14N
TCGA-A7-A3RF
TCGA-A2-A0D2
TCGA-D8-A1JA
TCGA-E2-A10C
TCGA-A2-A0EX
TCGA-BH-A0DT
TCGA-A7-A0CE
TCGA-A7-A0CE
TCGA-A2-A4S2
TCGA-AR-A5QM
TCGA-AR-A24W
TCGA-AC-A3TM
TCGA-4H-AAAK
TCGA-A7-A0CG
TCGA-BH-A18N
TCGA-D8-A27E
TCGA-A8-A06O
TCGA-C8-A8HQ
TCGA-A7-A26J
TCGA-B6-A0RU
TCGA-BH-A18V
TCGA-BH-A18V
TCGA-HN-A2NL
TCGA-E2-A1II
TCGA-AR-A24O
TCGA-A7-A26E
TCGA-GM-A2D9
TCGA-A8-A07W
TCGA-A2-A04W
TCGA-D8-A1XY
TCGA-EW-A1P7
TCGA-A8-A093
TCGA-AC-A2FB
TCGA-C8-A137
TCGA-E9-A1N3
TCGA-BH-A0DQ
TCGA-BH-A0DQ
TCGA-AC-A3BB
TCGA-C8-A1HJ
TCGA-B6-A0IO
TCGA-C8-A1HL
TCGA-A8-A07Z
TCGA-A2-A3XU
TCGA-A2-A0T3
TCGA-AN-A0XT
TCGA-AC-A62Y
TCGA-GM-A2DB
TCGA-A8-A07S
TCGA-BH-A204
TCGA-A2-A0D1
TCGA-AN-A0AT
TCGA-B6-A0IJ
TCGA-E2-A15E
TCGA-A8-A0A4
TCGA-A8-A09T
TCGA-A2-A3XY
TCGA-BH-A18U
TCGA-A8-A06Z
TCGA-A2-A3KC
TCGA-BH-A0H7
TCGA-BH-A0B7
TCGA-AR-A1AJ
TCGA-OL-A66O
TCGA-AC-A3YI
TCGA-BH-A0BT
TCGA-BH-A0AZ
TCGA-AO-A1KT
TCGA-AR-A0TT
TCGA-BH-A1FL
TCGA-B6-A409
TCGA-E9-A248
TCGA-A2-A3Y0
TCGA-BH-A1FH
TCGA-A7-A3IZ
TCGA-LL-A6FR
TCGA-E9-A22H
TCGA-A7-A0D9
TCGA-A1-A0SI
TCGA-AN-A0XO
TCGA-BH-A0HF
TCGA-B6-A0WS
TCGA-AR-A250
TCGA-S3-A6ZG
TCGA-BH-A1EU
TCGA-GI-A2C9
TCGA-GI-A2C9
TCGA-A2-A0CZ
TCGA-E9-A243
TCGA-EW-A2FS
TCGA-A7-A0CJ
TCGA-BH-A0BJ
TCGA-E2-A1LH
TCGA-E2-A1LH
TCGA-E2-A159
TCGA-LL-A7T0
TCGA-C8-A8HR
TCGA-D8-A1JF
TCGA-C8-A1HG
TCGA-GM-A2DC
TCGA-D8-A1JS
TCGA-A7-A4SE
TCGA-E9-A226
TCGA-E2-A15G
TCGA-BH-A208
TCGA-D8-A1JN
TCGA-AO-A03V
TCGA-D8-A3Z6
TCGA-BH-A0DL
TCGA-BH-A0DL
TCGA-GM-A3NY
TCGA-B6-A402
TCGA-E2-A153
TCGA-BH-A0E9
TCGA-E2-A1IK
TCGA-A2-A0YJ
TCGA-E2-A10A
TCGA-A8-A08I
TCGA-GM-A2DM
TCGA-A1-A0SM
TCGA-A2-A0CY
TCGA-A2-A25C
TCGA-A8-A0A9
TCGA-BH-A1FM
TCGA-BH-A0C3
TCGA-C8-A1HI
TCGA-OL-A5S0
TCGA-AQ-A54N
TCGA-AO-A0JG
TCGA-AO-A12G
TCGA-A2-A0T7
TCGA-B6-A0RP
TCGA-GM-A2DH
TCGA-BH-A0B2
TCGA-D8-A1XF
TCGA-A2-A0CK
TCGA-E9-A247
TCGA-EW-A6SD
TCGA-A7-A4SB
TCGA-E9-A1R2
TCGA-AR-A24Q
TCGA-A8-A091
TCGA-OL-A6VQ
TCGA-GI-A2C8
TCGA-GI-A2C8
TCGA-E2-A108
TCGA-B6-A0X5
TCGA-BH-A1FD
TCGA-BH-A0W5
TCGA-AO-A03U
TCGA-AC-A2FG
TCGA-BH-A0BM
TCGA-BH-A0BM
TCGA-AC-A2FF
TCGA-AC-A2FF
TCGA-AR-A24M
TCGA-E9-A6HE
TCGA-BH-A0EI
TCGA-AC-A4ZE
TCGA-E9-A22A
TCGA-A8-A08R
TCGA-AR-A0TW
TCGA-A8-A082
TCGA-BH-A5J0
TCGA-E2-A1B1
TCGA-C8-A1HN
TCGA-D8-A27G
TCGA-A2-A0CL
TCGA-GM-A5PV
TCGA-D8-A1XD
TCGA-AC-A3HN
TCGA-B6-A0WX
TCGA-B6-A0RT
TCGA-EW-A1J2
TCGA-C8-A278
TCGA-BH-A1F8
TCGA-D8-A27W
TCGA-A2-A1FW
TCGA-AC-A23E
TCGA-E2-A14S
TCGA-AR-A1AS
TCGA-D8-A27K
TCGA-BH-A209
TCGA-AR-A24K
TCGA-AC-A2QH
TCGA-A8-A081
TCGA-AR-A2LJ
TCGA-W8-A86G
TCGA-A8-A09R
TCGA-A1-A0SK
TCGA-E2-A574
TCGA-C8-A131
TCGA-EW-A1P8
TCGA-B6-A0WY
TCGA-AC-A6IV
TCGA-BH-A0B4
TCGA-AC-A2FK
TCGA-A8-A08T
TCGA-AO-A12H
TCGA-GM-A2DK
TCGA-BH-A1F6
TCGA-A2-A0EY
TCGA-E9-A5UO
TCGA-AN-A0XP
TCGA-A7-A4SF
TCGA-E9-A1N6
TCGA-AO-A0J6
TCGA-A8-A09K
TCGA-B6-A0RO
TCGA-BH-A1ES
TCGA-BH-A1ES
TCGA-E2-A1LB
TCGA-A8-A07R
TCGA-A2-A04Q
TCGA-EW-A6SB
TCGA-EW-A3E8
TCGA-A2-A0EV
TCGA-Z7-A8R5
TCGA-A8-A06X
TCGA-BH-A0BV
TCGA-C8-A12X
TCGA-BH-A0DT
TCGA-E2-A15A
TCGA-A8-A08G
TCGA-AR-A0U4
TCGA-B6-A408
TCGA-E2-A1B0
TCGA-BH-A1EW
TCGA-AC-A6IX
TCGA-AC-A6IX
TCGA-B6-A0RG
TCGA-PL-A8LY
TCGA-BH-A18I
TCGA-BH-A18N
TCGA-E9-A24A
TCGA-AN-A04C
TCGA-A1-A0SO
TCGA-A7-A13G
TCGA-A7-A13G
TCGA-C8-A12K
TCGA-E9-A1N4
TCGA-E9-A1N4
TCGA-BH-A0E6
TCGA-AO-A03L
TCGA-A7-A26J
TCGA-A7-A26J
TCGA-V7-A7HQ
TCGA-GM-A3XL
TCGA-E2-A1IU
TCGA-D8-A1JE
TCGA-LL-A440
TCGA-AO-A128
TCGA-A2-A0T1
TCGA-AO-A0J8
TCGA-B6-A0I6
TCGA-D8-A1JC
TCGA-C8-A1HF
TCGA-BH-A5IZ
TCGA-AN-A0AL
TCGA-E9-A54X
TCGA-A2-A0ES
TCGA-A8-A084
TCGA-AC-A2B8
TCGA-D8-A1JM
TCGA-AN-A0XU
TCGA-AR-A5QN
TCGA-JL-A3YW
TCGA-AO-A03M
TCGA-LL-A7SZ
TCGA-BH-A204
TCGA-A8-A09Q
TCGA-S3-AA12
TCGA-B6-A0RE
TCGA-A2-A0YD
TCGA-D8-A1XZ
TCGA-AN-A0FY
TCGA-A7-A13F
TCGA-BH-A18U
TCGA-AR-A24L
TCGA-E9-A1RE
TCGA-BH-A1F0
TCGA-BH-A1F0
TCGA-A1-A0SJ
TCGA-BH-A8FZ
TCGA-A7-A4SA
TCGA-BH-A0BT
TCGA-BH-A18K
TCGA-OL-A5RV
TCGA-BH-A0EA
TCGA-A7-A5ZW
TCGA-B6-A0IB
TCGA-B6-A0X1
TCGA-AN-A0FX
TCGA-E2-A15K
TCGA-BH-A0B3
TCGA-BH-A0B3
TCGA-5L-AAT1
TCGA-A2-A04N
TCGA-D8-A13Y
TCGA-BH-A1EU
TCGA-EW-A1P5
TCGA-AR-A1AW
TCGA-E2-A15P
TCGA-3C-AALI
TCGA-BH-A18P
TCGA-AC-A6NO
TCGA-A2-A04V
TCGA-E9-A1NG
TCGA-C8-A8HP
TCGA-AR-A24P
TCGA-BH-A1FG
TCGA-C8-A130
TCGA-BH-A1FG
TCGA-A2-A0EW
TCGA-BH-A0B8
TCGA-BH-A0E7
TCGA-D8-A1J9
TCGA-BH-A0DO
TCGA-BH-A0BJ
TCGA-A7-A3J0
TCGA-A8-A08Z
TCGA-A8-A096
TCGA-AR-A1AV
TCGA-B6-A400
TCGA-BH-A18Q
TCGA-E2-A10E
TCGA-BH-A0HB
TCGA-BH-A0DD
TCGA-A8-A097
TCGA-E9-A1R7
TCGA-E9-A1R7
TCGA-LD-A7W6
TCGA-OL-A5D8
TCGA-A8-A08S
TCGA-BH-A1FN
TCGA-A8-A09X
TCGA-BH-A1FN
TCGA-GM-A3NW
TCGA-A2-A4S0
TCGA-BH-A0BO
TCGA-A2-A1G6
TCGA-AN-A0FT
TCGA-D8-A1JB
TCGA-A2-A1G1
TCGA-JL-A3YX
TCGA-BH-A0AV
TCGA-D8-A3Z5
TCGA-A8-A075
TCGA-BH-A1FM
TCGA-D8-A141
TCGA-B6-A0I9
TCGA-BH-A18S
TCGA-AO-A0JM
TCGA-UU-A93S
TCGA-BH-A1EO
TCGA-WT-AB44
TCGA-BH-A8G0
TCGA-E9-A1RC
TCGA-D8-A13Z
TCGA-AR-A0TQ
TCGA-A2-A4RY
TCGA-AC-A3QP
TCGA-BH-A0B9
TCGA-A2-A0EN
TCGA-A2-A0YM
TCGA-A8-A06T
TCGA-E2-A1LI
TCGA-OL-A5RY
TCGA-D8-A1XR
TCGA-A2-A0YT
TCGA-LL-A442
TCGA-B6-A0WV
TCGA-D8-A1JJ
TCGA-BH-A1FD
TCGA-A2-A0EP
TCGA-BH-A0BS
TCGA-E2-A155
TCGA-AR-A252
TCGA-A1-A0SN
TCGA-A8-A099
TCGA-AO-A03O
TCGA-OL-A66K
TCGA-B6-A0RM
TCGA-E2-A15L
TCGA-A7-A6VV
TCGA-BH-A0BZ
TCGA-BH-A1FE
TCGA-BH-A0BW
TCGA-BH-A0BW
TCGA-BH-A1EN
TCGA-E2-A15T
TCGA-C8-A12Y
TCGA-A1-A0SH
TCGA-B6-A0IA
TCGA-A2-A3XX
TCGA-C8-A12V
TCGA-A2-A3XW
TCGA-A2-A0ET
TCGA-B6-A0WT
TCGA-EW-A1PC
TCGA-D8-A1Y3
TCGA-E9-A1NF
TCGA-E9-A1NF
TCGA-BH-A0HL
TCGA-AR-A255
TCGA-UL-AAZ6
TCGA-AR-A0U3
TCGA-C8-A12M
TCGA-BH-A1F8
TCGA-A2-A0EQ
TCGA-A7-A6VX
TCGA-A8-A09W
TCGA-BH-A0W7
TCGA-A8-A08P
TCGA-BH-A0BD
TCGA-E2-A1IN
TCGA-EW-A1PH
TCGA-AR-A2LN
TCGA-B6-A1KC
TCGA-BH-A0BQ
TCGA-BH-A0HN
TCGA-GM-A2DO
TCGA-AO-A12A
TCGA-C8-A274
TCGA-BH-A0C0
TCGA-B6-A0IH
TCGA-E9-A1RI
TCGA-AR-A0U2
TCGA-BH-A0DP
TCGA-AR-A1AL
TCGA-BH-A0GZ
TCGA-XX-A89A
TCGA-A2-A0T2
TCGA-LL-A740
TCGA-E9-A1N6
TCGA-E9-A1NC
TCGA-AR-A24H
TCGA-E2-A15S
TCGA-A8-A09V
TCGA-BH-A18G
TCGA-E2-A15I
TCGA-E2-A15I
TCGA-AC-A2BK
TCGA-B6-A0IC
TCGA-A2-A0SY
TCGA-A8-A07U
TCGA-BH-A0BV
TCGA-C8-A26X
TCGA-A2-A0YI
TCGA-C8-A12T
TCGA-A2-A04X
TCGA-AO-A12C
TCGA-E2-A1IF
TCGA-A2-A0ER
TCGA-BH-A1EW
TCGA-GM-A2DI
TCGA-A7-A0CH
TCGA-A7-A0CH
TCGA-A2-A04Y
TCGA-LL-A441
TCGA-D8-A1XO
TCGA-BH-A1F2
TCGA-BH-A1F2
TCGA-C8-A12W
TCGA-A7-A3IY
TCGA-BH-A18V
TCGA-BH-A0H3
TCGA-LL-A5YP
TCGA-D8-A1XS
TCGA-BH-A42T
TCGA-AN-A0FS
TCGA-A7-A26E
TCGA-AN-A0AM
TCGA-A7-A26E
TCGA-B6-A0IQ
TCGA-BH-A0DX
TCGA-AC-A2FB
TCGA-AO-A0JB
TCGA-A8-A06P
TCGA-OL-A5RW
TCGA-B6-A0I8
TCGA-LL-A73Z
TCGA-BH-A1EY
TCGA-A2-A0YC
TCGA-BH-A0W4
TCGA-GM-A3XN
TCGA-A2-A0T6
TCGA-B6-A0WZ
TCGA-BH-AB28
TCGA-A8-A08F
TCGA-A1-A0SQ
TCGA-E2-A15E
TCGA-AO-A12E
TCGA-E2-A1LL
TCGA-A7-A13F
TCGA-E2-A15J
TCGA-E2-A1IE
TCGA-AC-A7VC
TCGA-EW-A1P3
TCGA-C8-A26Z
TCGA-EW-A3U0
TCGA-AC-A2QJ
TCGA-AR-A0TY
TCGA-BH-A0HU
TCGA-BH-A0DG
TCGA-BH-A208
TCGA-A7-A26H
TCGA-D8-A27T
TCGA-OL-A5RX
TCGA-BH-A6R9
TCGA-BH-A1EO
TCGA-LL-A6FP
TCGA-E2-A15M
TCGA-C8-A12L
TCGA-C8-A273
TCGA-A2-A1FV
TCGA-B6-A0RV
TCGA-D8-A1JT
TCGA-OK-A5Q2
TCGA-D8-A1X7
TCGA-E2-A570
TCGA-A8-A07O
TCGA-E2-A1LA
TCGA-E9-A229
TCGA-A2-A0CS
TCGA-D8-A1Y0
TCGA-E9-A22D
TCGA-LL-A8F5
TCGA-E2-A10F
TCGA-A2-A0YK
TCGA-BH-A0HI
TCGA-A7-A2KD
TCGA-PL-A8LZ
TCGA-MS-A51U
TCGA-BH-A0AY
TCGA-E2-A1LS
TCGA-C8-A3M7
TCGA-A2-A0T0
TCGA-AR-A1AP
TCGA-AC-A3QQ
TCGA-E9-A1RB
TCGA-AN-A0G0
TCGA-AN-A0AJ
TCGA-E2-A15R
TCGA-BH-A0DV
TCGA-D8-A1X9
TCGA-AQ-A54O
TCGA-AR-A2LH
TCGA-BH-A0E1
TCGA-OL-A6VO
TCGA-GM-A4E0
TCGA-BH-A1EV
TCGA-BH-A1EV
TCGA-A2-A4S3
TCGA-AO-A1KR
TCGA-AR-A2LQ
TCGA-E9-A22B
TCGA-A8-A08A
TCGA-D8-A4Z1
TCGA-A2-A1FZ
TCGA-A8-A09Z
TCGA-E2-A10B
TCGA-AC-A6IW
TCGA-A7-A13E
TCGA-A7-A13E
TCGA-A7-A13E
TCGA-A7-A13E
TCGA-A8-A07P
TCGA-B6-A0RI
TCGA-BH-A0HP
TCGA-E2-A56Z
TCGA-BH-A1F5
TCGA-BH-A0DZ
TCGA-AQ-A1H3
TCGA-A8-A079
TCGA-EW-A1PF
TCGA-A2-A0D0
TCGA-BH-A0BC
TCGA-BH-A42U
TCGA-AN-A0FV
TCGA-BH-A0BA
TCGA-BH-A0BA
TCGA-BH-A1ET
TCGA-E2-A14Q
TCGA-AR-A24U
TCGA-BH-A0HK
TCGA-AC-A3W6
TCGA-AR-A24N
TCGA-BH-A0H5
TCGA-E9-A1RG
TCGA-B6-A0X4
TCGA-BH-A0HX
TCGA-AO-A126
TCGA-D8-A27V
TCGA-AN-A04A
TCGA-E9-A1R3
TCGA-AN-A0XV
TCGA-AR-A24V
TCGA-WT-AB41
TCGA-Z7-A8R6
TCGA-D8-A1JU
TCGA-A8-A07B
TCGA-AN-A03X
TCGA-A8-A090
TCGA-AO-A124
TCGA-A2-A0T4
TCGA-A7-A4SC
TCGA-EW-A6SA
TCGA-S3-A6ZH
TCGA-E2-A156
TCGA-BH-A0DK
TCGA-EW-A1J1
TCGA-D8-A1JH
TCGA-E2-A1L9
TCGA-BH-A8FY
TCGA-A8-A08O
TCGA-E2-A9RU
TCGA-C8-A275
TCGA-S3-AA0Z
TCGA-AR-A2LR
TCGA-AQ-A0Y5
TCGA-AR-A0TU
TCGA-A8-A09E
TCGA-EW-A1OZ
TCGA-E2-A1B6
TCGA-LL-A6FQ
TCGA-BH-A0C7
TCGA-LL-A50Y
TCGA-B6-A0RS
TCGA-B6-A3ZX
TCGA-AC-A5XU
TCGA-AN-A0XS
TCGA-D8-A1XK
TCGA-E9-A1RB
TCGA-AO-A0J7
TCGA-E9-A3X8
TCGA-A8-A09N
TCGA-BH-A0AU
TCGA-OL-A66J
TCGA-AR-A1AU
TCGA-D8-A73U
TCGA-OL-A66I
TCGA-AO-A0JE
TCGA-A8-A092
TCGA-AR-A254
TCGA-A8-A09G
TCGA-A8-A09B
TCGA-D8-A1XQ
TCGA-LD-A74U
TCGA-AO-A0J3
TCGA-HN-A2OB
TCGA-AR-A1AO
TCGA-BH-A0BR
TCGA-A8-A09M
TCGA-E9-A1RD
TCGA-E9-A1RD
TCGA-A8-A0A1
TCGA-E9-A228
TCGA-BH-A1FC
TCGA-E9-A1N9
TCGA-E9-A1N9
TCGA-C8-A12U
TCGA-C8-A12N
TCGA-A8-A094
TCGA-OL-A66P
TCGA-E9-A249
TCGA-AN-A0XL
TCGA-BH-A0DZ
TCGA-B6-A40C
TCGA-A2-A4S1
TCGA-AN-A041
TCGA-A2-A3XZ
TCGA-E9-A295
TCGA-C8-A12O
TCGA-C8-A132
TCGA-B6-A0I2
TCGA-A2-A0CW
TCGA-E9-A1RA
TCGA-AN-A049
TCGA-A1-A0SF
TCGA-AC-A3W5
TCGA-S3-AA17
TCGA-BH-A1ET
TCGA-5T-A9QA
TCGA-E9-A227
TCGA-E2-A3DX
TCGA-BH-A0HK
TCGA-PE-A5DE
TCGA-BH-A0RX
TCGA-A7-A0DC
TCGA-A2-A04U
TCGA-BH-A18J
TCGA-AC-A8OQ
TCGA-BH-A0DH
TCGA-E9-A2JT
TCGA-A2-A04P
TCGA-E9-A1NH
TCGA-AN-A0XR
TCGA-A7-A26I
TCGA-A8-A08X
TCGA-E9-A5FK
TCGA-BH-A203
TCGA-OL-A66N
TCGA-AO-A0J4
TCGA-LL-A5YM
TCGA-LL-A73Y
TCGA-GM-A2DD
TCGA-AR-A0TP
TCGA-E2-A107
TCGA-BH-A18R
TCGA-A7-A0DB
TCGA-AO-A12B
TCGA-A2-A3XS
TCGA-AN-A0FW
TCGA-E2-A14T
TCGA-A7-A3J1
TCGA-S3-AA14
TCGA-BH-A0AY
TCGA-E2-A1LS
TCGA-D8-A1XM
TCGA-EW-A6SC
TCGA-AR-A1AK
TCGA-A2-A0CU
TCGA-BH-A0AU
TCGA-BH-A0DV
TCGA-C8-A135
TCGA-A8-A09D
TCGA-B6-A0IG
TCGA-D8-A1Y1
TCGA-A2-A0T5
TCGA-LD-A66U
TCGA-A2-A0CT
TCGA-D8-A27L
TCGA-BH-A0E1
TCGA-E2-A14X
TCGA-A8-A06N
TCGA-AO-A1KP
TCGA-EW-A1OX
TCGA-AR-A1AY
TCGA-AR-A0TV
TCGA-D8-A1J8
TCGA-EW-A1IY
TCGA-E9-A1RH
TCGA-E9-A1RH
TCGA-E2-A573
TCGA-BH-A1FC
TCGA-OL-A5RU
TCGA-AR-A1AI
TCGA-C8-A12Z
TCGA-B6-A2IU
TCGA-C8-A133
TCGA-LL-A5YO
TCGA-AR-A1AQ
TCGA-A7-A6VW
TCGA-AC-A3TN
TCGA-EW-A1P0
TCGA-A2-A0SW
TCGA-E9-A3HO
TCGA-A8-A09C
TCGA-AQ-A04J
TCGA-XX-A899
TCGA-BH-A1FR
TCGA-BH-A1FR
TCGA-BH-A0DS
TCGA-BH-A18F
TCGA-D8-A147
TCGA-A2-A0EU
TCGA-AC-A3EH
TCGA-E2-A14O
TCGA-A2-A0CP
TCGA-E9-A245
TCGA-BH-A0HO
TCGA-A2-A0D3
TCGA-E9-A2JS
TCGA-A8-A07E
TCGA-B6-A40B
TCGA-BH-A0E2
TCGA-E2-A150
TCGA-AO-A125
TCGA-A8-A0A6
TCGA-A7-A13H
TCGA-A8-A07J
TCGA-A2-A0EM
TCGA-AO-A12D
TCGA-D8-A73X
TCGA-A7-A0DC
TCGA-A7-A425
TCGA-AO-A0JA
TCGA-A8-A085
TCGA-C8-A12P
TCGA-A2-A0YF
TCGA-BH-A0W3
TCGA-OL-A66L
TCGA-D8-A142
TCGA-AR-A2LO
TCGA-A8-A0AD
TCGA-E2-A1AZ
TCGA-A2-A25A
TCGA-BH-A18M
TCGA-E2-A1IH
TCGA-D8-A73W
TCGA-B6-A0WW
TCGA-AC-A2FE
TCGA-BH-A0EB
TCGA-E2-A15F
TCGA-A7-A56D
TCGA-AN-A0AR
TCGA-AR-A1AT
TCGA-AO-A0JI
TCGA-E9-A3Q9
TCGA-E9-A5FL
TCGA-A2-A1G4
TCGA-E2-A106
TCGA-A8-A06R
TCGA-E9-A3QA
TCGA-GM-A2DA
TCGA-A2-A0YG
TCGA-EW-A1IZ
TCGA-EW-A1PD
TCGA-AN-A0XN
TCGA-AR-A0TX
TCGA-E2-A1L8
TCGA-A2-A4RX
TCGA-LL-A5YN
TCGA-E2-A2P6
TCGA-C8-A26W
TCGA-E9-A1N5
TCGA-E9-A1N5
TCGA-A8-A086
TCGA-S3-A6ZF
TCGA-BH-A0B6
TCGA-AC-A8OR
TCGA-S3-AA15
TCGA-AQ-A04H
TCGA-PL-A8LX
TCGA-BH-A0HY
TCGA-A2-A0YE
TCGA-BH-A0BC
TCGA-C8-A1HM
TCGA-A7-A4SD
TCGA-AN-A04D
TCGA-AO-A03R
TCGA-EW-A1PA
TCGA-AN-A0FK
TCGA-E2-A154
TCGA-AR-A2LK
TCGA-BH-A0H5
TCGA-A8-A07L
TCGA-EW-A1J6
TCGA-B6-A0X7
TCGA-BH-A0DH
TCGA-E2-A14P
TCGA-AC-A3YJ
TCGA-D8-A27M
TCGA-AR-A5QP
TCGA-AO-A12F
TCGA-EW-A1OV
TCGA-BH-A0H6
TCGA-D8-A1XJ
TCGA-A8-A09A
TCGA-A2-A1FX
TCGA-A2-A0YL
TCGA-C8-A1HE
TCGA-A2-A04T
TCGA-EW-A1PE
TCGA-E2-A1IL
TCGA-PE-A5DD
TCGA-BH-A203
TCGA-C8-A1HO
TCGA-D8-A1JL
TCGA-BH-A18M
TCGA-AO-A0JF
TCGA-A7-A0DB
TCGA-A7-A0DB
TCGA-E2-A15O
TCGA-GM-A5PX
TCGA-E9-A1ND
TCGA-E2-A152
TCGA-A7-A13D
TCGA-A7-A13D
TCGA-B6-A401
TCGA-D8-A1X8
TCGA-AC-A23C
TCGA-BH-A18H
TCGA-5L-AAT0
TCGA-BH-A202
TCGA-D8-A1XW
TCGA-EW-A2FR
TCGA-A8-A07C
TCGA-EW-A1PB
TCGA-AC-A2FM
TCGA-AC-A2FM
TCGA-E2-A105
TCGA-AR-A1AX
TCGA-EW-A1OW
TCGA-AC-A2FO
TCGA-BH-A0HQ
TCGA-A2-A04R
TCGA-BH-A1FJ
TCGA-AR-A1AM
TCGA-E2-A1LG
TCGA-E2-A14W
TCGA-EW-A2FV
TCGA-E2-A576
TCGA-A2-A0CM
TCGA-AC-A3OD
TCGA-D8-A1X5
TCGA-AR-A1AR
TCGA-E9-A1ND
TCGA-C8-A3M8
TCGA-AO-A1KO
TCGA-AN-A0FN
TCGA-B6-A0RH
TCGA-AO-A03P
TCGA-BH-A1FU
TCGA-BH-A1FU
TCGA-A1-A0SE
TCGA-BH-A0C1
TCGA-OL-A5D6
TCGA-D8-A27P
TCGA-AR-A0TR
TCGA-AC-A2QI
TCGA-BH-A0AW
TCGA-AQ-A1H2
TCGA-BH-A0EE
TCGA-A7-A6VY
TCGA-E9-A1RF
TCGA-AO-A129
TCGA-E9-A1R0
TCGA-EW-A1P1
TCGA-AR-A0TZ
TCGA-E2-A158
TCGA-GM-A2DL
TCGA-E2-A109
TCGA-C8-A27B
TCGA-E2-A2P5
TCGA-A7-A0DB
TCGA-A7-A0DC
TCGA-D8-A140
TCGA-A7-A13D
TCGA-AR-A5QQ
TCGA-OL-A5DA
TCGA-E9-A1R6
TCGA-BH-A18J
TCGA-LQ-A4E4
TCGA-C8-A27A
TCGA-E9-A1NE
TCGA-A2-A25D
TCGA-AR-A24X
TCGA-BH-A201
TCGA-D8-A145
TCGA-E9-A1N8
TCGA-C8-A26V
TCGA-B6-A1KF
TCGA-BH-A1FJ
TCGA-BH-A18R
TCGA-BH-A0BP
TCGA-BH-A0DK
TCGA-AN-A03Y
TCGA-EW-A1P6
TCGA-AC-A5EH
TCGA-A1-A0SD
TCGA-A2-A0SU
TCGA-E9-A1NI
TCGA-B6-A0RQ
TCGA-E9-A1RF
TCGA-A2-A0CX
TCGA-E2-A158
TCGA-A2-A25F
TCGA-AC-A23G
TCGA-E2-A15D
TCGA-OL-A5D7
 
 
 
 
  
 
 /tmp/ipykernel_1042853/4149498380.py:48: DeprecationWarning: the `interpolation=` argument to nanquantile was renamed to `method=`, which has additional options.
Users of the modes 'nearest', 'lower', 'higher', or 'midpoint' are encouraged to review the method they used. (Deprecated NumPy 1.22)
  nf  = conorm.tmm_norm_factors(genecount_df)
 
 
 
 
  
 
 TCGA-13-1489
TCGA-61-2101
TCGA-61-1900
TCGA-29-1784
TCGA-61-1725
TCGA-24-1470
TCGA-24-1546
TCGA-31-1956
TCGA-13-0913
TCGA-13-1477
TCGA-59-2363
TCGA-29-1701
TCGA-24-2267
TCGA-13-0884
TCGA-09-2054
TCGA-24-2035
TCGA-29-1711
TCGA-24-1923
TCGA-31-1959
TCGA-24-1103
TCGA-13-0765
TCGA-61-1911
TCGA-29-1783
TCGA-24-1424
TCGA-57-1582
TCGA-09-2051
TCGA-13-1510
TCGA-23-1114
TCGA-24-1553
TCGA-61-1914
TCGA-29-1781
TCGA-36-1570
TCGA-29-1763
TCGA-61-2000
TCGA-61-2104
TCGA-23-1119
TCGA-31-1944
TCGA-25-2398
TCGA-29-1710
TCGA-61-2097
TCGA-61-2113
TCGA-59-2355
TCGA-25-1315
TCGA-04-1356
TCGA-61-1907
TCGA-61-2003
TCGA-29-1762
TCGA-30-1861
TCGA-20-1682
TCGA-24-1928
TCGA-30-1860
TCGA-04-1651
TCGA-29-1776
TCGA-29-1688
TCGA-24-2297
TCGA-23-1109
TCGA-24-2293
TCGA-24-2289
TCGA-04-1361
TCGA-61-1738
TCGA-13-1409
TCGA-23-1023
TCGA-13-0886
TCGA-61-1721
TCGA-24-1471
TCGA-09-2056
TCGA-24-1563
TCGA-24-2262
TCGA-61-2092
TCGA-24-1924
TCGA-25-1628
TCGA-09-1669
TCGA-29-1761
TCGA-09-2045
TCGA-24-1423
TCGA-24-0979
TCGA-09-1668
TCGA-13-0720
TCGA-13-1403
TCGA-36-1571
TCGA-25-1318
TCGA-29-1785
TCGA-61-1741
TCGA-13-0714
TCGA-25-1633
TCGA-61-2012
TCGA-24-1567
TCGA-23-2084
TCGA-13-0730
TCGA-36-1576
TCGA-13-0725
TCGA-23-1029
TCGA-09-1666
TCGA-30-1862
TCGA-13-0883
TCGA-13-1501
TCGA-24-1616
TCGA-24-1930
TCGA-09-1670
TCGA-57-1994
TCGA-24-1552
TCGA-10-0938
TCGA-25-1328
TCGA-29-1768
TCGA-13-0920
TCGA-29-1707
TCGA-25-2042
TCGA-13-1408
TCGA-13-1505
TCGA-61-2111
TCGA-24-2254
TCGA-20-1687
TCGA-25-2393
TCGA-24-1843
TCGA-13-0724
TCGA-23-1110
TCGA-24-1845
TCGA-24-1560
TCGA-23-1026
TCGA-5X-AA5U
TCGA-24-1564
TCGA-61-1919
TCGA-24-1565
TCGA-31-1946
TCGA-29-1774
TCGA-04-1519
TCGA-24-2020
TCGA-23-2078
TCGA-04-1655
TCGA-24-2261
TCGA-09-1661
TCGA-23-2077
TCGA-36-1581
TCGA-13-0924
TCGA-24-2027
TCGA-04-1338
TCGA-61-1918
TCGA-61-1995
TCGA-24-2290
TCGA-29-2427
TCGA-04-1364
TCGA-24-1558
TCGA-61-2002
TCGA-13-1411
TCGA-25-2399
TCGA-09-2053
TCGA-25-1312
TCGA-24-1544
TCGA-29-1766
TCGA-09-1667
TCGA-04-1365
TCGA-30-1866
TCGA-29-2428
TCGA-24-2038
TCGA-24-1417
TCGA-24-0982
TCGA-23-1030
TCGA-29-A5NZ
TCGA-24-1550
TCGA-23-1809
TCGA-04-1332
TCGA-13-1499
TCGA-25-2391
TCGA-24-2280
TCGA-23-1123
TCGA-04-1530
TCGA-09-1665
TCGA-24-1425
TCGA-23-1122
TCGA-24-1844
TCGA-61-2008
TCGA-61-2008
TCGA-25-1317
TCGA-13-A5FT
TCGA-25-1316
TCGA-24-0970
TCGA-13-1492
TCGA-25-1323
TCGA-57-1585
TCGA-24-1551
TCGA-10-0931
TCGA-36-1569
TCGA-13-0800
TCGA-13-1497
TCGA-OY-A56Q
TCGA-29-1777
TCGA-57-1583
TCGA-36-1568
TCGA-25-1623
TCGA-24-2271
TCGA-61-1724
TCGA-13-1405
TCGA-61-1728
TCGA-24-1427
TCGA-61-1733
TCGA-24-1422
TCGA-25-1630
TCGA-13-0901
TCGA-24-2288
TCGA-13-0905
TCGA-10-0928
TCGA-24-1430
TCGA-13-0885
TCGA-24-1847
TCGA-24-2023
TCGA-24-2281
TCGA-24-1434
TCGA-13-0762
TCGA-24-1604
TCGA-24-0968
TCGA-23-1113
TCGA-25-1626
TCGA-09-0364
TCGA-24-1846
TCGA-25-1631
TCGA-25-1321
TCGA-13-1511
TCGA-29-1690
TCGA-13-0888
TCGA-04-1331
TCGA-61-1910
TCGA-13-0897
TCGA-13-0804
TCGA-24-2298
TCGA-13-0727
TCGA-61-2110
TCGA-29-1696
TCGA-23-1024
TCGA-04-1341
TCGA-13-0900
TCGA-20-1683
TCGA-25-1322
TCGA-10-0937
TCGA-61-2009
TCGA-04-1343
TCGA-13-0795
TCGA-29-1703
TCGA-24-2036
TCGA-13-1487
TCGA-25-1627
TCGA-13-0906
TCGA-13-1496
TCGA-36-1577
TCGA-57-1584
TCGA-29-1705
TCGA-13-1407
TCGA-23-1120
TCGA-04-1357
TCGA-13-0893
TCGA-24-1435
TCGA-04-1536
TCGA-04-1514
TCGA-61-1737
TCGA-57-1993
TCGA-61-2098
TCGA-25-1635
TCGA-20-1686
TCGA-25-1326
TCGA-57-1586
TCGA-24-1842
TCGA-23-1027
TCGA-25-1634
TCGA-25-2392
TCGA-59-2348
TCGA-30-1718
TCGA-30-1857
TCGA-24-1469
TCGA-36-1580
TCGA-24-1474
TCGA-24-1426
TCGA-13-0768
TCGA-13-1498
TCGA-23-1022
TCGA-29-1691
TCGA-23-1107
TCGA-24-2026
TCGA-23-1021
TCGA-24-1105
TCGA-30-1891
TCGA-29-1693
TCGA-25-2404
TCGA-13-1509
TCGA-04-1362
TCGA-25-1632
TCGA-24-1562
TCGA-30-1853
TCGA-20-0987
TCGA-25-1319
TCGA-36-1574
TCGA-24-1557
TCGA-04-1542
TCGA-23-1118
TCGA-25-1877
TCGA-13-1483
TCGA-29-1770
TCGA-59-2350
TCGA-04-1648
TCGA-24-2024
TCGA-13-0891
TCGA-13-0797
TCGA-24-1418
TCGA-13-0887
TCGA-13-2060
TCGA-09-0369
TCGA-09-2048
TCGA-29-1694
TCGA-61-2088
TCGA-13-0726
TCGA-29-1695
TCGA-24-1467
TCGA-24-1549
TCGA-25-2401
TCGA-20-0991
TCGA-13-0923
TCGA-24-1413
TCGA-24-1464
TCGA-25-2397
TCGA-29-1778
TCGA-31-1950
TCGA-13-1485
TCGA-13-1507
TCGA-24-1419
TCGA-61-1998
TCGA-10-0936
TCGA-23-1116
TCGA-61-2102
TCGA-59-2352
TCGA-13-1512
TCGA-09-1662
TCGA-24-2033
TCGA-10-0927
TCGA-13-0911
TCGA-10-0933
TCGA-09-0367
TCGA-25-1329
TCGA-13-1488
TCGA-24-1428
TCGA-13-1410
TCGA-59-2354
TCGA-61-2109
TCGA-23-1028
TCGA-24-1104
TCGA-WR-A838
TCGA-24-0966
TCGA-29-1769
TCGA-09-2044
TCGA-13-1495
TCGA-04-1350
TCGA-09-1673
TCGA-13-0766
TCGA-29-2425
TCGA-09-1659
TCGA-24-1603
TCGA-09-0366
TCGA-30-1892
TCGA-61-1736
TCGA-24-1431
TCGA-04-1347
TCGA-23-1111
TCGA-13-1404
TCGA-59-A5PD
TCGA-25-2400
TCGA-VG-A8LO
TCGA-13-1489
TCGA-25-1313
TCGA-13-0916
TCGA-13-0908
TCGA-31-1953
TCGA-59-2351
TCGA-31-1951
TCGA-24-1850
TCGA-13-1506
TCGA-29-2414
TCGA-29-2414
TCGA-25-1320
TCGA-30-1714
TCGA-25-1870
TCGA-24-1416
TCGA-29-1697
TCGA-25-2409
TCGA-25-2396
 
 
 
 
  
 
 /tmp/ipykernel_1042853/4149498380.py:48: DeprecationWarning: the `interpolation=` argument to nanquantile was renamed to `method=`, which has additional options.
Users of the modes 'nearest', 'lower', 'higher', or 'midpoint' are encouraged to review the method they used. (Deprecated NumPy 1.22)
  nf  = conorm.tmm_norm_factors(genecount_df)
 
 
 
 
  
 
 TCGA-PG-A5BC
TCGA-EY-A1GP
TCGA-AJ-A3BG
TCGA-BG-A0W1
TCGA-BS-A0U9
TCGA-A5-A0GB
TCGA-AX-A2HH
TCGA-BG-A0MS
TCGA-FL-A1YH
TCGA-BK-A4ZD
TCGA-AJ-A3NH
TCGA-QS-A5YQ
TCGA-EY-A2OP
TCGA-AX-A1CN
TCGA-EO-A3AU
TCGA-PG-A917
TCGA-BK-A139
TCGA-BS-A0TE
TCGA-EY-A1GD
TCGA-B5-A11I
TCGA-AJ-A6NU
TCGA-PG-A916
TCGA-D1-A1NX
TCGA-B5-A1MU
TCGA-B5-A0K9
TCGA-AJ-A3TW
TCGA-AJ-A3OL
TCGA-AP-A054
TCGA-A5-A0R8
TCGA-BS-A0UF
TCGA-D1-A3JQ
TCGA-AX-A1C9
TCGA-BK-A0CC
TCGA-A5-A0GN
TCGA-BK-A6W3
TCGA-EY-A3QX
TCGA-D1-A16B
TCGA-BS-A0UJ
TCGA-EO-A22S
TCGA-B5-A11N
TCGA-D1-A17L
TCGA-D1-A165
TCGA-A5-A0GQ
TCGA-D1-A2G5
TCGA-AP-A1DM
TCGA-AP-A0LH
TCGA-EY-A1GF
TCGA-AJ-A23M
TCGA-BK-A13C
TCGA-D1-A15Z
TCGA-EY-A547
TCGA-EO-A22T
TCGA-BG-A220
TCGA-EY-A2OQ
TCGA-AX-A2H8
TCGA-B5-A0K6
TCGA-D1-A15X
TCGA-AX-A2HG
TCGA-FI-A2D2
TCGA-AP-A053
TCGA-AJ-A3NE
TCGA-B5-A3FA
TCGA-AJ-A3NE
TCGA-D1-A3DG
TCGA-EY-A1GK
TCGA-BS-A0U7
TCGA-H5-A2HR
TCGA-A5-A0GI
TCGA-AP-A0LE
TCGA-FL-A1YG
TCGA-BK-A0CB
TCGA-AX-A063
TCGA-AJ-A3BD
TCGA-D1-A160
TCGA-FI-A2CX
TCGA-PG-A7D5
TCGA-KP-A3VZ
TCGA-D1-A17D
TCGA-D1-A16O
TCGA-AX-A0IS
TCGA-A5-A1OK
TCGA-EC-A1NJ
TCGA-D1-A17C
TCGA-E6-A1LX
TCGA-AJ-A5DV
TCGA-FI-A2F8
TCGA-D1-A17B
TCGA-AJ-A3QS
TCGA-BG-A3EW
TCGA-A5-A0G9
TCGA-AP-A05N
TCGA-D1-A16Q
TCGA-E6-A1M0
TCGA-E6-A1M0
TCGA-AJ-A3NG
TCGA-BS-A0UM
TCGA-AX-A1CF
TCGA-BK-A139
TCGA-AX-A0J0
TCGA-AX-A2H7
TCGA-AX-A06F
TCGA-B5-A3FB
TCGA-AJ-A3IA
TCGA-DF-A2KS
TCGA-AX-A06B
TCGA-BK-A4ZD
TCGA-D1-A16J
TCGA-SL-A6JA
TCGA-JU-AAVI
TCGA-BG-A0VT
TCGA-AJ-A3NH
TCGA-QS-A744
TCGA-A5-A0GJ
TCGA-AP-A0LJ
TCGA-BS-A0U8
TCGA-AP-A1E4
TCGA-D1-A2G0
TCGA-BG-A0YV
TCGA-AP-A056
TCGA-AJ-A3BI
TCGA-EY-A1GR
TCGA-BG-A3EW
TCGA-BG-A0M7
TCGA-D1-A0ZN
TCGA-EY-A3L3
TCGA-BG-A0MQ
TCGA-AX-A1CF
TCGA-PG-A6IB
TCGA-AJ-A2QK
TCGA-D1-A162
TCGA-BK-A139
TCGA-BG-A0M3
TCGA-AJ-A3NF
TCGA-EY-A1G8
TCGA-AX-A0J0
TCGA-A5-A0VO
TCGA-AX-A3FV
TCGA-AX-A05W
TCGA-KJ-A3U4
TCGA-B5-A11O
TCGA-FI-A3PX
TCGA-B5-A0K2
TCGA-AX-A2HC
TCGA-BG-A187
TCGA-AW-A1PO
TCGA-B5-A11R
TCGA-BG-A222
TCGA-KP-A3W0
TCGA-B5-A11J
TCGA-EY-A1GT
TCGA-BG-A0YU
TCGA-EY-A1GL
TCGA-B5-A1MY
TCGA-BK-A0CC
TCGA-AX-A2HD
TCGA-BS-A0TD
TCGA-AP-A0LV
TCGA-D1-A0ZP
TCGA-AJ-A3BK
TCGA-AP-A059
TCGA-EY-A1GQ
TCGA-EC-A24G
TCGA-BG-A221
TCGA-4E-A92E
TCGA-BG-A0M4
TCGA-BK-A6W4
TCGA-BS-A0TI
TCGA-BS-A0T9
TCGA-AJ-A3EM
TCGA-AX-A2IO
TCGA-AP-A0LL
TCGA-AX-A3G9
TCGA-B5-A0JS
TCGA-AX-A05S
TCGA-AX-A1C4
TCGA-D1-A0ZO
TCGA-A5-A1OG
TCGA-BG-A0MC
TCGA-D1-A1NY
TCGA-B5-A0JT
TCGA-AP-A0LD
TCGA-AP-A1E0
TCGA-AJ-A3BH
TCGA-AP-A05O
TCGA-A5-A0R7
TCGA-A5-A2K7
TCGA-EO-A22R
TCGA-B5-A1N2
TCGA-AX-A2H8
TCGA-D1-A169
TCGA-FL-A1YI
TCGA-AP-A0L8
TCGA-D1-A1NS
TCGA-EY-A5W2
TCGA-EO-A3AZ
TCGA-AP-A0LO
TCGA-D1-A15V
TCGA-A5-A2K3
TCGA-AX-A2H2
TCGA-BG-A2AD
TCGA-B5-A0K4
TCGA-D1-A17A
TCGA-D1-A174
TCGA-AP-A0LN
TCGA-D1-A1O5
TCGA-D1-A177
TCGA-B5-A3FC
TCGA-BG-A0MG
TCGA-A5-A0VQ
TCGA-D1-A17H
TCGA-AX-A1CE
TCGA-EY-A1GS
TCGA-EO-A1Y7
TCGA-FL-A1YT
TCGA-D1-A0ZZ
TCGA-E6-A2P9
TCGA-AP-A0LS
TCGA-DI-A1C3
TCGA-FL-A1YQ
TCGA-D1-A0ZS
TCGA-B5-A11E
TCGA-BG-A0M9
TCGA-QS-A5YR
TCGA-BS-A0V6
TCGA-AX-A2HF
TCGA-B5-A0K0
TCGA-B5-A5OC
TCGA-FI-A2D0
TCGA-AJ-A8CT
TCGA-DI-A2QT
TCGA-BG-A0MH
TCGA-QF-A5YT
TCGA-A5-A3LP
TCGA-B5-A11F
TCGA-PG-A915
TCGA-AX-A1C5
TCGA-B5-A0JX
TCGA-D1-A15W
TCGA-KP-A3W1
TCGA-AX-A1CA
TCGA-EY-A1GH
TCGA-EY-A1GW
TCGA-AP-A3K1
TCGA-D1-A103
TCGA-EO-A3AV
TCGA-AX-A2HD
TCGA-D1-A3DA
TCGA-EO-A3B1
TCGA-B5-A11Y
TCGA-AJ-A2QN
TCGA-BG-A18B
TCGA-A5-A0GM
TCGA-AJ-A2QL
TCGA-BS-A0U5
TCGA-AJ-A2QL
TCGA-BS-A0TC
TCGA-EY-A214
TCGA-AX-A05U
TCGA-B5-A11W
TCGA-EY-A1GI
TCGA-D1-A161
TCGA-EO-A3B0
TCGA-A5-A0R6
TCGA-BS-A0UA
TCGA-AP-A0LT
TCGA-A5-A0G5
TCGA-AX-A3G7
TCGA-BS-A0UT
TCGA-EY-A1GV
TCGA-BG-A0LW
TCGA-BG-A3PP
TCGA-AX-A2HK
TCGA-FL-A1YL
TCGA-A5-A0GA
TCGA-EO-A3AS
TCGA-EO-A3KX
TCGA-BS-A0WQ
TCGA-AX-A3G8
TCGA-AX-A1CR
TCGA-QS-A8F1
TCGA-BK-A0CA
TCGA-BK-A0CA
TCGA-KP-A3W4
TCGA-BG-A0MO
TCGA-A5-A0GX
TCGA-EY-A54A
TCGA-AP-A1E3
TCGA-DI-A2QU
TCGA-B5-A3FD
TCGA-B5-A1MV
TCGA-B5-A0JY
TCGA-EY-A1GU
TCGA-EY-A1GM
TCGA-AX-A2HJ
TCGA-AJ-A23O
TCGA-D1-A0ZV
TCGA-DI-A2QY
TCGA-DI-A2QY
TCGA-FL-A1YN
TCGA-AX-A05T
TCGA-BK-A56F
TCGA-BG-A18A
TCGA-BK-A0C9
TCGA-AX-A2HC
TCGA-D1-A16I
TCGA-BG-A0MA
TCGA-FI-A2EY
TCGA-A5-A0GR
TCGA-A5-A0GP
TCGA-D1-A16D
TCGA-BK-A13B
TCGA-DI-A1BY
TCGA-AP-A1E1
TCGA-B5-A0JN
TCGA-B5-A11U
TCGA-AX-A1C7
TCGA-EY-A215
TCGA-AP-A05J
TCGA-AX-A060
TCGA-EO-A22U
TCGA-BK-A0CC
TCGA-EY-A549
TCGA-AX-A1CP
TCGA-B5-A121
TCGA-FI-A2EU
TCGA-B5-A11S
TCGA-BG-A2AE
TCGA-D1-A3JP
TCGA-A5-A1OH
TCGA-D1-A179
TCGA-A5-A0GV
TCGA-FI-A2D4
TCGA-FI-A2EW
TCGA-BK-A26L
TCGA-AJ-A3EJ
TCGA-EY-A72D
TCGA-A5-A3LO
TCGA-AX-A3FZ
TCGA-AJ-A3I9
TCGA-EY-A1H0
TCGA-SL-A6J9
TCGA-AP-A0LI
TCGA-B5-A11M
TCGA-AP-A05P
TCGA-BS-A0VI
TCGA-BS-A0TA
TCGA-AP-A1DR
TCGA-A5-A0RA
TCGA-AP-A0L9
TCGA-AP-A05A
TCGA-AX-A0IZ
TCGA-D1-A16N
TCGA-BG-A0M8
TCGA-A5-A0GW
TCGA-BG-A0M0
TCGA-B5-A1MZ
TCGA-BG-A0VX
TCGA-AX-A05Y
TCGA-AP-A1DP
TCGA-D1-A3DH
TCGA-A5-A7WK
TCGA-E6-A1LZ
TCGA-EY-A1GE
TCGA-D1-A17K
TCGA-2E-A9G8
TCGA-BG-A2AD
TCGA-BG-A0MT
TCGA-A5-A0G2
TCGA-D1-A0ZQ
TCGA-D1-A1NU
TCGA-B5-A11L
TCGA-DI-A2QU
TCGA-AX-A05Z
TCGA-AX-A1CI
TCGA-BG-A0MU
TCGA-BG-A0LX
TCGA-A5-A0G3
TCGA-AP-A1DO
TCGA-EO-A3KW
TCGA-AX-A3G4
TCGA-BG-A2L7
TCGA-QF-A5YS
TCGA-DI-A1NN
TCGA-AX-A3GI
TCGA-AJ-A3EK
TCGA-EY-A2OO
TCGA-A5-A0GE
TCGA-D1-A17M
TCGA-AX-A1CJ
TCGA-BS-A0UL
TCGA-BG-A0RY
TCGA-D1-A2G6
TCGA-AJ-A8CW
TCGA-B5-A11Z
TCGA-AP-A052
TCGA-A5-A0GH
TCGA-D1-A1O0
TCGA-AP-A051
TCGA-B5-A11P
TCGA-A5-A0GG
TCGA-A5-A0G1
TCGA-FL-A1YV
TCGA-5S-A9Q8
TCGA-BG-A0M6
TCGA-DF-A2KU
TCGA-EY-A4KR
TCGA-B5-A11Q
TCGA-DF-A2KN
TCGA-B5-A0K7
TCGA-BK-A26L
TCGA-BG-A0VV
TCGA-FI-A2F4
TCGA-SJ-A6ZI
TCGA-AX-A1C8
TCGA-AX-A064
TCGA-AX-A06H
TCGA-D1-A0ZU
TCGA-BK-A13C
TCGA-B5-A0JV
TCGA-B5-A11G
TCGA-EO-A3AY
TCGA-AP-A5FX
TCGA-B5-A1MW
TCGA-DF-A2KR
TCGA-KP-A3W3
TCGA-DF-A2KZ
TCGA-B5-A3F9
TCGA-EY-A1G7
TCGA-A5-A7WJ
TCGA-A5-A1OF
TCGA-DF-A2KY
TCGA-AX-A05Y
TCGA-BG-A3PP
TCGA-AX-A3GB
TCGA-D1-A102
TCGA-FL-A3WE
TCGA-AX-A0IW
TCGA-D1-A16E
TCGA-D1-A0ZR
TCGA-AX-A2IN
TCGA-AP-A1DH
TCGA-B5-A11H
TCGA-A5-A0R9
TCGA-EY-A1GO
TCGA-A5-A0VP
TCGA-BK-A0CB
TCGA-B5-A0JU
TCGA-AJ-A8CV
TCGA-AX-A06L
TCGA-B5-A0KB
TCGA-EO-A2CG
TCGA-FL-A1YU
TCGA-B5-A0K3
TCGA-B5-A0K1
TCGA-BG-A186
TCGA-FL-A1YM
TCGA-AJ-A3BF
TCGA-AX-A1CI
TCGA-AX-A06D
TCGA-AX-A0J1
TCGA-D1-A16R
TCGA-EO-A3L0
TCGA-BS-A0UV
TCGA-B5-A11V
TCGA-D1-A1O8
TCGA-AP-A1DK
TCGA-D1-A17T
TCGA-EO-A22Y
TCGA-FI-A3PV
TCGA-AP-A0LP
TCGA-AX-A3FX
TCGA-AP-A05D
TCGA-AX-A3FW
TCGA-D1-A17R
TCGA-DI-A1BU
TCGA-EO-A2CH
TCGA-D1-A17N
TCGA-BG-A0MK
TCGA-BG-A0M2
TCGA-D1-A1NW
TCGA-AX-A3FS
TCGA-A5-AB3J
TCGA-AJ-A3OK
TCGA-EY-A1GX
TCGA-AJ-A5DW
TCGA-EY-A2OM
TCGA-SJ-A6ZJ
TCGA-AX-A2H4
TCGA-D1-A163
TCGA-EO-A1Y5
TCGA-BK-A26L
TCGA-FL-A1YF
TCGA-B5-A0K8
TCGA-A5-A2K4
TCGA-BG-A18C
TCGA-EY-A210
TCGA-PG-A914
TCGA-D1-A101
TCGA-B5-A1MX
TCGA-AJ-A2QO
TCGA-BS-A0TJ
TCGA-AX-A3G1
TCGA-AX-A2H5
TCGA-FI-A2F9
TCGA-AX-A06J
TCGA-AP-A1DQ
TCGA-AJ-A23N
TCGA-DF-A2L0
TCGA-D1-A167
TCGA-AX-A1CC
TCGA-BG-A0VW
TCGA-AX-A0IZ
TCGA-D1-A2G7
TCGA-A5-A0GD
TCGA-D1-A1NZ
TCGA-K6-A3WQ
TCGA-EO-A1Y8
TCGA-AJ-A3OJ
TCGA-AX-A3G3
TCGA-AX-A3G6
TCGA-D1-A17S
TCGA-EC-A1QX
TCGA-AP-A0LF
TCGA-AX-A3FT
TCGA-AP-A05H
TCGA-B5-A5OD
TCGA-D1-A16X
TCGA-EO-A3KU
TCGA-EY-A212
TCGA-AX-A2HA
TCGA-A5-A2K2
TCGA-AX-A2HA
TCGA-B5-A1MR
TCGA-DI-A0WH
TCGA-BG-A0VZ
TCGA-A5-A1OJ
TCGA-EY-A2ON
TCGA-BK-A0CA
TCGA-FI-A2D5
TCGA-BS-A0V4
TCGA-BS-A0V8
TCGA-BS-A0V7
TCGA-D1-A17Q
TCGA-D1-A17F
TCGA-B5-A0JR
TCGA-AX-A1CK
TCGA-A5-A0GU
TCGA-BS-A0TG
TCGA-AX-A062
TCGA-D1-A1O7
TCGA-EY-A548
TCGA-BG-A0W2
TCGA-E6-A8L9
TCGA-B5-A11X
TCGA-AJ-A3NC
TCGA-D1-A16G
TCGA-FI-A2D6
TCGA-5B-A90C
TCGA-AP-A0LG
TCGA-AX-A1CK
TCGA-AP-A1DV
TCGA-FI-A2CY
TCGA-D1-A16Y
TCGA-AJ-A3NC
TCGA-B5-A3FH
TCGA-AJ-A3EL
TCGA-D1-A168
TCGA-E6-A2P8
TCGA-AX-A0IU
TCGA-EY-A1GC
TCGA-D1-A17U
TCGA-AP-A0LM
TCGA-B5-A3S1
TCGA-B5-A1MS
TCGA-D1-A175
TCGA-DI-A1NO
TCGA-BG-A0MI
TCGA-AJ-A2QM
TCGA-D1-A16F
TCGA-A5-A2K5
TCGA-EO-A22X
TCGA-DF-A2KV
TCGA-FI-A2EX
TCGA-D1-A176
TCGA-B5-A0JZ
TCGA-D1-A16V
TCGA-B5-A5OE
TCGA-D1-A16S
TCGA-BK-A139
 
 
 
 
  
 
 /tmp/ipykernel_1042853/4149498380.py:48: DeprecationWarning: the `interpolation=` argument to nanquantile was renamed to `method=`, which has additional options.
Users of the modes 'nearest', 'lower', 'higher', or 'midpoint' are encouraged to review the method they used. (Deprecated NumPy 1.22)
  nf  = conorm.tmm_norm_factors(genecount_df)
 
 
 
 
  
 
 TCGA-C5-A1BK
TCGA-EA-A5ZF
TCGA-BI-A20A
TCGA-IR-A3LA
TCGA-C5-A2LV
TCGA-VS-A9V2
TCGA-C5-A1M5
TCGA-Q1-A6DT
TCGA-VS-A8EG
TCGA-JW-A5VH
TCGA-HM-A6W2
TCGA-HM-A6W2
TCGA-IR-A3LL
TCGA-C5-A1MQ
TCGA-DG-A2KK
TCGA-C5-A1ML
TCGA-C5-A1BL
TCGA-VS-A8EH
TCGA-EK-A2PK
TCGA-C5-A3HF
TCGA-EK-A2PM
TCGA-C5-A1MP
TCGA-C5-A3HL
TCGA-ZJ-AAXF
TCGA-JW-A5VG
TCGA-VS-A94Y
TCGA-UC-A7PD
TCGA-C5-A1M8
TCGA-C5-A3HD
TCGA-FU-A770
TCGA-LP-A5U3
TCGA-ZJ-AAX4
TCGA-UC-A7PF
TCGA-C5-A0TN
TCGA-EA-A5O9
TCGA-VS-AA62
TCGA-C5-A8XH
TCGA-C5-A2LS
TCGA-VS-A8QA
TCGA-JW-AAVH
TCGA-C5-A7CK
TCGA-VS-A9V3
TCGA-MU-A8JM
TCGA-EX-A8YF
TCGA-EX-A69M
TCGA-LP-A5U2
TCGA-EX-A1H6
TCGA-JX-A3Q0
TCGA-VS-A8QF
TCGA-C5-A1MJ
TCGA-VS-A94X
TCGA-C5-A1BF
TCGA-VS-A8QM
TCGA-C5-A1MN
TCGA-DS-A1OB
TCGA-C5-A1BE
TCGA-VS-A9UQ
TCGA-VS-A8EB
TCGA-EA-A556
TCGA-DG-A2KL
TCGA-VS-A8EJ
TCGA-MA-AA3Y
TCGA-VS-A94W
TCGA-C5-A1MH
TCGA-LP-A4AX
TCGA-EA-A43B
TCGA-C5-A8ZZ
TCGA-JX-A3Q8
TCGA-EA-A5ZD
TCGA-DR-A0ZL
TCGA-BI-A0VS
TCGA-BI-A0VR
TCGA-Q1-A73P
TCGA-EA-A3Y4
TCGA-EA-A3HT
TCGA-VS-A950
TCGA-JX-A3PZ
TCGA-EA-A439
TCGA-ZJ-AAXI
TCGA-EK-A2PL
TCGA-DS-A1OA
TCGA-ZX-AA5X
TCGA-VS-A9UU
TCGA-WL-A834
TCGA-VS-A9UO
TCGA-C5-A7X3
TCGA-MY-A5BF
TCGA-MY-A5BF
TCGA-C5-A1MF
TCGA-EK-A2PG
TCGA-UC-A7PG
TCGA-UC-A7PG
TCGA-4J-AA1J
TCGA-JW-A5VI
TCGA-PN-A8MA
TCGA-C5-A7X8
TCGA-VS-A8Q9
TCGA-IR-A3LC
TCGA-ZJ-AAXT
TCGA-2W-A8YY
TCGA-C5-A7UC
TCGA-Q1-A73Q
TCGA-Q1-A6DW
TCGA-EA-A5ZE
TCGA-MU-A5YI
TCGA-DS-A0VK
TCGA-ZJ-AAXN
TCGA-VS-A9UD
TCGA-EK-A2RL
TCGA-DG-A2KH
TCGA-EA-A3HR
TCGA-DS-A3LQ
TCGA-FU-A40J
TCGA-IR-A3L7
TCGA-GH-A9DA
TCGA-FU-A3HY
TCGA-VS-A9UZ
TCGA-FU-A3WB
TCGA-EK-A2H1
TCGA-VS-A9UH
TCGA-VS-A94Z
TCGA-EA-A50E
TCGA-ZJ-AAXJ
TCGA-EX-A1H5
TCGA-HM-A4S6
TCGA-EA-A3HQ
TCGA-VS-A8Q8
TCGA-VS-A9U7
TCGA-EX-A69L
TCGA-FU-A3YQ
TCGA-EA-A1QS
TCGA-FU-A3HZ
TCGA-LP-A7HU
TCGA-FU-A3TQ
TCGA-Q1-A5R3
TCGA-DR-A0ZM
TCGA-LP-A4AW
TCGA-EA-A3HS
TCGA-IR-A3LI
TCGA-EK-A2IP
TCGA-VS-A9V4
TCGA-C5-A2M2
TCGA-MU-A51Y
TCGA-IR-A3LF
TCGA-VS-A9UJ
TCGA-C5-A1BJ
TCGA-EA-A78R
TCGA-VS-A9UL
TCGA-VS-A8EI
TCGA-EA-A410
TCGA-HM-A3JK
TCGA-DG-A2KJ
TCGA-JW-A852
TCGA-C5-A1BM
TCGA-MA-AA41
TCGA-C5-A1M6
TCGA-C5-A7UH
TCGA-EA-A411
TCGA-FU-A23K
TCGA-FU-A23L
TCGA-EA-A97N
TCGA-ZJ-AAXB
TCGA-C5-A2M1
TCGA-C5-A1BN
TCGA-C5-A8XK
TCGA-C5-A1BI
TCGA-VS-A9U5
TCGA-C5-A1MI
TCGA-C5-A8XI
TCGA-FU-A57G
TCGA-EA-A1QT
TCGA-VS-A958
TCGA-VS-A9U6
TCGA-EA-A3HU
TCGA-EK-A2RE
TCGA-VS-A954
TCGA-VS-A9UI
TCGA-FU-A5XV
TCGA-EA-A3QD
TCGA-EA-A4BA
TCGA-VS-A952
TCGA-EK-A2IR
TCGA-C5-A1ME
TCGA-VS-A9UY
TCGA-EA-A6QX
TCGA-C5-A1M9
TCGA-EA-A3QE
TCGA-ZJ-AB0I
TCGA-VS-A9V0
TCGA-VS-A9V1
TCGA-VS-A9UT
TCGA-EK-A2RC
TCGA-EK-A2RK
TCGA-JX-A5QV
TCGA-EK-A2RA
TCGA-MA-AA3X
TCGA-VS-A9UR
TCGA-C5-A7X5
TCGA-ZJ-AAXD
TCGA-VS-A8EC
TCGA-Q1-A73S
TCGA-ZJ-A8QQ
TCGA-DS-A5RQ
TCGA-ZJ-AAX8
TCGA-C5-A1MK
TCGA-FU-A3TX
TCGA-IR-A3LK
TCGA-FU-A3EO
TCGA-FU-A3EO
TCGA-C5-A2LX
TCGA-DS-A1O9
TCGA-C5-A7CL
TCGA-JW-A5VK
TCGA-MA-AA3Z
TCGA-C5-A3HE
TCGA-RA-A741
TCGA-C5-A8YR
TCGA-C5-A2LZ
TCGA-EK-A2PI
TCGA-EK-A2RM
TCGA-DS-A7WI
TCGA-VS-A8QH
TCGA-UC-A7PI
TCGA-Q1-A6DV
TCGA-EK-A2H0
TCGA-C5-A907
TCGA-EK-A2RO
TCGA-C5-A905
TCGA-VS-A9UV
TCGA-MY-A913
TCGA-VS-A9UB
TCGA-VS-A957
TCGA-VS-A8QC
TCGA-EK-A3GM
TCGA-VS-A9UC
TCGA-EK-A2RJ
TCGA-C5-A7UI
TCGA-C5-A8YT
TCGA-MY-A5BD
TCGA-EA-A44S
TCGA-DS-A7WF
TCGA-EK-A3GK
TCGA-Q1-A73O
TCGA-JW-A5VL
TCGA-C5-A7CM
TCGA-VS-A9V5
TCGA-EK-A2R8
TCGA-FU-A2QG
TCGA-C5-A7CH
TCGA-EK-A2R7
TCGA-EK-A2GZ
TCGA-Q1-A73R
TCGA-VS-A8EK
TCGA-C5-A7UE
TCGA-MA-AA42
TCGA-VS-A953
TCGA-FU-A3NI
TCGA-DS-A7WH
TCGA-ZJ-A8QR
TCGA-ZJ-AAXU
TCGA-C5-A8YQ
TCGA-EK-A3GJ
TCGA-C5-A7XC
TCGA-DS-A0VL
TCGA-VS-A8EL
TCGA-LP-A4AU
TCGA-HG-A2PA
TCGA-DS-A1OC
TCGA-C5-A2LY
TCGA-C5-A1BQ
TCGA-IR-A3LB
TCGA-C5-A8XJ
TCGA-ZJ-AAXA
TCGA-C5-A1M7
TCGA-C5-A901
TCGA-EX-A449
TCGA-ZJ-A8QO
TCGA-DS-A1OD
TCGA-EA-A5FO
TCGA-DS-A0VM
TCGA-MA-AA43
TCGA-VS-A9UM
TCGA-JW-A5VJ
TCGA-VS-A9UP
TCGA-ZJ-AB0H
TCGA-HM-A3JJ
TCGA-HM-A3JJ
TCGA-LP-A4AV
TCGA-IR-A3LH
TCGA-VS-A959
TCGA-C5-A7CG
TCGA-Q1-A5R2
TCGA-EX-A3L1
TCGA-EK-A3GN
TCGA-MY-A5BE
TCGA-MA-AA3W
TCGA-C5-A7CJ
TCGA-EK-A2RB
TCGA-R2-A69V
TCGA-DG-A2KM
TCGA-EK-A2RN
TCGA-EK-A2R9
TCGA-C5-A902
TCGA-Q1-A5R1
TCGA-JW-A69B
TCGA-C5-A2LT
TCGA-DS-A0VN
TCGA-C5-A7CO
TCGA-XS-A8TJ
 
 
 
 
  
 
 /tmp/ipykernel_1042853/4149498380.py:48: DeprecationWarning: the `interpolation=` argument to nanquantile was renamed to `method=`, which has additional options.
Users of the modes 'nearest', 'lower', 'higher', or 'midpoint' are encouraged to review the method they used. (Deprecated NumPy 1.22)
  nf  = conorm.tmm_norm_factors(genecount_df)
 
 
 
 
 
  
 
 
 
 
 In [ ]: 
 
 
     # ov_gdc_tmm = genecount_to_tmm_for_gsea(ov_gdc_genecount) 
 # ov_gdc_tmm.iloc[:,1:].to_csv("ov_gdc_tmm.csv") 
 # ov_gdc_tpm.to_csv("ov_gdc_tpm.csv") 
  
 
 
 
 
  
 
 
 
 
 In [5]: 
 
 
     f   =   open  (  'h.all.v2023.1.Hs.json'  ,   'r'  ) 
 h_all_json   =   json  .  loads  (  f  .  read  ()) 
 h_all_json 
 hallmark_gs   =   {  key  :   h_all_json  [  key  ][  "geneSymbols"  ]  for   key   in   h_all_json  .  keys  ()} 
 f   =   open  (  'c6.all.v2023.1.Hs.json'  ,   'r'  ) 
 c6_all_json   =   json  .  loads  (  f  .  read  ()) 
 c6_all_json 
 c6_gs   =   {  key  :   c6_all_json  [  key  ][  "geneSymbols"  ]  for   key   in   c6_all_json  .  keys  ()} 
 f   =   open  (  'c3.tft.gtrd.v2023.1.Hs.json'  ,   'r'  ) 
 c3_tft_json   =   json  .  loads  (  f  .  read  ()) 
 c3_tft_json 
 c3_gs   =   {  key  :   c3_tft_json  [  key  ][  "geneSymbols"  ]  for   key   in   c3_tft_json  .  keys  ()} 
  
 
 
 
 
  
 
 
 
 
 In [6]: 
 
 
     len  (  set  (  hallmark_gs  [  "HALLMARK_ESTROGEN_RESPONSE_EARLY"  ])  .  intersection  (  c6_gs  [  "MEK_UP.V1_DN"  ]))  /  len  (  hallmark_gs  [  "HALLMARK_ESTROGEN_RESPONSE_EARLY"  ]) 
  
 
 
 
 
 
 
 
 
 
 Out[6]: 
 
 0.15 
 
 
 
 
  
 
 
 
 
 In [7]: 
 
 
     brca_gdc_tpm 
  
 
 
 
 
 
 
 
 
 
 Out[7]: 
 
 
 
 
 
 
  
 TCGA-E2-A1L7 
 TCGA-AR-A0U0 
 TCGA-BH-A28O 
 TCGA-A2-A0D4 
 TCGA-E9-A1R4 
 TCGA-AO-A1KQ 
 TCGA-AC-A62V 
 TCGA-D8-A143 
 TCGA-A2-A0SV 
 TCGA-AN-A0XW 
 ... 
 TCGA-AC-A5EH 
 TCGA-A1-A0SD 
 TCGA-A2-A0SU 
 TCGA-E9-A1NI 
 TCGA-B6-A0RQ 
 TCGA-A2-A0CX 
 TCGA-A2-A25F 
 TCGA-AC-A23G 
 TCGA-E2-A15D 
 TCGA-OL-A5D7 
 
 
 gene_name 
  
  
  
  
  
  
  
  
  
  
  
  
  
  
  
  
  
  
  
  
  
 
 
 
 
 5S_rRNA 
 0.231289 
 0.204489 
 0.193633 
 0.256344 
 0.076333 
 0.134200 
 0.322033 
 0.059178 
 0.092778 
 0.140722 
 ... 
 0.0000 
 0.066067 
 0.245711 
 0.086689 
 0.0000 
 0.000000 
 0.000000 
 0.133544 
 0.0000 
 0.0000 
 
 
 5_8S_rRNA 
 0.000000 
 0.000000 
 0.000000 
 0.000000 
 0.000000 
 0.000000 
 0.000000 
 0.000000 
 0.000000 
 0.000000 
 ... 
 0.0000 
 0.069117 
 0.000000 
 0.000000 
 0.0000 
 0.000000 
 0.000000 
 0.000000 
 0.0000 
 0.0000 
 
 
 7SK 
 0.036586 
 0.000000 
 0.037600 
 0.000000 
 0.000000 
 0.041571 
 0.000000 
 0.000000 
 0.044886 
 0.128714 
 ... 
 0.0000 
 0.000000 
 0.000000 
 0.000000 
 0.0000 
 0.065329 
 0.024243 
 0.000000 
 0.0000 
 0.0000 
 
 
 A1BG 
 0.095400 
 0.048800 
 0.357700 
 0.384100 
 0.364200 
 0.233600 
 0.107600 
 0.067100 
 0.117100 
 0.369300 
 ... 
 0.1941 
 0.346800 
 0.097700 
 0.020700 
 0.2668 
 0.113000 
 0.180400 
 0.446000 
 0.3221 
 0.0560 
 
 
 A1BG-AS1 
 2.036100 
 0.693800 
 1.926900 
 2.128600 
 2.546700 
 1.959600 
 0.546300 
 0.377900 
 1.096000 
 1.870200 
 ... 
 1.7947 
 1.849500 
 0.949500 
 1.127500 
 2.3798 
 0.803400 
 0.741900 
 3.209600 
 2.1898 
 1.0393 
 
 
 ... 
 ... 
 ... 
 ... 
 ... 
 ... 
 ... 
 ... 
 ... 
 ... 
 ... 
 ... 
 ... 
 ... 
 ... 
 ... 
 ... 
 ... 
 ... 
 ... 
 ... 
 ... 
 
 
 ZZEF1 
 22.224500 
 15.184600 
 22.424000 
 6.723100 
 21.986200 
 5.789800 
 6.021400 
 11.884700 
 7.580600 
 8.623300 
 ... 
 13.4076 
 16.404400 
 7.027800 
 11.893900 
 18.7700 
 7.368500 
 23.281400 
 16.446400 
 21.7841 
 11.1431 
 
 
 ZZZ3 
 30.547300 
 25.071200 
 27.180200 
 15.913500 
 15.230100 
 10.108100 
 6.902900 
 25.557900 
 11.546500 
 19.725300 
 ... 
 16.8483 
 29.787200 
 28.208200 
 14.266900 
 28.4495 
 11.836500 
 26.301300 
 17.070800 
 26.1865 
 7.2553 
 
 
 hsa-mir-1253 
 0.000000 
 0.000000 
 0.000000 
 0.000000 
 0.000000 
 0.000000 
 0.000000 
 0.000000 
 0.000000 
 0.000000 
 ... 
 0.0000 
 0.000000 
 0.000000 
 0.000000 
 0.0000 
 0.000000 
 0.000000 
 0.000000 
 0.0000 
 0.0000 
 
 
 hsa-mir-423 
 0.000000 
 0.000000 
 0.000000 
 0.000000 
 0.000000 
 0.000000 
 0.000000 
 0.000000 
 0.000000 
 0.000000 
 ... 
 0.0000 
 0.000000 
 0.000000 
 0.000000 
 0.0000 
 0.000000 
 0.000000 
 0.000000 
 0.0000 
 0.0000 
 
 
 snoZ196 
 0.000000 
 0.000000 
 1.461000 
 0.000000 
 0.000000 
 0.807500 
 0.000000 
 0.000000 
 1.577800 
 0.000000 
 ... 
 1.7445 
 0.000000 
 0.000000 
 0.000000 
 0.0000 
 0.000000 
 0.953500 
 0.000000 
 0.7616 
 0.0000 
 
 
 
 59427 rows × 1095 columns 
 
 
 
 
 
  
 
 
 
 
 In [8]: 
 
 
     brca_hallmark_es   =   gsva_py2r  (  np  .  log2  (  brca_gdc_tpm  +  1  ),  hallmark_gs  ) 
 ov_hallmark_es   =   gsva_py2r  (  np  .  log2  (  ov_gdc_tpm  +  1  ),  hallmark_gs  ) 
 ucec_hallmark_es   =   gsva_py2r  (  np  .  log2  (  ucec_gdc_tpm  +  1  ),  hallmark_gs  ) 
 cesc_hallmark_es   =   gsva_py2r  (  np  .  log2  (  cesc_gdc_tpm  +  1  ),  hallmark_gs  ) 
  
 
 
 
 
 
 
 
 
 
  
 
 Converting df
Estimating GSVA scores for 50 gene sets.
Estimating ECDFs with Gaussian kernels
  |======================================================================| 100%

Converting df
Estimating GSVA scores for 50 gene sets.
Estimating ECDFs with Gaussian kernels
  |======================================================================| 100%

Converting df
Estimating GSVA scores for 50 gene sets.
Estimating ECDFs with Gaussian kernels
  |======================================================================| 100%

Converting df
Estimating GSVA scores for 50 gene sets.
Estimating ECDFs with Gaussian kernels
  |======================================================================| 100%

 
 
 
 
 
  
 
 
 
 
 In [10]: 
 
 
     brca_es   =   pd  .  DataFrame  (  brca_hallmark_es  [  "HALLMARK_ESTROGEN_RESPONSE_EARLY"  ]  .  values  ,   index  =  brca_hallmark_es  .  index  ,   columns  =  [  'EARLY'  ]) 
 ov_es   =   pd  .  DataFrame  (  ov_hallmark_es  [  "HALLMARK_ESTROGEN_RESPONSE_EARLY"  ]  .  values  ,   index  =  ov_hallmark_es  .  index  ,   columns  =  [  'EARLY'  ]) 
 ucec_es   =   pd  .  DataFrame  (  ucec_hallmark_es  [  "HALLMARK_ESTROGEN_RESPONSE_EARLY"  ]  .  values  ,   index  =  ucec_hallmark_es  .  index  ,   columns  =  [  'EARLY'  ]) 
 cesc_es   =   pd  .  DataFrame  (  cesc_hallmark_es  [  "HALLMARK_ESTROGEN_RESPONSE_EARLY"  ]  .  values  ,   index  =  cesc_hallmark_es  .  index  ,   columns  =  [  'EARLY'  ]) 
  
 
 
 
 
  
 
 
 
 
 In [9]: 
 
 
     with   pd  .  ExcelWriter  (  'Table S1.xlsx'  )   as   writer  : 
     brca_es  .  rename  (  columns  =  {  "EARLY"  :  "EERES"  })  .  to_excel  (  writer  ,   "BRCA"  ) 
     ov_es  .  rename  (  columns  =  {  "EARLY"  :  "EERES"  })  .  to_excel  (  writer  ,   "OV"  ) 
     ucec_es  .  rename  (  columns  =  {  "EARLY"  :  "EERES"  })  .  to_excel  (  writer  ,   "UCEC"  ) 
     cesc_es  .  rename  (  columns  =  {  "EARLY"  :  "EERES"  })  .  to_excel  (  writer  ,   "CESC"  ) 
  
 
 
 
 
 
 
 
 
 
  
  
 Figure 1 ¶  
 
 
 
  
 
 
 
 
 In [11]: 
 
 
     def   finding_best_es_for_survival  (  es_dfs_df  ,   es_dss_df  ,   es_df  ): 
     bestes  =  None 
     bestp  =  1 
     sp   =   [] 

     for   i   in   np  .  arange  (  es_df  [  "EARLY"  ]  .  min  (),   es_df  [  "EARLY"  ]  .  max  (),   0.01  ): 
         results_dfs   =   km  .  fit  (  es_dfs_df  [  f  "DFS_MONTHS"  ],   es_dfs_df  [  f  "DFS_STATUS"  ],   (  es_dfs_df  [  "EARLY"  ]  >  i  )  .  apply  (  lambda   x  :   "High EERES"   if   x   else   "Low EERES"  )) 
         results_dss   =   km  .  fit  (  es_dss_df  [  f  "DSS_MONTHS"  ],   es_dss_df  [  f  "DSS_STATUS"  ],   (  es_dss_df  [  "EARLY"  ]  >  i  )  .  apply  (  lambda   x  :   "High EERES"   if   x   else   "Low EERES"  )) 
         p   =   (  results_dfs  [  'logrank_P'  ]  +  results_dss  [  'logrank_P'  ])  /  2 
         if   p  <  bestp  : 
             bestes   =   i 
             bestp   =   (  results_dfs  [  'logrank_P'  ]  +  results_dss  [  'logrank_P'  ])  /  2 
     print  (  "EERES Threshold"  ,   bestes  ) 
     print  (  "n"  ,   len  (  es_df  )) 
     print  (  "n >threshold"  ,   (  es_df  [  "EARLY"  ]  >  bestes  )  .  sum  ()) 
     print  (  "n <=threshold"  ,   (  es_df  [  "EARLY"  ]  <=  bestes  )  .  sum  ()) 
     results   =   km  .  fit  (  es_dfs_df  [  f  "DFS_MONTHS"  ],   es_dfs_df  [  f  "DFS_STATUS"  ],   (  es_dfs_df  [  "EARLY"  ]  >  bestes  )  .  apply  (  lambda   x  :   "High EERES"   if   x   else   "Low EESRS"  )) 
     km  .  plot  (  results  ,   full_ylim  =  True  ,   y_percentage  =  True  ,   fontsize  =  15  ) 
     plt  .  show  () 
     results   =   km  .  fit  (  es_dss_df  [  f  "DSS_MONTHS"  ],   es_dss_df  [  f  "DSS_STATUS"  ],   (  es_dss_df  [  "EARLY"  ]  >  bestes  )  .  apply  (  lambda   x  :   "High EERES"   if   x   else   "Low EESRS"  )) 
     km  .  plot  (  results  ,   full_ylim  =  True  ,   y_percentage  =  True  ,   fontsize  =  15  ) 
     plt  .  show  () 
     return   bestes 
    
 print  (  "BRCA"  )     
 brca_dfs_es   =   brca_dfs  .  join  (  brca_es  ,   how  =  'inner'  )  .  dropna  () 
 brca_dss_es   =   brca_dss  .  join  (  brca_es  ,   how  =  'inner'  )  .  dropna  () 
 brca_bestes   =   finding_best_es_for_survival  (  brca_dfs_es  ,   brca_dss_es  ,   brca_es  ) 
 print  (  "OV"  )  
 ov_dfs_es   =   ov_dfs  .  join  (  ov_es  ,   how  =  'inner'  )  .  dropna  () 
 ov_dss_es   =   ov_dss  .  join  (  ov_es  ,   how  =  'inner'  )  .  dropna  () 
 ov_bestes   =   finding_best_es_for_survival  (  ov_dfs_es  ,   ov_dss_es  ,   ov_es  ) 
 print  (  "UCEC"  )  
 ucec_dfs_es   =   ucec_dfs  .  join  (  ucec_es  ,   how  =  'inner'  )  .  dropna  () 
 ucec_dss_es   =   ucec_dss  .  join  (  ucec_es  ,   how  =  'inner'  )  .  dropna  () 
 ucec_bestes   =   finding_best_es_for_survival  (  ucec_dfs_es  ,   ucec_dss_es  ,   ucec_es  ) 
 print  (  "CESC"  )  
 cesc_dfs_es   =   cesc_dfs  .  join  (  cesc_es  ,   how  =  'inner'  )  .  dropna  () 
 cesc_dss_es   =   cesc_dss  .  join  (  cesc_es  ,   how  =  'inner'  )  .  dropna  () 
 cesc_bestes   =   finding_best_es_for_survival  (  cesc_dfs_es  ,   cesc_dss_es  ,   cesc_es  ) 
  
 
 
 
 
 
 
 
 
 
  
 
 BRCA
EERES Threshold -0.051194787594808
n 1095
n >threshold 610
n <=threshold 485
 
 
 
 
  
 
 
 
 
 
  
 
 
 
 
 
  
 
 OV
EERES Threshold -0.18398784249546785
n 378
n >threshold 263
n <=threshold 115
 
 
 
 
  
 
 
 
 
 
  
 
 
 
 
 
  
 
 UCEC
EERES Threshold 0.15745491375942877
n 557
n >threshold 147
n <=threshold 410
 
 
 
 
  
 
 
 
 
 
  
 
 
 
 
 
  
 
 CESC
EERES Threshold -0.2282193398849608
n 304
n >threshold 248
n <=threshold 56
 
 
 
 
  
 
 
 
 
 
  
 
 
 
 
 
 
  
 
 
 
 
 In [11]: 
 
 
     tmp   =   brca_dfs_es  [[  "DFS_STATUS"  ,   "DFS_MONTHS"  ,   "EARLY"  ]] 
 tmp  [  "EARLY"  ]   =   tmp  [  "EARLY"  ]  .  apply  (  lambda   x  :   1   if   x  >-  0.051194787594808   else   0  ) 
 tmp  .  to_csv  (  "brca_dfs.csv"  ) 
 tmp   =   brca_dss_es  [[  "DSS_STATUS"  ,   "DSS_MONTHS"  ,   "EARLY"  ]] 
 tmp  [  "EARLY"  ]   =   tmp  [  "EARLY"  ]  .  apply  (  lambda   x  :   1   if   x  >-  0.051194787594808   else   0  ) 
 tmp  .  to_csv  (  "brca_dss.csv"  ) 

 tmp   =   ov_dfs_es  [[  "DFS_STATUS"  ,   "DFS_MONTHS"  ,   "EARLY"  ]] 
 tmp  [  "EARLY"  ]   =   tmp  [  "EARLY"  ]  .  apply  (  lambda   x  :   1   if   x  >-  0.18398784249546785   else   0  ) 
 tmp  .  to_csv  (  "ov_dfs.csv"  ) 
 tmp   =   ov_dss_es  [[  "DSS_STATUS"  ,   "DSS_MONTHS"  ,   "EARLY"  ]] 
 tmp  [  "EARLY"  ]   =   tmp  [  "EARLY"  ]  .  apply  (  lambda   x  :   1   if   x  >-  0.18398784249546785   else   0  ) 
 tmp  .  to_csv  (  "ov_dss.csv"  ) 

 tmp   =   ucec_dfs_es  [[  "DFS_STATUS"  ,   "DFS_MONTHS"  ,   "EARLY"  ]] 
 tmp  [  "EARLY"  ]   =   tmp  [  "EARLY"  ]  .  apply  (  lambda   x  :   1   if   x  >  0.15745491375942877   else   0  ) 
 tmp  .  to_csv  (  "ucec_dfs.csv"  ) 
 tmp   =   ucec_dss_es  [[  "DSS_STATUS"  ,   "DSS_MONTHS"  ,   "EARLY"  ]] 
 tmp  [  "EARLY"  ]   =   tmp  [  "EARLY"  ]  .  apply  (  lambda   x  :   1   if   x  >  0.15745491375942877   else   0  ) 
 tmp  .  to_csv  (  "ucec_dss.csv"  ) 

 tmp   =   cesc_dfs_es  [[  "DFS_STATUS"  ,   "DFS_MONTHS"  ,   "EARLY"  ]] 
 tmp  [  "EARLY"  ]   =   tmp  [  "EARLY"  ]  .  apply  (  lambda   x  :   1   if   x  >-  0.2282193398849608   else   0  ) 
 tmp  .  to_csv  (  "cesc_dfs.csv"  ) 
 tmp   =   cesc_dss_es  [[  "DSS_STATUS"  ,   "DSS_MONTHS"  ,   "EARLY"  ]] 
 tmp  [  "EARLY"  ]   =   tmp  [  "EARLY"  ]  .  apply  (  lambda   x  :   1   if   x  >-  0.2282193398849608   else   0  ) 
 tmp  .  to_csv  (  "cesc_dss.csv"  ) 
  
 
 
 
 
 
 
 
 
 
  
 
 /tmp/ipykernel_30800/682250153.py:2: SettingWithCopyWarning: 
A value is trying to be set on a copy of a slice from a DataFrame.
Try using .loc[row_indexer,col_indexer] = value instead

See the caveats in the documentation: https://pandas.pydata.org/pandas-docs/stable/user_guide/indexing.html#returning-a-view-versus-a-copy
  tmp["EARLY"] = tmp["EARLY"].apply(lambda x: 1 if x>-0.051194787594808 else 0)
/tmp/ipykernel_30800/682250153.py:5: SettingWithCopyWarning: 
A value is trying to be set on a copy of a slice from a DataFrame.
Try using .loc[row_indexer,col_indexer] = value instead

See the caveats in the documentation: https://pandas.pydata.org/pandas-docs/stable/user_guide/indexing.html#returning-a-view-versus-a-copy
  tmp["EARLY"] = tmp["EARLY"].apply(lambda x: 1 if x>-0.051194787594808 else 0)
/tmp/ipykernel_30800/682250153.py:9: SettingWithCopyWarning: 
A value is trying to be set on a copy of a slice from a DataFrame.
Try using .loc[row_indexer,col_indexer] = value instead

See the caveats in the documentation: https://pandas.pydata.org/pandas-docs/stable/user_guide/indexing.html#returning-a-view-versus-a-copy
  tmp["EARLY"] = tmp["EARLY"].apply(lambda x: 1 if x>-0.18398784249546785 else 0)
/tmp/ipykernel_30800/682250153.py:12: SettingWithCopyWarning: 
A value is trying to be set on a copy of a slice from a DataFrame.
Try using .loc[row_indexer,col_indexer] = value instead

See the caveats in the documentation: https://pandas.pydata.org/pandas-docs/stable/user_guide/indexing.html#returning-a-view-versus-a-copy
  tmp["EARLY"] = tmp["EARLY"].apply(lambda x: 1 if x>-0.18398784249546785 else 0)
/tmp/ipykernel_30800/682250153.py:16: SettingWithCopyWarning: 
A value is trying to be set on a copy of a slice from a DataFrame.
Try using .loc[row_indexer,col_indexer] = value instead

See the caveats in the documentation: https://pandas.pydata.org/pandas-docs/stable/user_guide/indexing.html#returning-a-view-versus-a-copy
  tmp["EARLY"] = tmp["EARLY"].apply(lambda x: 1 if x>0.15745491375942877 else 0)
/tmp/ipykernel_30800/682250153.py:19: SettingWithCopyWarning: 
A value is trying to be set on a copy of a slice from a DataFrame.
Try using .loc[row_indexer,col_indexer] = value instead

See the caveats in the documentation: https://pandas.pydata.org/pandas-docs/stable/user_guide/indexing.html#returning-a-view-versus-a-copy
  tmp["EARLY"] = tmp["EARLY"].apply(lambda x: 1 if x>0.15745491375942877 else 0)
/tmp/ipykernel_30800/682250153.py:23: SettingWithCopyWarning: 
A value is trying to be set on a copy of a slice from a DataFrame.
Try using .loc[row_indexer,col_indexer] = value instead

See the caveats in the documentation: https://pandas.pydata.org/pandas-docs/stable/user_guide/indexing.html#returning-a-view-versus-a-copy
  tmp["EARLY"] = tmp["EARLY"].apply(lambda x: 1 if x>-0.2282193398849608 else 0)
/tmp/ipykernel_30800/682250153.py:26: SettingWithCopyWarning: 
A value is trying to be set on a copy of a slice from a DataFrame.
Try using .loc[row_indexer,col_indexer] = value instead

See the caveats in the documentation: https://pandas.pydata.org/pandas-docs/stable/user_guide/indexing.html#returning-a-view-versus-a-copy
  tmp["EARLY"] = tmp["EARLY"].apply(lambda x: 1 if x>-0.2282193398849608 else 0)
 
 
 
 
 
  
 
 
 
 
 In [13]: 
 
 
     def   plotting_esr1_eeres_scatter_spearman  (  esr1_es_df  ):     
     plt  .  scatter  (  np  .  log2  (  esr1_es_df  [  "ESR1"  ]   +   1  ),   esr1_es_df  [  "EARLY"  ]) 
     plt  .  xlabel  (  "log2[ESR1+1]"  ,   weight  =  "bold"  ,   fontsize  =  12  ,   labelpad  =  0  ) 
     plt  .  ylabel  (  "EERES"  ,   weight  =  "bold"  ,   fontsize  =  12  ,   labelpad  =-  5  ) 
     sr  ,   sp   =   stats  .  spearmanr  (  np  .  log2  (  esr1_es_df  [  "ESR1"  ]),   esr1_es_df  [  "EARLY"  ]) 
     plt  .  title  (  f  "Spearman R=  {  sr  :  .3f  }  , p=  {  sp  :  .3e  }  "  ,   weight  =  "bold"  ) 
     plt  .  show  () 
    
 brca_es   =   pd  .  DataFrame  (  brca_hallmark_es  [  "HALLMARK_ESTROGEN_RESPONSE_EARLY"  ]  .  values  ,   index  =  brca_hallmark_es  .  index  ,   columns  =  [  "EARLY"  ]) 
 brca_esr1_es_df   =   brca_gdc_tpm  .  transpose  ()[[  "ESR1"  ]]  .  join  (  brca_es  ,   how  =  'inner'  ) 
 plotting_esr1_eeres_scatter_spearman  (  brca_esr1_es_df  ) 

 ov_es   =   pd  .  DataFrame  (  ov_hallmark_es  [  "HALLMARK_ESTROGEN_RESPONSE_EARLY"  ]  .  values  ,   index  =  ov_hallmark_es  .  index  ,   columns  =  [  "EARLY"  ]) 
 ov_esr1_es_df   =   ov_gdc_tpm  .  transpose  ()[[  "ESR1"  ]]  .  join  (  ov_es  ,   how  =  'inner'  ) 
 plotting_esr1_eeres_scatter_spearman  (  ov_esr1_es_df  ) 

 ucec_es   =   pd  .  DataFrame  (  ucec_hallmark_es  [  "HALLMARK_ESTROGEN_RESPONSE_EARLY"  ]  .  values  ,   index  =  ucec_hallmark_es  .  index  ,   columns  =  [  "EARLY"  ]) 
 ucec_esr1_es_df   =   ucec_gdc_tpm  .  transpose  ()[[  "ESR1"  ]]  .  join  (  ucec_es  ,   how  =  'inner'  ) 
 plotting_esr1_eeres_scatter_spearman  (  ucec_esr1_es_df  ) 

 cesc_es   =   pd  .  DataFrame  (  cesc_hallmark_es  [  "HALLMARK_ESTROGEN_RESPONSE_EARLY"  ]  .  values  ,   index  =  cesc_hallmark_es  .  index  ,   columns  =  [  "EARLY"  ]) 
 cesc_esr1_es_df   =   cesc_gdc_tpm  .  transpose  ()[[  "ESR1"  ]]  .  join  (  cesc_es  ,   how  =  'inner'  ) 
 plotting_esr1_eeres_scatter_spearman  (  cesc_esr1_es_df  ) 
  
 
 
 
 
 
 
 
 
 
  
 
 
 
 
 
  
 
 
 
 
 
  
 
 
 
 
 
  
 
 
 
 
 
 
 
 
 
 
 
  
  
 Figure 2 ¶   ESR1 and EERES on Survival of hormonal therapy treated patients ¶  
 
 
 
  
 
 
 
 
 In [14]: 
 
 
     q_groups   =   {} 
 for   q   in   np  .  arange  (  0.1  ,  1  ,  0.05  ): 
     br_clinical_gdc   =   pd  .  read_table  (  "Data/gdc/TCGA-BRCA/clinical/nationwidechildrens.org_clinical_patient_brca.txt"  ,   skiprows  =  1  ,   header  =  0  ,   index_col  =  1  )  .  iloc  [  1  :] 
     brca_es   =   pd  .  DataFrame  (  brca_hallmark_es  [  "HALLMARK_ESTROGEN_RESPONSE_EARLY"  ]  .  values  ,   index  =  brca_hallmark_es  .  index  ,   columns  =  [  "EARLY"  ]) 
     brca_esr1_es_df   =   brca_gdc_tpm  .  transpose  ()[[  "ESR1"  ]]  .  join  (  brca_es  ,   how  =  'inner'  ) 
     brca_erpos_her2neg_index   =   br_clinical_gdc  [((  br_clinical_gdc  [  "breast_carcinoma_estrogen_receptor_status"  ]  ==  "Positive"  ))  &  (  br_clinical_gdc  [  "lab_proc_her2_neu_immunohistochemistry_receptor_status"  ]  ==  "Negative"  )]  .  index 
     brca_erpos_her2neg_index   =   [  i  .  replace  (  '.'  ,  '-'  )   for   i   in   brca_erpos_her2neg_index  ] 
     brca_erpos_her2neg_index_esr1_index   =   set  (  brca_erpos_her2neg_index  )  .  intersection  (  brca_esr1_es_df  .  index  ) 
     print  (  "n:"  ,   len  (  brca_erpos_her2neg_index_esr1_index  )) 
     brca_esr1_es_df   =   brca_esr1_es_df  .  loc  [  list  (  brca_erpos_her2neg_index_esr1_index  )] 
     BRCA_ESR1_Threshold   =   brca_esr1_es_df  [  "ESR1"  ]  .  quantile  (  q  ) 
     BRCA_EERES_Threshold   =   brca_esr1_es_df  [  "EARLY"  ]  .  quantile  (  q  ) 
     print  (  BRCA_ESR1_Threshold  ) 
     print  (  BRCA_EERES_Threshold  ) 
    
     plotting_scatter_esr1_es  (  brca_esr1_es_df  ,   BRCA_ESR1_Threshold  ,   BRCA_EERES_Threshold  ) 
    
     brca_groups   =   grouping4  (  brca_esr1_es_df  ,   BRCA_ESR1_Threshold  ,   BRCA_EERES_Threshold  ) 

     q_groups  [  q  ]   =   {  'BRCA_ESR1_Threshold'  :  BRCA_ESR1_Threshold  ,   'BRCA_EERES_Threshold'  :  BRCA_EERES_Threshold  } 
  
 
 
 
 
 
 
 
 
 
  
 
 n: 436
29.35475
-0.28884320118370976
 
 
 
 
  
 
 
 
 
 
  
 
 n: 436
43.950675000000004
-0.20438048637962367
 
 
 
 
  
 
 
 
 
 
  
 
 n: 436
58.74400000000001
-0.15194432395471977
 
 
 
 
  
 
 
 
 
 
  
 
 n: 436
68.10275
-0.08065019107565878
 
 
 
 
  
 
 
 
 
 
  
 
 n: 436
77.9179
-0.025146768801859266
 
 
 
 
  
 
 
 
 
 
  
 
 n: 436
89.568425
0.015048116919465372
 
 
 
 
  
 
 
 
 
 
  
 
 n: 436
102.37820000000004
0.05880599028967579
 
 
 
 
  
 
 
 
 
 
  
 
 n: 436
123.099225
0.1061000830013521
 
 
 
 
  
 
 
 
 
 
  
 
 n: 436
140.102
0.14691609120149926
 
 
 
 
  
 
 
 
 
 
  
 
 n: 436
153.9781250000001
0.17969457947342965
 
 
 
 
  
 
 
 
 
 
  
 
 n: 436
173.33830000000023
0.20547056079620443
 
 
 
 
  
 
 
 
 
 
  
 
 n: 436
199.29877500000003
0.21932558344087438
 
 
 
 
  
 
 
 
 
 
  
 
 n: 436
226.31265000000008
0.24852481578754917
 
 
 
 
  
 
 
 
 
 
  
 
 n: 436
256.38257500000014
0.2821959900735944
 
 
 
 
  
 
 
 
 
 
  
 
 n: 436
296.9775000000001
0.31572829628565613
 
 
 
 
  
 
 
 
 
 
  
 
 n: 436
338.723425
0.35571723332676947
 
 
 
 
  
 
 
 
 
 
  
 
 n: 436
381.8058500000002
0.3883947642399483
 
 
 
 
  
 
 
 
 
 
  
 
 n: 436
493.54282500000215
0.4366569137147916
 
 
 
 
  
 
 
 
 
 
 
  
 
 
 
 
 In [12]: 
 
 
     brca_endocrine_index   =   pd  .  read_table  (  "Data/brca_tcga_pan_can_atlas_2018/brca_tcga_pan_can_atlas_2018_clinical_data_endocrineTreated.tsv"  ,   index_col  =  1  )  .  index 
  
 
 
 
 
  
 
 
 
 
 In [15]: 
 
 
     for   q   in   q_groups  : 
     print  (  q  ) 
     BRCA_ESR1_Threshold   =   q_groups  [  q  ][  'BRCA_ESR1_Threshold'  ] 
     BRCA_EERES_Threshold   =   q_groups  [  q  ][  'BRCA_EERES_Threshold'  ] 
    
     print  (  "PFS"  ) 
    
     br_pfs_es   =   brca_pfs  .  join  (  brca_esr1_es_df  ,   how  =  'inner'  ) 
     br_pfs_es   =   br_pfs_es  [  ~  br_pfs_es  .  index  .  duplicated  (  keep  =  "first"  )] 
     plotting_2groups  (  br_pfs_es  ,   "PFS"  ,   BRCA_ESR1_Threshold  ,   BRCA_EERES_Threshold  ) 
     plotting_4groups  (  br_pfs_es  ,   "PFS"  ,   BRCA_ESR1_Threshold  ,   BRCA_EERES_Threshold  ) 
    
     print  (  "OS"  ) 
    
     br_os_es   =   brca_os  .  join  (  brca_esr1_es_df  ,   how  =  'inner'  ) 
     br_os_es   =   br_os_es  [  ~  br_os_es  .  index  .  duplicated  (  keep  =  "first"  )] 
     plotting_2groups  (  br_os_es  ,   "OS"  ,   BRCA_ESR1_Threshold  ,   BRCA_EERES_Threshold  ) 
     plotting_4groups  (  br_os_es  ,   "OS"  ,   BRCA_ESR1_Threshold  ,   BRCA_EERES_Threshold  ) 
    
     print  (  "DFS"  ) 
    
     br_dfs_es   =   brca_dfs  .  join  (  brca_esr1_es_df  ,   how  =  'inner'  ) 
     br_dfs_es   =   br_dfs_es  [  ~  br_dfs_es  .  index  .  duplicated  (  keep  =  "first"  )] 
     plotting_2groups  (  br_dfs_es  ,   "DFS"  ,   BRCA_ESR1_Threshold  ,   BRCA_EERES_Threshold  ) 
     plotting_4groups  (  br_dfs_es  ,   "DFS"  ,   BRCA_ESR1_Threshold  ,   BRCA_EERES_Threshold  ) 
    
     print  (  "DSS"  ) 
    
     br_dss_es   =   brca_dss  .  join  (  brca_esr1_es_df  ,   how  =  'inner'  ) 
     br_dss_es   =   br_dss_es  [  ~  br_dss_es  .  index  .  duplicated  (  keep  =  "first"  )] 
     plotting_2groups  (  br_dss_es  ,   "DSS"  ,   BRCA_ESR1_Threshold  ,   BRCA_EERES_Threshold  ) 
     plotting_4groups  (  br_dss_es  ,   "DSS"  ,   BRCA_ESR1_Threshold  ,   BRCA_EERES_Threshold  ) 
  
 
 
 
 
 
 
 
 
 
  
 
 0.1
PFS
 
 
 
 
  
 
 
 
 
 
  
 
 
 
 
 
  
 
 ESR1_low_EERES_low vs ESR1_low_EERES_high 0.8598786805963252
ESR1_low_EERES_low vs ESR1_high_EERES_low 0.5201102619875551
ESR1_low_EERES_low vs ESR1_high_EERES_high 0.6329711579191577
ESR1_low_EERES_high vs ESR1_high_EERES_low 0.7482941382955608
ESR1_low_EERES_high vs ESR1_high_EERES_high 0.737526064124406
ESR1_high_EERES_low vs ESR1_high_EERES_high 0.8538484019660898
                     ESR1_low_EERES_low ESR1_low_EERES_high  \
ESR1_low_EERES_low                  NaN            0.859879   
ESR1_low_EERES_high                 NaN                 NaN   
ESR1_high_EERES_low                 NaN                 NaN   
ESR1_high_EERES_high                NaN                 NaN   

                     ESR1_high_EERES_low ESR1_high_EERES_high  
ESR1_low_EERES_low               0.52011             0.632971  
ESR1_low_EERES_high             0.748294             0.737526  
ESR1_high_EERES_low                  NaN             0.853848  
ESR1_high_EERES_high                 NaN                  NaN  
 
 
 
 
  
 
 
 
 
 
  
 
 OS
 
 
 
 
  
 
 
 
 
 
  
 
 
 
 
 
  
 
 ESR1_low_EERES_low vs ESR1_low_EERES_high 0.9555688034538364
ESR1_low_EERES_low vs ESR1_high_EERES_low 0.6612251134791733
ESR1_low_EERES_low vs ESR1_high_EERES_high 0.34691325795723904
ESR1_low_EERES_high vs ESR1_high_EERES_low 0.625325358271994
ESR1_low_EERES_high vs ESR1_high_EERES_high 0.6360814577004981
ESR1_high_EERES_low vs ESR1_high_EERES_high 0.9875905429808715
                     ESR1_low_EERES_low ESR1_low_EERES_high  \
ESR1_low_EERES_low                  NaN            0.955569   
ESR1_low_EERES_high                 NaN                 NaN   
ESR1_high_EERES_low                 NaN                 NaN   
ESR1_high_EERES_high                NaN                 NaN   

                     ESR1_high_EERES_low ESR1_high_EERES_high  
ESR1_low_EERES_low              0.661225             0.346913  
ESR1_low_EERES_high             0.625325             0.636081  
ESR1_high_EERES_low                  NaN             0.987591  
ESR1_high_EERES_high                 NaN                  NaN  
 
 
 
 
  
 
 
 
 
 
  
 
 DFS
 
 
 
 
  
 
 
 
 
 
  
 
 
 
 
 
  
 
 ESR1_low_EERES_low vs ESR1_low_EERES_high 0.7838594446269418
ESR1_low_EERES_low vs ESR1_high_EERES_low 0.20438922788614522
ESR1_low_EERES_low vs ESR1_high_EERES_high 0.9631777045647636
ESR1_low_EERES_high vs ESR1_high_EERES_low 0.18048579714551144
ESR1_low_EERES_high vs ESR1_high_EERES_high 0.9434151447359935
ESR1_high_EERES_low vs ESR1_high_EERES_high 0.18551959439733268
                     ESR1_low_EERES_low ESR1_low_EERES_high  \
ESR1_low_EERES_low                  NaN            0.783859   
ESR1_low_EERES_high                 NaN                 NaN   
ESR1_high_EERES_low                 NaN                 NaN   
ESR1_high_EERES_high                NaN                 NaN   

                     ESR1_high_EERES_low ESR1_high_EERES_high  
ESR1_low_EERES_low              0.204389             0.963178  
ESR1_low_EERES_high             0.180486             0.943415  
ESR1_high_EERES_low                  NaN              0.18552  
ESR1_high_EERES_high                 NaN                  NaN  
 
 
 
 
  
 
 
 
 
 
  
 
 DSS
 
 
 
 
  
 
 
 
 
 
  
 
 
 
 
 
  
 
 ESR1_low_EERES_low vs ESR1_low_EERES_high 1.0
ESR1_low_EERES_low vs ESR1_high_EERES_low 0.1967056024589432
ESR1_low_EERES_low vs ESR1_high_EERES_high 0.14433386571613221
ESR1_low_EERES_high vs ESR1_high_EERES_low 0.14103164052071204
ESR1_low_EERES_high vs ESR1_high_EERES_high 0.21547136682388363
ESR1_high_EERES_low vs ESR1_high_EERES_high 0.9121914800870119
                     ESR1_low_EERES_low ESR1_low_EERES_high  \
ESR1_low_EERES_low                  NaN                 1.0   
ESR1_low_EERES_high                 NaN                 NaN   
ESR1_high_EERES_low                 NaN                 NaN   
ESR1_high_EERES_high                NaN                 NaN   

                     ESR1_high_EERES_low ESR1_high_EERES_high  
ESR1_low_EERES_low              0.196706             0.144334  
ESR1_low_EERES_high             0.141032             0.215471  
ESR1_high_EERES_low                  NaN             0.912191  
ESR1_high_EERES_high                 NaN                  NaN  
 
 
 
 
  
 
 
 
 
 
  
 
 0.15000000000000002
PFS
 
 
 
 
  
 
 
 
 
 
  
 
 
 
 
 
  
 
 ESR1_low_EERES_low vs ESR1_low_EERES_high 0.9200218606235004
ESR1_low_EERES_low vs ESR1_high_EERES_low 0.867793323292622
ESR1_low_EERES_low vs ESR1_high_EERES_high 0.873827633162038
ESR1_low_EERES_high vs ESR1_high_EERES_low 0.9436849407075355
ESR1_low_EERES_high vs ESR1_high_EERES_high 0.8050812363068717
ESR1_high_EERES_low vs ESR1_high_EERES_high 0.8329214185252707
                     ESR1_low_EERES_low ESR1_low_EERES_high  \
ESR1_low_EERES_low                  NaN            0.920022   
ESR1_low_EERES_high                 NaN                 NaN   
ESR1_high_EERES_low                 NaN                 NaN   
ESR1_high_EERES_high                NaN                 NaN   

                     ESR1_high_EERES_low ESR1_high_EERES_high  
ESR1_low_EERES_low              0.867793             0.873828  
ESR1_low_EERES_high             0.943685             0.805081  
ESR1_high_EERES_low                  NaN             0.832921  
ESR1_high_EERES_high                 NaN                  NaN  
 
 
 
 
  
 
 
 
 
 
  
 
 OS
 
 
 
 
  
 
 
 
 
 
  
 
 
 
 
 
  
 
 ESR1_low_EERES_low vs ESR1_low_EERES_high 0.7124875507937802
ESR1_low_EERES_low vs ESR1_high_EERES_low 0.8548810264109633
ESR1_low_EERES_low vs ESR1_high_EERES_high 0.21637174668544573
ESR1_low_EERES_high vs ESR1_high_EERES_low 0.7672728860386844
ESR1_low_EERES_high vs ESR1_high_EERES_high 0.36007983911830976
ESR1_high_EERES_low vs ESR1_high_EERES_high 0.6435079866012261
                     ESR1_low_EERES_low ESR1_low_EERES_high  \
ESR1_low_EERES_low                  NaN            0.712488   
ESR1_low_EERES_high                 NaN                 NaN   
ESR1_high_EERES_low                 NaN                 NaN   
ESR1_high_EERES_high                NaN                 NaN   

                     ESR1_high_EERES_low ESR1_high_EERES_high  
ESR1_low_EERES_low              0.854881             0.216372  
ESR1_low_EERES_high             0.767273              0.36008  
ESR1_high_EERES_low                  NaN             0.643508  
ESR1_high_EERES_high                 NaN                  NaN  
 
 
 
 
  
 
 
 
 
 
  
 
 DFS
 
 
 
 
  
 
 
 
 
 
  
 
 
 
 
 
  
 
 ESR1_low_EERES_low vs ESR1_low_EERES_high 0.8695165528479848
ESR1_low_EERES_low vs ESR1_high_EERES_low 0.47992952660517163
ESR1_low_EERES_low vs ESR1_high_EERES_high 0.7580653318841046
ESR1_low_EERES_high vs ESR1_high_EERES_low 0.3573081766899927
ESR1_low_EERES_high vs ESR1_high_EERES_high 0.7364660272412553
ESR1_high_EERES_low vs ESR1_high_EERES_high 0.6073074165485859
                     ESR1_low_EERES_low ESR1_low_EERES_high  \
ESR1_low_EERES_low                  NaN            0.869517   
ESR1_low_EERES_high                 NaN                 NaN   
ESR1_high_EERES_low                 NaN                 NaN   
ESR1_high_EERES_high                NaN                 NaN   

                     ESR1_high_EERES_low ESR1_high_EERES_high  
ESR1_low_EERES_low               0.47993             0.758065  
ESR1_low_EERES_high             0.357308             0.736466  
ESR1_high_EERES_low                  NaN             0.607307  
ESR1_high_EERES_high                 NaN                  NaN  
 
 
 
 
  
 
 
 
 
 
  
 
 DSS
 
 
 
 
  
 
 
 
 
 
  
 
 
 
 
 
  
 
 ESR1_low_EERES_low vs ESR1_low_EERES_high 0.3906743460235679
ESR1_low_EERES_low vs ESR1_high_EERES_low 0.5741490727971621
ESR1_low_EERES_low vs ESR1_high_EERES_high 0.14003613914740343
ESR1_low_EERES_high vs ESR1_high_EERES_low 1.0
ESR1_low_EERES_high vs ESR1_high_EERES_high 0.13131342022078188
ESR1_high_EERES_low vs ESR1_high_EERES_high 0.1990545844748189
                     ESR1_low_EERES_low ESR1_low_EERES_high  \
ESR1_low_EERES_low                  NaN            0.390674   
ESR1_low_EERES_high                 NaN                 NaN   
ESR1_high_EERES_low                 NaN                 NaN   
ESR1_high_EERES_high                NaN                 NaN   

                     ESR1_high_EERES_low ESR1_high_EERES_high  
ESR1_low_EERES_low              0.574149             0.140036  
ESR1_low_EERES_high                  1.0             0.131313  
ESR1_high_EERES_low                  NaN             0.199055  
ESR1_high_EERES_high                 NaN                  NaN  
 
 
 
 
  
 
 
 
 
 
  
 
 0.20000000000000004
PFS
 
 
 
 
  
 
 
 
 
 
  
 
 
 
 
 
  
 
 ESR1_low_EERES_low vs ESR1_low_EERES_high 0.6622116249286212
ESR1_low_EERES_low vs ESR1_high_EERES_low 0.5283765688460079
ESR1_low_EERES_low vs ESR1_high_EERES_high 0.9082863220968839
ESR1_low_EERES_high vs ESR1_high_EERES_low 0.5435899146780092
ESR1_low_EERES_high vs ESR1_high_EERES_high 0.4987389752556778
ESR1_high_EERES_low vs ESR1_high_EERES_high 0.4315892982097067
                     ESR1_low_EERES_low ESR1_low_EERES_high  \
ESR1_low_EERES_low                  NaN            0.662212   
ESR1_low_EERES_high                 NaN                 NaN   
ESR1_high_EERES_low                 NaN                 NaN   
ESR1_high_EERES_high                NaN                 NaN   

                     ESR1_high_EERES_low ESR1_high_EERES_high  
ESR1_low_EERES_low              0.528377             0.908286  
ESR1_low_EERES_high              0.54359             0.498739  
ESR1_high_EERES_low                  NaN             0.431589  
ESR1_high_EERES_high                 NaN                  NaN  
 
 
 
 
  
 
 
 
 
 
  
 
 OS
 
 
 
 
  
 
 
 
 
 
  
 
 
 
 
 
  
 
 ESR1_low_EERES_low vs ESR1_low_EERES_high 0.15219900983806545
ESR1_low_EERES_low vs ESR1_high_EERES_low 0.8686215375739792
ESR1_low_EERES_low vs ESR1_high_EERES_high 0.4436500841749935
ESR1_low_EERES_high vs ESR1_high_EERES_low 0.2539255742278348
ESR1_low_EERES_high vs ESR1_high_EERES_high 0.06975060098670358
ESR1_high_EERES_low vs ESR1_high_EERES_high 0.38875089045625855
                     ESR1_low_EERES_low ESR1_low_EERES_high  \
ESR1_low_EERES_low                  NaN            0.152199   
ESR1_low_EERES_high                 NaN                 NaN   
ESR1_high_EERES_low                 NaN                 NaN   
ESR1_high_EERES_high                NaN                 NaN   

                     ESR1_high_EERES_low ESR1_high_EERES_high  
ESR1_low_EERES_low              0.868622              0.44365  
ESR1_low_EERES_high             0.253926             0.069751  
ESR1_high_EERES_low                  NaN             0.388751  
ESR1_high_EERES_high                 NaN                  NaN  
 
 
 
 
  
 
 
 
 
 
  
 
 DFS
 
 
 
 
  
 
 
 
 
 
  
 
 
 
 
 
  
 
 ESR1_low_EERES_low vs ESR1_low_EERES_high 0.5115840757349344
ESR1_low_EERES_low vs ESR1_high_EERES_low 0.6360337395293292
ESR1_low_EERES_low vs ESR1_high_EERES_high 0.845122748789334
ESR1_low_EERES_high vs ESR1_high_EERES_low 0.8687878461045657
ESR1_low_EERES_high vs ESR1_high_EERES_high 0.3602871089654599
ESR1_high_EERES_low vs ESR1_high_EERES_high 0.2119404851538137
                     ESR1_low_EERES_low ESR1_low_EERES_high  \
ESR1_low_EERES_low                  NaN            0.511584   
ESR1_low_EERES_high                 NaN                 NaN   
ESR1_high_EERES_low                 NaN                 NaN   
ESR1_high_EERES_high                NaN                 NaN   

                     ESR1_high_EERES_low ESR1_high_EERES_high  
ESR1_low_EERES_low              0.636034             0.845123  
ESR1_low_EERES_high             0.868788             0.360287  
ESR1_high_EERES_low                  NaN              0.21194  
ESR1_high_EERES_high                 NaN                  NaN  
 
 
 
 
  
 
 
 
 
 
  
 
 DSS
 
 
 
 
  
 
 
 
 
 
  
 
 
 
 
 
  
 
 ESR1_low_EERES_low vs ESR1_low_EERES_high 0.4521656813501922
ESR1_low_EERES_low vs ESR1_high_EERES_low 0.5474808401254836
ESR1_low_EERES_low vs ESR1_high_EERES_high 0.09185060039891405
ESR1_low_EERES_high vs ESR1_high_EERES_low 0.27698616050758074
ESR1_low_EERES_high vs ESR1_high_EERES_high 0.06577751779867913
ESR1_high_EERES_low vs ESR1_high_EERES_high 0.4410291749926585
                     ESR1_low_EERES_low ESR1_low_EERES_high  \
ESR1_low_EERES_low                  NaN            0.452166   
ESR1_low_EERES_high                 NaN                 NaN   
ESR1_high_EERES_low                 NaN                 NaN   
ESR1_high_EERES_high                NaN                 NaN   

                     ESR1_high_EERES_low ESR1_high_EERES_high  
ESR1_low_EERES_low              0.547481             0.091851  
ESR1_low_EERES_high             0.276986             0.065778  
ESR1_high_EERES_low                  NaN             0.441029  
ESR1_high_EERES_high                 NaN                  NaN  
 
 
 
 
  
 
 
 
 
 
  
 
 0.25000000000000006
PFS
 
 
 
 
  
 
 
 
 
 
  
 
 
 
 
 
  
 
 ESR1_low_EERES_low vs ESR1_low_EERES_high 0.6041400499518633
ESR1_low_EERES_low vs ESR1_high_EERES_low 0.2530709963729081
ESR1_low_EERES_low vs ESR1_high_EERES_high 0.5388154992686951
ESR1_low_EERES_high vs ESR1_high_EERES_low 0.07473720820823107
ESR1_low_EERES_high vs ESR1_high_EERES_high 0.9638363189382196
ESR1_high_EERES_low vs ESR1_high_EERES_high 0.05357855418752254
                     ESR1_low_EERES_low ESR1_low_EERES_high  \
ESR1_low_EERES_low                  NaN             0.60414   
ESR1_low_EERES_high                 NaN                 NaN   
ESR1_high_EERES_low                 NaN                 NaN   
ESR1_high_EERES_high                NaN                 NaN   

                     ESR1_high_EERES_low ESR1_high_EERES_high  
ESR1_low_EERES_low              0.253071             0.538815  
ESR1_low_EERES_high             0.074737             0.963836  
ESR1_high_EERES_low                  NaN             0.053579  
ESR1_high_EERES_high                 NaN                  NaN  
 
 
 
 
  
 
 
 
 
 
  
 
 OS
 
 
 
 
  
 
 
 
 
 
  
 
 
 
 
 
  
 
 ESR1_low_EERES_low vs ESR1_low_EERES_high 0.8593440941343111
ESR1_low_EERES_low vs ESR1_high_EERES_low 0.4695966951919802
ESR1_low_EERES_low vs ESR1_high_EERES_high 0.29912542754635785
ESR1_low_EERES_high vs ESR1_high_EERES_low 0.44615071586061594
ESR1_low_EERES_high vs ESR1_high_EERES_high 0.43433680564822175
ESR1_high_EERES_low vs ESR1_high_EERES_high 0.8299744001895408
                     ESR1_low_EERES_low ESR1_low_EERES_high  \
ESR1_low_EERES_low                  NaN            0.859344   
ESR1_low_EERES_high                 NaN                 NaN   
ESR1_high_EERES_low                 NaN                 NaN   
ESR1_high_EERES_high                NaN                 NaN   

                     ESR1_high_EERES_low ESR1_high_EERES_high  
ESR1_low_EERES_low              0.469597             0.299125  
ESR1_low_EERES_high             0.446151             0.434337  
ESR1_high_EERES_low                  NaN             0.829974  
ESR1_high_EERES_high                 NaN                  NaN  
 
 
 
 
  
 
 
 
 
 
  
 
 DFS
 
 
 
 
  
 
 
 
 
 
  
 
 
 
 
 
  
 
 ESR1_low_EERES_low vs ESR1_low_EERES_high 0.9000380010432019
ESR1_low_EERES_low vs ESR1_high_EERES_low 0.22513362854847863
ESR1_low_EERES_low vs ESR1_high_EERES_high 0.3591452367266007
ESR1_low_EERES_high vs ESR1_high_EERES_low 0.10179338844810748
ESR1_low_EERES_high vs ESR1_high_EERES_high 0.5756259566357591
ESR1_high_EERES_low vs ESR1_high_EERES_high 0.004854088871972836
                     ESR1_low_EERES_low ESR1_low_EERES_high  \
ESR1_low_EERES_low                  NaN            0.900038   
ESR1_low_EERES_high                 NaN                 NaN   
ESR1_high_EERES_low                 NaN                 NaN   
ESR1_high_EERES_high                NaN                 NaN   

                     ESR1_high_EERES_low ESR1_high_EERES_high  
ESR1_low_EERES_low              0.225134             0.359145  
ESR1_low_EERES_high             0.101793             0.575626  
ESR1_high_EERES_low                  NaN             0.004854  
ESR1_high_EERES_high                 NaN                  NaN  
 
 
 
 
  
 
 
 
 
 
  
 
 DSS
 
 
 
 
  
 
 
 
 
 
  
 
 
 
 
 
  
 
 ESR1_low_EERES_low vs ESR1_low_EERES_high 0.6028765819021226
ESR1_low_EERES_low vs ESR1_high_EERES_low 0.08151187580045727
ESR1_low_EERES_low vs ESR1_high_EERES_high 0.07139574250467706
ESR1_low_EERES_high vs ESR1_high_EERES_low 0.2924638597495853
ESR1_low_EERES_high vs ESR1_high_EERES_high 0.31006434881733286
ESR1_high_EERES_low vs ESR1_high_EERES_high 0.6543242908847227
                     ESR1_low_EERES_low ESR1_low_EERES_high  \
ESR1_low_EERES_low                  NaN            0.602877   
ESR1_low_EERES_high                 NaN                 NaN   
ESR1_high_EERES_low                 NaN                 NaN   
ESR1_high_EERES_high                NaN                 NaN   

                     ESR1_high_EERES_low ESR1_high_EERES_high  
ESR1_low_EERES_low              0.081512             0.071396  
ESR1_low_EERES_high             0.292464             0.310064  
ESR1_high_EERES_low                  NaN             0.654324  
ESR1_high_EERES_high                 NaN                  NaN  
 
 
 
 
  
 
 
 
 
 
  
 
 0.30000000000000004
PFS
 
 
 
 
  
 
 
 
 
 
  
 
 
 
 
 
  
 
 ESR1_low_EERES_low vs ESR1_low_EERES_high 0.5058132013021313
ESR1_low_EERES_low vs ESR1_high_EERES_low 0.19549844746287565
ESR1_low_EERES_low vs ESR1_high_EERES_high 0.7199192796832914
ESR1_low_EERES_high vs ESR1_high_EERES_low 0.044598516666416135
ESR1_low_EERES_high vs ESR1_high_EERES_high 0.8380343349471735
ESR1_high_EERES_low vs ESR1_high_EERES_high 0.04392883302561642
                     ESR1_low_EERES_low ESR1_low_EERES_high  \
ESR1_low_EERES_low                  NaN            0.505813   
ESR1_low_EERES_high                 NaN                 NaN   
ESR1_high_EERES_low                 NaN                 NaN   
ESR1_high_EERES_high                NaN                 NaN   

                     ESR1_high_EERES_low ESR1_high_EERES_high  
ESR1_low_EERES_low              0.195498             0.719919  
ESR1_low_EERES_high             0.044599             0.838034  
ESR1_high_EERES_low                  NaN             0.043929  
ESR1_high_EERES_high                 NaN                  NaN  
 
 
 
 
  
 
 
 
 
 
  
 
 OS
 
 
 
 
  
 
 
 
 
 
  
 
 
 
 
 
  
 
 ESR1_low_EERES_low vs ESR1_low_EERES_high 0.9380965829360217
ESR1_low_EERES_low vs ESR1_high_EERES_low 0.8872985288092695
ESR1_low_EERES_low vs ESR1_high_EERES_high 0.26030395757036157
ESR1_low_EERES_high vs ESR1_high_EERES_low 0.9852233370671866
ESR1_low_EERES_high vs ESR1_high_EERES_high 0.5688175897052216
ESR1_high_EERES_low vs ESR1_high_EERES_high 0.5418884834069067
                     ESR1_low_EERES_low ESR1_low_EERES_high  \
ESR1_low_EERES_low                  NaN            0.938097   
ESR1_low_EERES_high                 NaN                 NaN   
ESR1_high_EERES_low                 NaN                 NaN   
ESR1_high_EERES_high                NaN                 NaN   

                     ESR1_high_EERES_low ESR1_high_EERES_high  
ESR1_low_EERES_low              0.887299             0.260304  
ESR1_low_EERES_high             0.985223             0.568818  
ESR1_high_EERES_low                  NaN             0.541888  
ESR1_high_EERES_high                 NaN                  NaN  
 
 
 
 
  
 
 
 
 
 
  
 
 DFS
 
 
 
 
  
 
 
 
 
 
  
 
 
 
 
 
  
 
 ESR1_low_EERES_low vs ESR1_low_EERES_high 0.41232725147562477
ESR1_low_EERES_low vs ESR1_high_EERES_low 0.26721420831984144
ESR1_low_EERES_low vs ESR1_high_EERES_high 0.41539298110155
ESR1_low_EERES_high vs ESR1_high_EERES_low 0.042862607793559104
ESR1_low_EERES_high vs ESR1_high_EERES_high 0.7340103569255207
ESR1_high_EERES_low vs ESR1_high_EERES_high 0.024698975981733953
                     ESR1_low_EERES_low ESR1_low_EERES_high  \
ESR1_low_EERES_low                  NaN            0.412327   
ESR1_low_EERES_high                 NaN                 NaN   
ESR1_high_EERES_low                 NaN                 NaN   
ESR1_high_EERES_high                NaN                 NaN   

                     ESR1_high_EERES_low ESR1_high_EERES_high  
ESR1_low_EERES_low              0.267214             0.415393  
ESR1_low_EERES_high             0.042863              0.73401  
ESR1_high_EERES_low                  NaN             0.024699  
ESR1_high_EERES_high                 NaN                  NaN  
 
 
 
 
  
 
 
 
 
 
  
 
 DSS
 
 
 
 
  
 
 
 
 
 
  
 
 
 
 
 
  
 
 ESR1_low_EERES_low vs ESR1_low_EERES_high 0.28021312518838576
ESR1_low_EERES_low vs ESR1_high_EERES_low 0.05668713718928911
ESR1_low_EERES_low vs ESR1_high_EERES_high 0.05470216674870091
ESR1_low_EERES_high vs ESR1_high_EERES_low 0.563194608222823
ESR1_low_EERES_high vs ESR1_high_EERES_high 0.627749952586823
ESR1_high_EERES_low vs ESR1_high_EERES_high 0.6755485120255311
                     ESR1_low_EERES_low ESR1_low_EERES_high  \
ESR1_low_EERES_low                  NaN            0.280213   
ESR1_low_EERES_high                 NaN                 NaN   
ESR1_high_EERES_low                 NaN                 NaN   
ESR1_high_EERES_high                NaN                 NaN   

                     ESR1_high_EERES_low ESR1_high_EERES_high  
ESR1_low_EERES_low              0.056687             0.054702  
ESR1_low_EERES_high             0.563195              0.62775  
ESR1_high_EERES_low                  NaN             0.675549  
ESR1_high_EERES_high                 NaN                  NaN  
 
 
 
 
  
 
 
 
 
 
  
 
 0.3500000000000001
PFS
 
 
 
 
  
 
 
 
 
 
  
 
 
 
 
 
  
 
 ESR1_low_EERES_low vs ESR1_low_EERES_high 0.4798360422639487
ESR1_low_EERES_low vs ESR1_high_EERES_low 0.11370601547195149
ESR1_low_EERES_low vs ESR1_high_EERES_high 0.6574717665362904
ESR1_low_EERES_high vs ESR1_high_EERES_low 0.03773374576566702
ESR1_low_EERES_high vs ESR1_high_EERES_high 0.8209793487394946
ESR1_high_EERES_low vs ESR1_high_EERES_high 0.02496886626110304
                     ESR1_low_EERES_low ESR1_low_EERES_high  \
ESR1_low_EERES_low                  NaN            0.479836   
ESR1_low_EERES_high                 NaN                 NaN   
ESR1_high_EERES_low                 NaN                 NaN   
ESR1_high_EERES_high                NaN                 NaN   

                     ESR1_high_EERES_low ESR1_high_EERES_high  
ESR1_low_EERES_low              0.113706             0.657472  
ESR1_low_EERES_high             0.037734             0.820979  
ESR1_high_EERES_low                  NaN             0.024969  
ESR1_high_EERES_high                 NaN                  NaN  
 
 
 
 
  
 
 
 
 
 
  
 
 OS
 
 
 
 
  
 
 
 
 
 
  
 
 
 
 
 
  
 
 ESR1_low_EERES_low vs ESR1_low_EERES_high 0.8171097507821032
ESR1_low_EERES_low vs ESR1_high_EERES_low 0.4038273599144595
ESR1_low_EERES_low vs ESR1_high_EERES_high 0.2636054567905919
ESR1_low_EERES_high vs ESR1_high_EERES_low 0.3930261472869109
ESR1_low_EERES_high vs ESR1_high_EERES_high 0.6468840357266319
ESR1_high_EERES_low vs ESR1_high_EERES_high 0.8021157055063309
                     ESR1_low_EERES_low ESR1_low_EERES_high  \
ESR1_low_EERES_low                  NaN             0.81711   
ESR1_low_EERES_high                 NaN                 NaN   
ESR1_high_EERES_low                 NaN                 NaN   
ESR1_high_EERES_high                NaN                 NaN   

                     ESR1_high_EERES_low ESR1_high_EERES_high  
ESR1_low_EERES_low              0.403827             0.263605  
ESR1_low_EERES_high             0.393026             0.646884  
ESR1_high_EERES_low                  NaN             0.802116  
ESR1_high_EERES_high                 NaN                  NaN  
 
 
 
 
  
 
 
 
 
 
  
 
 DFS
 
 
 
 
  
 
 
 
 
 
  
 
 
 
 
 
  
 
 ESR1_low_EERES_low vs ESR1_low_EERES_high 0.2773766277419356
ESR1_low_EERES_low vs ESR1_high_EERES_low 0.139812347350742
ESR1_low_EERES_low vs ESR1_high_EERES_high 0.3582962416541551
ESR1_low_EERES_high vs ESR1_high_EERES_low 0.021142154737416503
ESR1_low_EERES_high vs ESR1_high_EERES_high 0.6250399176419952
ESR1_high_EERES_low vs ESR1_high_EERES_high 0.008748059383925337
                     ESR1_low_EERES_low ESR1_low_EERES_high  \
ESR1_low_EERES_low                  NaN            0.277377   
ESR1_low_EERES_high                 NaN                 NaN   
ESR1_high_EERES_low                 NaN                 NaN   
ESR1_high_EERES_high                NaN                 NaN   

                     ESR1_high_EERES_low ESR1_high_EERES_high  
ESR1_low_EERES_low              0.139812             0.358296  
ESR1_low_EERES_high             0.021142              0.62504  
ESR1_high_EERES_low                  NaN             0.008748  
ESR1_high_EERES_high                 NaN                  NaN  
 
 
 
 
  
 
 
 
 
 
  
 
 DSS
 
 
 
 
  
 
 
 
 
 
  
 
 
 
 
 
  
 
 ESR1_low_EERES_low vs ESR1_low_EERES_high 0.10663165998822288
ESR1_low_EERES_low vs ESR1_high_EERES_low 0.010672575317710353
ESR1_low_EERES_low vs ESR1_high_EERES_high 0.039141447538481644
ESR1_low_EERES_high vs ESR1_high_EERES_low 0.333738636398704
ESR1_low_EERES_high vs ESR1_high_EERES_high 0.9937658841319399
ESR1_high_EERES_low vs ESR1_high_EERES_high 0.3762049645769673
                     ESR1_low_EERES_low ESR1_low_EERES_high  \
ESR1_low_EERES_low                  NaN            0.106632   
ESR1_low_EERES_high                 NaN                 NaN   
ESR1_high_EERES_low                 NaN                 NaN   
ESR1_high_EERES_high                NaN                 NaN   

                     ESR1_high_EERES_low ESR1_high_EERES_high  
ESR1_low_EERES_low              0.010673             0.039141  
ESR1_low_EERES_high             0.333739             0.993766  
ESR1_high_EERES_low                  NaN             0.376205  
ESR1_high_EERES_high                 NaN                  NaN  
 
 
 
 
  
 
 
 
 
 
  
 
 0.40000000000000013
PFS
 
 
 
 
  
 
 
 
 
 
  
 
 
 
 
 
  
 
 ESR1_low_EERES_low vs ESR1_low_EERES_high 0.9873769158980736
ESR1_low_EERES_low vs ESR1_high_EERES_low 0.03319634964525207
ESR1_low_EERES_low vs ESR1_high_EERES_high 0.9035441783339548
ESR1_low_EERES_high vs ESR1_high_EERES_low 0.06041617680404591
ESR1_low_EERES_high vs ESR1_high_EERES_high 0.7474555738875708
ESR1_high_EERES_low vs ESR1_high_EERES_high 0.015240539757677763
                     ESR1_low_EERES_low ESR1_low_EERES_high  \
ESR1_low_EERES_low                  NaN            0.987377   
ESR1_low_EERES_high                 NaN                 NaN   
ESR1_high_EERES_low                 NaN                 NaN   
ESR1_high_EERES_high                NaN                 NaN   

                     ESR1_high_EERES_low ESR1_high_EERES_high  
ESR1_low_EERES_low              0.033196             0.903544  
ESR1_low_EERES_high             0.060416             0.747456  
ESR1_high_EERES_low                  NaN             0.015241  
ESR1_high_EERES_high                 NaN                  NaN  
 
 
 
 
  
 
 
 
 
 
  
 
 OS
 
 
 
 
  
 
 
 
 
 
  
 
 
 
 
 
  
 
 ESR1_low_EERES_low vs ESR1_low_EERES_high 0.9099969188108601
ESR1_low_EERES_low vs ESR1_high_EERES_low 0.15294386624066017
ESR1_low_EERES_low vs ESR1_high_EERES_high 0.09255246245786314
ESR1_low_EERES_high vs ESR1_high_EERES_low 0.165289150136204
ESR1_low_EERES_high vs ESR1_high_EERES_high 0.21937328231803468
ESR1_high_EERES_low vs ESR1_high_EERES_high 0.7514538813901733
                     ESR1_low_EERES_low ESR1_low_EERES_high  \
ESR1_low_EERES_low                  NaN            0.909997   
ESR1_low_EERES_high                 NaN                 NaN   
ESR1_high_EERES_low                 NaN                 NaN   
ESR1_high_EERES_high                NaN                 NaN   

                     ESR1_high_EERES_low ESR1_high_EERES_high  
ESR1_low_EERES_low              0.152944             0.092552  
ESR1_low_EERES_high             0.165289             0.219373  
ESR1_high_EERES_low                  NaN             0.751454  
ESR1_high_EERES_high                 NaN                  NaN  
 
 
 
 
  
 
 
 
 
 
  
 
 DFS
 
 
 
 
  
 
 
 
 
 
  
 
 
 
 
 
  
 
 ESR1_low_EERES_low vs ESR1_low_EERES_high 0.8241361876221089
ESR1_low_EERES_low vs ESR1_high_EERES_low 0.06616032967377358
ESR1_low_EERES_low vs ESR1_high_EERES_high 0.48136870727622494
ESR1_low_EERES_high vs ESR1_high_EERES_low 0.06184207333993803
ESR1_low_EERES_high vs ESR1_high_EERES_high 0.6956698098670374
ESR1_high_EERES_low vs ESR1_high_EERES_high 0.0037427082266420475
                     ESR1_low_EERES_low ESR1_low_EERES_high  \
ESR1_low_EERES_low                  NaN            0.824136   
ESR1_low_EERES_high                 NaN                 NaN   
ESR1_high_EERES_low                 NaN                 NaN   
ESR1_high_EERES_high                NaN                 NaN   

                     ESR1_high_EERES_low ESR1_high_EERES_high  
ESR1_low_EERES_low               0.06616             0.481369  
ESR1_low_EERES_high             0.061842              0.69567  
ESR1_high_EERES_low                  NaN             0.003743  
ESR1_high_EERES_high                 NaN                  NaN  
 
 
 
 
  
 
 
 
 
 
  
 
 DSS
 
 
 
 
  
 
 
 
 
 
  
 
 
 
 
 
  
 
 ESR1_low_EERES_low vs ESR1_low_EERES_high 0.12365433396065431
ESR1_low_EERES_low vs ESR1_high_EERES_low 0.0032554693974250534
ESR1_low_EERES_low vs ESR1_high_EERES_high 0.013931631344921752
ESR1_low_EERES_high vs ESR1_high_EERES_low 0.16332985504177772
ESR1_low_EERES_high vs ESR1_high_EERES_high 0.5320083839142234
ESR1_high_EERES_low vs ESR1_high_EERES_high 0.5095807807399126
                     ESR1_low_EERES_low ESR1_low_EERES_high  \
ESR1_low_EERES_low                  NaN            0.123654   
ESR1_low_EERES_high                 NaN                 NaN   
ESR1_high_EERES_low                 NaN                 NaN   
ESR1_high_EERES_high                NaN                 NaN   

                     ESR1_high_EERES_low ESR1_high_EERES_high  
ESR1_low_EERES_low              0.003255             0.013932  
ESR1_low_EERES_high              0.16333             0.532008  
ESR1_high_EERES_low                  NaN             0.509581  
ESR1_high_EERES_high                 NaN                  NaN  
 
 
 
 
  
 
 
 
 
 
  
 
 0.45000000000000007
PFS
 
 
 
 
  
 
 
 
 
 
  
 
 
 
 
 
  
 
 ESR1_low_EERES_low vs ESR1_low_EERES_high 0.982427382552995
ESR1_low_EERES_low vs ESR1_high_EERES_low 0.08041735054888723
ESR1_low_EERES_low vs ESR1_high_EERES_high 0.6314572009729043
ESR1_low_EERES_high vs ESR1_high_EERES_low 0.1429808692907533
ESR1_low_EERES_high vs ESR1_high_EERES_high 0.4370418803994094
ESR1_high_EERES_low vs ESR1_high_EERES_high 0.03693243268051407
                     ESR1_low_EERES_low ESR1_low_EERES_high  \
ESR1_low_EERES_low                  NaN            0.982427   
ESR1_low_EERES_high                 NaN                 NaN   
ESR1_high_EERES_low                 NaN                 NaN   
ESR1_high_EERES_high                NaN                 NaN   

                     ESR1_high_EERES_low ESR1_high_EERES_high  
ESR1_low_EERES_low              0.080417             0.631457  
ESR1_low_EERES_high             0.142981             0.437042  
ESR1_high_EERES_low                  NaN             0.036932  
ESR1_high_EERES_high                 NaN                  NaN  
 
 
 
 
  
 
 
 
 
 
  
 
 OS
 
 
 
 
  
 
 
 
 
 
  
 
 
 
 
 
  
 
 ESR1_low_EERES_low vs ESR1_low_EERES_high 0.7252573668206016
ESR1_low_EERES_low vs ESR1_high_EERES_low 0.25029395616333006
ESR1_low_EERES_low vs ESR1_high_EERES_high 0.24509500502611406
ESR1_low_EERES_high vs ESR1_high_EERES_low 0.21564283368854534
ESR1_low_EERES_high vs ESR1_high_EERES_high 0.3042654447199684
ESR1_high_EERES_low vs ESR1_high_EERES_high 0.6562459972469991
                     ESR1_low_EERES_low ESR1_low_EERES_high  \
ESR1_low_EERES_low                  NaN            0.725257   
ESR1_low_EERES_high                 NaN                 NaN   
ESR1_high_EERES_low                 NaN                 NaN   
ESR1_high_EERES_high                NaN                 NaN   

                     ESR1_high_EERES_low ESR1_high_EERES_high  
ESR1_low_EERES_low              0.250294             0.245095  
ESR1_low_EERES_high             0.215643             0.304265  
ESR1_high_EERES_low                  NaN             0.656246  
ESR1_high_EERES_high                 NaN                  NaN  
 
 
 
 
  
 
 
 
 
 
  
 
 DFS
 
 
 
 
  
 
 
 
 
 
  
 
 
 
 
 
  
 
 ESR1_low_EERES_low vs ESR1_low_EERES_high 0.7359247658028732
ESR1_low_EERES_low vs ESR1_high_EERES_low 0.1909820075089022
ESR1_low_EERES_low vs ESR1_high_EERES_high 0.16961770051186148
ESR1_low_EERES_high vs ESR1_high_EERES_low 0.15204909586444373
ESR1_low_EERES_high vs ESR1_high_EERES_high 0.3504359089844693
ESR1_high_EERES_low vs ESR1_high_EERES_high 0.007947491281592795
                     ESR1_low_EERES_low ESR1_low_EERES_high  \
ESR1_low_EERES_low                  NaN            0.735925   
ESR1_low_EERES_high                 NaN                 NaN   
ESR1_high_EERES_low                 NaN                 NaN   
ESR1_high_EERES_high                NaN                 NaN   

                     ESR1_high_EERES_low ESR1_high_EERES_high  
ESR1_low_EERES_low              0.190982             0.169618  
ESR1_low_EERES_high             0.152049             0.350436  
ESR1_high_EERES_low                  NaN             0.007947  
ESR1_high_EERES_high                 NaN                  NaN  
 
 
 
 
  
 
 
 
 
 
  
 
 DSS
 
 
 
 
  
 
 
 
 
 
  
 
 
 
 
 
  
 
 ESR1_low_EERES_low vs ESR1_low_EERES_high 0.4875711681913767
ESR1_low_EERES_low vs ESR1_high_EERES_low 0.09186688445153474
ESR1_low_EERES_low vs ESR1_high_EERES_high 0.07060409169879438
ESR1_low_EERES_high vs ESR1_high_EERES_low 0.32276949957104184
ESR1_low_EERES_high vs ESR1_high_EERES_high 0.5328935947512027
ESR1_high_EERES_low vs ESR1_high_EERES_high 0.8439934074982907
                     ESR1_low_EERES_low ESR1_low_EERES_high  \
ESR1_low_EERES_low                  NaN            0.487571   
ESR1_low_EERES_high                 NaN                 NaN   
ESR1_high_EERES_low                 NaN                 NaN   
ESR1_high_EERES_high                NaN                 NaN   

                     ESR1_high_EERES_low ESR1_high_EERES_high  
ESR1_low_EERES_low              0.091867             0.070604  
ESR1_low_EERES_high             0.322769             0.532894  
ESR1_high_EERES_low                  NaN             0.843993  
ESR1_high_EERES_high                 NaN                  NaN  
 
 
 
 
  
 
 
 
 
 
  
 
 0.5000000000000001
PFS
 
 
 
 
  
 
 
 
 
 
  
 
 
 
 
 
  
 
 ESR1_low_EERES_low vs ESR1_low_EERES_high 0.45839751253583605
ESR1_low_EERES_low vs ESR1_high_EERES_low 0.07411214962475982
ESR1_low_EERES_low vs ESR1_high_EERES_high 0.3769704516985296
ESR1_low_EERES_high vs ESR1_high_EERES_low 0.28265464628928877
ESR1_low_EERES_high vs ESR1_high_EERES_high 0.075463204142372
ESR1_high_EERES_low vs ESR1_high_EERES_high 0.021088955803457276
                     ESR1_low_EERES_low ESR1_low_EERES_high  \
ESR1_low_EERES_low                  NaN            0.458398   
ESR1_low_EERES_high                 NaN                 NaN   
ESR1_high_EERES_low                 NaN                 NaN   
ESR1_high_EERES_high                NaN                 NaN   

                     ESR1_high_EERES_low ESR1_high_EERES_high  
ESR1_low_EERES_low              0.074112              0.37697  
ESR1_low_EERES_high             0.282655             0.075463  
ESR1_high_EERES_low                  NaN             0.021089  
ESR1_high_EERES_high                 NaN                  NaN  
 
 
 
 
  
 
 
 
 
 
  
 
 OS
 
 
 
 
  
 
 
 
 
 
  
 
 
 
 
 
  
 
 ESR1_low_EERES_low vs ESR1_low_EERES_high 0.9997852566302716
ESR1_low_EERES_low vs ESR1_high_EERES_low 0.10789518563010361
ESR1_low_EERES_low vs ESR1_high_EERES_high 0.3419563718060396
ESR1_low_EERES_high vs ESR1_high_EERES_low 0.10099202822608018
ESR1_low_EERES_high vs ESR1_high_EERES_high 0.5365758040099686
ESR1_high_EERES_low vs ESR1_high_EERES_high 0.3277452671141016
                     ESR1_low_EERES_low ESR1_low_EERES_high  \
ESR1_low_EERES_low                  NaN            0.999785   
ESR1_low_EERES_high                 NaN                 NaN   
ESR1_high_EERES_low                 NaN                 NaN   
ESR1_high_EERES_high                NaN                 NaN   

                     ESR1_high_EERES_low ESR1_high_EERES_high  
ESR1_low_EERES_low              0.107895             0.341956  
ESR1_low_EERES_high             0.100992             0.536576  
ESR1_high_EERES_low                  NaN             0.327745  
ESR1_high_EERES_high                 NaN                  NaN  
 
 
 
 
  
 
 
 
 
 
  
 
 DFS
 
 
 
 
  
 
 
 
 
 
  
 
 
 
 
 
  
 
 ESR1_low_EERES_low vs ESR1_low_EERES_high 0.7001545046038424
ESR1_low_EERES_low vs ESR1_high_EERES_low 0.2830286719697306
ESR1_low_EERES_low vs ESR1_high_EERES_high 0.08114127326813025
ESR1_low_EERES_high vs ESR1_high_EERES_low 0.4461225701313132
ESR1_low_EERES_high vs ESR1_high_EERES_high 0.04539577130181184
ESR1_high_EERES_low vs ESR1_high_EERES_high 0.012183988636368303
                     ESR1_low_EERES_low ESR1_low_EERES_high  \
ESR1_low_EERES_low                  NaN            0.700155   
ESR1_low_EERES_high                 NaN                 NaN   
ESR1_high_EERES_low                 NaN                 NaN   
ESR1_high_EERES_high                NaN                 NaN   

                     ESR1_high_EERES_low ESR1_high_EERES_high  
ESR1_low_EERES_low              0.283029             0.081141  
ESR1_low_EERES_high             0.446123             0.045396  
ESR1_high_EERES_low                  NaN             0.012184  
ESR1_high_EERES_high                 NaN                  NaN  
 
 
 
 
  
 
 
 
 
 
  
 
 DSS
 
 
 
 
  
 
 
 
 
 
  
 
 
 
 
 
  
 
 ESR1_low_EERES_low vs ESR1_low_EERES_high 0.0860082565346043
ESR1_low_EERES_low vs ESR1_high_EERES_low 0.016525849681837193
ESR1_low_EERES_low vs ESR1_high_EERES_high 0.18246972445820414
ESR1_low_EERES_high vs ESR1_high_EERES_low 0.35030658649065693
ESR1_low_EERES_high vs ESR1_high_EERES_high 0.4992959569561384
ESR1_high_EERES_low vs ESR1_high_EERES_high 0.299840571766389
                     ESR1_low_EERES_low ESR1_low_EERES_high  \
ESR1_low_EERES_low                  NaN            0.086008   
ESR1_low_EERES_high                 NaN                 NaN   
ESR1_high_EERES_low                 NaN                 NaN   
ESR1_high_EERES_high                NaN                 NaN   

                     ESR1_high_EERES_low ESR1_high_EERES_high  
ESR1_low_EERES_low              0.016526              0.18247  
ESR1_low_EERES_high             0.350307             0.499296  
ESR1_high_EERES_low                  NaN             0.299841  
ESR1_high_EERES_high                 NaN                  NaN  
 
 
 
 
  
 
 
 
 
 
  
 
 0.5500000000000002
PFS
 
 
 
 
  
 
 
 
 
 
  
 
 
 
 
 
  
 
 ESR1_low_EERES_low vs ESR1_low_EERES_high 0.7623079871639259
ESR1_low_EERES_low vs ESR1_high_EERES_low 0.17961486648753947
ESR1_low_EERES_low vs ESR1_high_EERES_high 0.09648824996481367
ESR1_low_EERES_high vs ESR1_high_EERES_low 0.327478355152512
ESR1_low_EERES_high vs ESR1_high_EERES_high 0.06645029844699525
ESR1_high_EERES_low vs ESR1_high_EERES_high 0.0074432420661959915
                     ESR1_low_EERES_low ESR1_low_EERES_high  \
ESR1_low_EERES_low                  NaN            0.762308   
ESR1_low_EERES_high                 NaN                 NaN   
ESR1_high_EERES_low                 NaN                 NaN   
ESR1_high_EERES_high                NaN                 NaN   

                     ESR1_high_EERES_low ESR1_high_EERES_high  
ESR1_low_EERES_low              0.179615             0.096488  
ESR1_low_EERES_high             0.327478              0.06645  
ESR1_high_EERES_low                  NaN             0.007443  
ESR1_high_EERES_high                 NaN                  NaN  
 
 
 
 
  
 
 
 
 
 
  
 
 OS
 
 
 
 
  
 
 
 
 
 
  
 
 
 
 
 
  
 
 ESR1_low_EERES_low vs ESR1_low_EERES_high 0.41749331647720145
ESR1_low_EERES_low vs ESR1_high_EERES_low 0.004678567907211327
ESR1_low_EERES_low vs ESR1_high_EERES_high 0.8182014017155887
ESR1_low_EERES_high vs ESR1_high_EERES_low 0.0773601511861495
ESR1_low_EERES_high vs ESR1_high_EERES_high 0.7330358437291215
ESR1_high_EERES_low vs ESR1_high_EERES_high 0.027006980818053923
                     ESR1_low_EERES_low ESR1_low_EERES_high  \
ESR1_low_EERES_low                  NaN            0.417493   
ESR1_low_EERES_high                 NaN                 NaN   
ESR1_high_EERES_low                 NaN                 NaN   
ESR1_high_EERES_high                NaN                 NaN   

                     ESR1_high_EERES_low ESR1_high_EERES_high  
ESR1_low_EERES_low              0.004679             0.818201  
ESR1_low_EERES_high              0.07736             0.733036  
ESR1_high_EERES_low                  NaN             0.027007  
ESR1_high_EERES_high                 NaN                  NaN  
 
 
 
 
  
 
 
 
 
 
  
 
 DFS
 
 
 
 
  
 
 
 
 
 
  
 
 
 
 
 
  
 
 ESR1_low_EERES_low vs ESR1_low_EERES_high 0.5325781490043979
ESR1_low_EERES_low vs ESR1_high_EERES_low 0.7816417522941512
ESR1_low_EERES_low vs ESR1_high_EERES_high 0.08118422997510498
ESR1_low_EERES_high vs ESR1_high_EERES_low 0.332373800806317
ESR1_low_EERES_high vs ESR1_high_EERES_high 0.216776957541912
ESR1_high_EERES_low vs ESR1_high_EERES_high 0.06629657055857258
                     ESR1_low_EERES_low ESR1_low_EERES_high  \
ESR1_low_EERES_low                  NaN            0.532578   
ESR1_low_EERES_high                 NaN                 NaN   
ESR1_high_EERES_low                 NaN                 NaN   
ESR1_high_EERES_high                NaN                 NaN   

                     ESR1_high_EERES_low ESR1_high_EERES_high  
ESR1_low_EERES_low              0.781642             0.081184  
ESR1_low_EERES_high             0.332374             0.216777  
ESR1_high_EERES_low                  NaN             0.066297  
ESR1_high_EERES_high                 NaN                  NaN  
 
 
 
 
  
 
 
 
 
 
  
 
 DSS
 
 
 
 
  
 
 
 
 
 
  
 
 
 
 
 
  
 
 ESR1_low_EERES_low vs ESR1_low_EERES_high 0.009328380815078537
ESR1_low_EERES_low vs ESR1_high_EERES_low 0.0020579715864256455
ESR1_low_EERES_low vs ESR1_high_EERES_high 0.652255085238989
ESR1_low_EERES_high vs ESR1_high_EERES_low 0.6254816559143137
ESR1_low_EERES_high vs ESR1_high_EERES_high 0.12233392646195887
ESR1_high_EERES_low vs ESR1_high_EERES_high 0.03592742946626071
                     ESR1_low_EERES_low ESR1_low_EERES_high  \
ESR1_low_EERES_low                  NaN            0.009328   
ESR1_low_EERES_high                 NaN                 NaN   
ESR1_high_EERES_low                 NaN                 NaN   
ESR1_high_EERES_high                NaN                 NaN   

                     ESR1_high_EERES_low ESR1_high_EERES_high  
ESR1_low_EERES_low              0.002058             0.652255  
ESR1_low_EERES_high             0.625482             0.122334  
ESR1_high_EERES_low                  NaN             0.035927  
ESR1_high_EERES_high                 NaN                  NaN  
 
 
 
 
  
 
 
 
 
 
  
 
 0.6000000000000002
PFS
 
 
 
 
  
 
 
 
 
 
  
 
 
 
 
 
  
 
 ESR1_low_EERES_low vs ESR1_low_EERES_high 0.6959349378096316
ESR1_low_EERES_low vs ESR1_high_EERES_low 0.09057494926841844
ESR1_low_EERES_low vs ESR1_high_EERES_high 0.03493102340453924
ESR1_low_EERES_high vs ESR1_high_EERES_low 0.28761874215146355
ESR1_low_EERES_high vs ESR1_high_EERES_high 0.01324351804114535
ESR1_high_EERES_low vs ESR1_high_EERES_high 0.0012720396101921284
                     ESR1_low_EERES_low ESR1_low_EERES_high  \
ESR1_low_EERES_low                  NaN            0.695935   
ESR1_low_EERES_high                 NaN                 NaN   
ESR1_high_EERES_low                 NaN                 NaN   
ESR1_high_EERES_high                NaN                 NaN   

                     ESR1_high_EERES_low ESR1_high_EERES_high  
ESR1_low_EERES_low              0.090575             0.034931  
ESR1_low_EERES_high             0.287619             0.013244  
ESR1_high_EERES_low                  NaN             0.001272  
ESR1_high_EERES_high                 NaN                  NaN  
 
 
 
 
  
 
 
 
 
 
  
 
 OS
 
 
 
 
  
 
 
 
 
 
  
 
 
 
 
 
  
 
 ESR1_low_EERES_low vs ESR1_low_EERES_high 0.24466975894055207
ESR1_low_EERES_low vs ESR1_high_EERES_low 0.0053376595793309285
ESR1_low_EERES_low vs ESR1_high_EERES_high 0.7733551847895382
ESR1_low_EERES_high vs ESR1_high_EERES_low 0.17294460978943757
ESR1_low_EERES_high vs ESR1_high_EERES_high 0.2839526376566176
ESR1_high_EERES_low vs ESR1_high_EERES_high 0.013476510585546482
                     ESR1_low_EERES_low ESR1_low_EERES_high  \
ESR1_low_EERES_low                  NaN             0.24467   
ESR1_low_EERES_high                 NaN                 NaN   
ESR1_high_EERES_low                 NaN                 NaN   
ESR1_high_EERES_high                NaN                 NaN   

                     ESR1_high_EERES_low ESR1_high_EERES_high  
ESR1_low_EERES_low              0.005338             0.773355  
ESR1_low_EERES_high             0.172945             0.283953  
ESR1_high_EERES_low                  NaN             0.013477  
ESR1_high_EERES_high                 NaN                  NaN  
 
 
 
 
  
 
 
 
 
 
  
 
 DFS
 
 
 
 
  
 
 
 
 
 
  
 
 
 
 
 
  
 
 ESR1_low_EERES_low vs ESR1_low_EERES_high 0.8839831745605427
ESR1_low_EERES_low vs ESR1_high_EERES_low 0.42715336510697444
ESR1_low_EERES_low vs ESR1_high_EERES_high 0.02324005266469036
ESR1_low_EERES_high vs ESR1_high_EERES_low 0.33407798319418397
ESR1_low_EERES_high vs ESR1_high_EERES_high 0.024540917833679836
ESR1_high_EERES_low vs ESR1_high_EERES_high 0.006855157567469663
                     ESR1_low_EERES_low ESR1_low_EERES_high  \
ESR1_low_EERES_low                  NaN            0.883983   
ESR1_low_EERES_high                 NaN                 NaN   
ESR1_high_EERES_low                 NaN                 NaN   
ESR1_high_EERES_high                NaN                 NaN   

                     ESR1_high_EERES_low ESR1_high_EERES_high  
ESR1_low_EERES_low              0.427153              0.02324  
ESR1_low_EERES_high             0.334078             0.024541  
ESR1_high_EERES_low                  NaN             0.006855  
ESR1_high_EERES_high                 NaN                  NaN  
 
 
 
 
  
 
 
 
 
 
  
 
 DSS
 
 
 
 
  
 
 
 
 
 
  
 
 
 
 
 
  
 
 ESR1_low_EERES_low vs ESR1_low_EERES_high 0.013792847978511769
ESR1_low_EERES_low vs ESR1_high_EERES_low 0.004801316368027246
ESR1_low_EERES_low vs ESR1_high_EERES_high 0.7934501656042929
ESR1_low_EERES_high vs ESR1_high_EERES_low 0.7029476150899023
ESR1_low_EERES_high vs ESR1_high_EERES_high 0.06009259772091245
ESR1_high_EERES_low vs ESR1_high_EERES_high 0.02168994650010052
                     ESR1_low_EERES_low ESR1_low_EERES_high  \
ESR1_low_EERES_low                  NaN            0.013793   
ESR1_low_EERES_high                 NaN                 NaN   
ESR1_high_EERES_low                 NaN                 NaN   
ESR1_high_EERES_high                NaN                 NaN   

                     ESR1_high_EERES_low ESR1_high_EERES_high  
ESR1_low_EERES_low              0.004801              0.79345  
ESR1_low_EERES_high             0.702948             0.060093  
ESR1_high_EERES_low                  NaN              0.02169  
ESR1_high_EERES_high                 NaN                  NaN  
 
 
 
 
  
 
 
 
 
 
  
 
 0.6500000000000001
PFS
 
 
 
 
  
 
 
 
 
 
  
 
 
 
 
 
  
 
 ESR1_low_EERES_low vs ESR1_low_EERES_high 0.5368876878465606
ESR1_low_EERES_low vs ESR1_high_EERES_low 0.057146857297087114
ESR1_low_EERES_low vs ESR1_high_EERES_high 0.0315722762261594
ESR1_low_EERES_high vs ESR1_high_EERES_low 0.23158262422018566
ESR1_low_EERES_high vs ESR1_high_EERES_high 0.006002694865759897
ESR1_high_EERES_low vs ESR1_high_EERES_high 0.0010180778656796406
                     ESR1_low_EERES_low ESR1_low_EERES_high  \
ESR1_low_EERES_low                  NaN            0.536888   
ESR1_low_EERES_high                 NaN                 NaN   
ESR1_high_EERES_low                 NaN                 NaN   
ESR1_high_EERES_high                NaN                 NaN   

                     ESR1_high_EERES_low ESR1_high_EERES_high  
ESR1_low_EERES_low              0.057147             0.031572  
ESR1_low_EERES_high             0.231583             0.006003  
ESR1_high_EERES_low                  NaN             0.001018  
ESR1_high_EERES_high                 NaN                  NaN  
 
 
 
 
  
 
 
 
 
 
  
 
 OS
 
 
 
 
  
 
 
 
 
 
  
 
 
 
 
 
  
 
 ESR1_low_EERES_low vs ESR1_low_EERES_high 0.2010628123066087
ESR1_low_EERES_low vs ESR1_high_EERES_low 0.015692692519150134
ESR1_low_EERES_low vs ESR1_high_EERES_high 0.7097412919206219
ESR1_low_EERES_high vs ESR1_high_EERES_low 0.28172096354689413
ESR1_low_EERES_high vs ESR1_high_EERES_high 0.26900789034517814
ESR1_high_EERES_low vs ESR1_high_EERES_high 0.05672685138527434
                     ESR1_low_EERES_low ESR1_low_EERES_high  \
ESR1_low_EERES_low                  NaN            0.201063   
ESR1_low_EERES_high                 NaN                 NaN   
ESR1_high_EERES_low                 NaN                 NaN   
ESR1_high_EERES_high                NaN                 NaN   

                     ESR1_high_EERES_low ESR1_high_EERES_high  
ESR1_low_EERES_low              0.015693             0.709741  
ESR1_low_EERES_high             0.281721             0.269008  
ESR1_high_EERES_low                  NaN             0.056727  
ESR1_high_EERES_high                 NaN                  NaN  
 
 
 
 
  
 
 
 
 
 
  
 
 DFS
 
 
 
 
  
 
 
 
 
 
  
 
 
 
 
 
  
 
 ESR1_low_EERES_low vs ESR1_low_EERES_high 0.9807412957743201
ESR1_low_EERES_low vs ESR1_high_EERES_low 0.2956203112513096
ESR1_low_EERES_low vs ESR1_high_EERES_high 0.05389266596807388
ESR1_low_EERES_high vs ESR1_high_EERES_low 0.2952124313167216
ESR1_low_EERES_high vs ESR1_high_EERES_high 0.04010865202237087
ESR1_high_EERES_low vs ESR1_high_EERES_high 0.011689564885294188
                     ESR1_low_EERES_low ESR1_low_EERES_high  \
ESR1_low_EERES_low                  NaN            0.980741   
ESR1_low_EERES_high                 NaN                 NaN   
ESR1_high_EERES_low                 NaN                 NaN   
ESR1_high_EERES_high                NaN                 NaN   

                     ESR1_high_EERES_low ESR1_high_EERES_high  
ESR1_low_EERES_low               0.29562             0.053893  
ESR1_low_EERES_high             0.295212             0.040109  
ESR1_high_EERES_low                  NaN              0.01169  
ESR1_high_EERES_high                 NaN                  NaN  
 
 
 
 
  
 
 
 
 
 
  
 
 DSS
 
 
 
 
  
 
 
 
 
 
  
 
 
 
 
 
  
 
 ESR1_low_EERES_low vs ESR1_low_EERES_high 0.02009822953406045
ESR1_low_EERES_low vs ESR1_high_EERES_low 0.010451626179313573
ESR1_low_EERES_low vs ESR1_high_EERES_high 0.28143026870034427
ESR1_low_EERES_high vs ESR1_high_EERES_low 0.6570884614872321
ESR1_low_EERES_high vs ESR1_high_EERES_high 0.024712990043976382
ESR1_high_EERES_low vs ESR1_high_EERES_high 0.019547263881759785
                     ESR1_low_EERES_low ESR1_low_EERES_high  \
ESR1_low_EERES_low                  NaN            0.020098   
ESR1_low_EERES_high                 NaN                 NaN   
ESR1_high_EERES_low                 NaN                 NaN   
ESR1_high_EERES_high                NaN                 NaN   

                     ESR1_high_EERES_low ESR1_high_EERES_high  
ESR1_low_EERES_low              0.010452              0.28143  
ESR1_low_EERES_high             0.657088             0.024713  
ESR1_high_EERES_low                  NaN             0.019547  
ESR1_high_EERES_high                 NaN                  NaN  
 
 
 
 
  
 
 
 
 
 
  
 
 0.7000000000000002
PFS
 
 
 
 
  
 
 
 
 
 
  
 
 
 
 
 
  
 
 ESR1_low_EERES_low vs ESR1_low_EERES_high 0.8206095737512977
ESR1_low_EERES_low vs ESR1_high_EERES_low 0.27028718352601794
ESR1_low_EERES_low vs ESR1_high_EERES_high 0.05933685185992164
ESR1_low_EERES_high vs ESR1_high_EERES_low 0.2853714451597228
ESR1_low_EERES_high vs ESR1_high_EERES_high 0.022887114195411208
ESR1_high_EERES_low vs ESR1_high_EERES_high 0.012195557349301998
                     ESR1_low_EERES_low ESR1_low_EERES_high  \
ESR1_low_EERES_low                  NaN             0.82061   
ESR1_low_EERES_high                 NaN                 NaN   
ESR1_high_EERES_low                 NaN                 NaN   
ESR1_high_EERES_high                NaN                 NaN   

                     ESR1_high_EERES_low ESR1_high_EERES_high  
ESR1_low_EERES_low              0.270287             0.059337  
ESR1_low_EERES_high             0.285371             0.022887  
ESR1_high_EERES_low                  NaN             0.012196  
ESR1_high_EERES_high                 NaN                  NaN  
 
 
 
 
  
 
 
 
 
 
  
 
 OS
 
 
 
 
  
 
 
 
 
 
  
 
 
 
 
 
  
 
 ESR1_low_EERES_low vs ESR1_low_EERES_high 0.784656423950342
ESR1_low_EERES_low vs ESR1_high_EERES_low 0.09098154206462047
ESR1_low_EERES_low vs ESR1_high_EERES_high 0.4462480354597165
ESR1_low_EERES_high vs ESR1_high_EERES_low 0.22224223243066665
ESR1_low_EERES_high vs ESR1_high_EERES_high 0.3602731732558776
ESR1_high_EERES_low vs ESR1_high_EERES_high 0.16506710117708986
                     ESR1_low_EERES_low ESR1_low_EERES_high  \
ESR1_low_EERES_low                  NaN            0.784656   
ESR1_low_EERES_high                 NaN                 NaN   
ESR1_high_EERES_low                 NaN                 NaN   
ESR1_high_EERES_high                NaN                 NaN   

                     ESR1_high_EERES_low ESR1_high_EERES_high  
ESR1_low_EERES_low              0.090982             0.446248  
ESR1_low_EERES_high             0.222242             0.360273  
ESR1_high_EERES_low                  NaN             0.165067  
ESR1_high_EERES_high                 NaN                  NaN  
 
 
 
 
  
 
 
 
 
 
  
 
 DFS
 
 
 
 
  
 
 
 
 
 
  
 
 
 
 
 
  
 
 ESR1_low_EERES_low vs ESR1_low_EERES_high 0.6109911461829366
ESR1_low_EERES_low vs ESR1_high_EERES_low 0.8483443890001743
ESR1_low_EERES_low vs ESR1_high_EERES_high 0.07522696921037074
ESR1_low_EERES_high vs ESR1_high_EERES_low 0.5571704577315346
ESR1_low_EERES_high vs ESR1_high_EERES_high 0.1164362863976808
ESR1_high_EERES_low vs ESR1_high_EERES_high 0.056490151824570616
                     ESR1_low_EERES_low ESR1_low_EERES_high  \
ESR1_low_EERES_low                  NaN            0.610991   
ESR1_low_EERES_high                 NaN                 NaN   
ESR1_high_EERES_low                 NaN                 NaN   
ESR1_high_EERES_high                NaN                 NaN   

                     ESR1_high_EERES_low ESR1_high_EERES_high  
ESR1_low_EERES_low              0.848344             0.075227  
ESR1_low_EERES_high              0.55717             0.116436  
ESR1_high_EERES_low                  NaN              0.05649  
ESR1_high_EERES_high                 NaN                  NaN  
 
 
 
 
  
 
 
 
 
 
  
 
 DSS
 
 
 
 
  
 
 
 
 
 
  
 
 
 
 
 
  
 
 ESR1_low_EERES_low vs ESR1_low_EERES_high 0.13057845648158886
ESR1_low_EERES_low vs ESR1_high_EERES_low 0.22079567199324665
ESR1_low_EERES_low vs ESR1_high_EERES_high 0.27414226609845604
ESR1_low_EERES_high vs ESR1_high_EERES_low 0.8854423680963981
ESR1_low_EERES_high vs ESR1_high_EERES_high 0.04691004523208199
ESR1_high_EERES_low vs ESR1_high_EERES_high 0.13057892246564853
                     ESR1_low_EERES_low ESR1_low_EERES_high  \
ESR1_low_EERES_low                  NaN            0.130578   
ESR1_low_EERES_high                 NaN                 NaN   
ESR1_high_EERES_low                 NaN                 NaN   
ESR1_high_EERES_high                NaN                 NaN   

                     ESR1_high_EERES_low ESR1_high_EERES_high  
ESR1_low_EERES_low              0.220796             0.274142  
ESR1_low_EERES_high             0.885442              0.04691  
ESR1_high_EERES_low                  NaN             0.130579  
ESR1_high_EERES_high                 NaN                  NaN  
 
 
 
 
  
 
 
 
 
 
  
 
 0.7500000000000002
PFS
 
 
 
 
  
 
 
 
 
 
  
 
 
 
 
 
  
 
 ESR1_low_EERES_low vs ESR1_low_EERES_high 0.6672591904151143
ESR1_low_EERES_low vs ESR1_high_EERES_low 0.39354255348368594
ESR1_low_EERES_low vs ESR1_high_EERES_high 0.1025233225555485
ESR1_low_EERES_high vs ESR1_high_EERES_low 0.181541901888896
ESR1_low_EERES_high vs ESR1_high_EERES_high 0.09828210228873673
ESR1_high_EERES_low vs ESR1_high_EERES_high 0.04395971391328831
                     ESR1_low_EERES_low ESR1_low_EERES_high  \
ESR1_low_EERES_low                  NaN            0.667259   
ESR1_low_EERES_high                 NaN                 NaN   
ESR1_high_EERES_low                 NaN                 NaN   
ESR1_high_EERES_high                NaN                 NaN   

                     ESR1_high_EERES_low ESR1_high_EERES_high  
ESR1_low_EERES_low              0.393543             0.102523  
ESR1_low_EERES_high             0.181542             0.098282  
ESR1_high_EERES_low                  NaN              0.04396  
ESR1_high_EERES_high                 NaN                  NaN  
 
 
 
 
  
 
 
 
 
 
  
 
 OS
 
 
 
 
  
 
 
 
 
 
  
 
 
 
 
 
  
 
 ESR1_low_EERES_low vs ESR1_low_EERES_high 0.31898091112087645
ESR1_low_EERES_low vs ESR1_high_EERES_low 0.13079069572894378
ESR1_low_EERES_low vs ESR1_high_EERES_high 0.10607532962813669
ESR1_low_EERES_high vs ESR1_high_EERES_low 0.024923148237325887
ESR1_low_EERES_high vs ESR1_high_EERES_high 0.18349227114101313
ESR1_high_EERES_low vs ESR1_high_EERES_high 0.03624526973838203
                     ESR1_low_EERES_low ESR1_low_EERES_high  \
ESR1_low_EERES_low                  NaN            0.318981   
ESR1_low_EERES_high                 NaN                 NaN   
ESR1_high_EERES_low                 NaN                 NaN   
ESR1_high_EERES_high                NaN                 NaN   

                     ESR1_high_EERES_low ESR1_high_EERES_high  
ESR1_low_EERES_low              0.130791             0.106075  
ESR1_low_EERES_high             0.024923             0.183492  
ESR1_high_EERES_low                  NaN             0.036245  
ESR1_high_EERES_high                 NaN                  NaN  
 
 
 
 
  
 
 
 
 
 
  
 
 DFS
 
 
 
 
  
 
 
 
 
 
  
 
 
 
 
 
  
 
 ESR1_low_EERES_low vs ESR1_low_EERES_high 0.40046118771396466
ESR1_low_EERES_low vs ESR1_high_EERES_low 0.9022696275831421
ESR1_low_EERES_low vs ESR1_high_EERES_high 0.14855442161136204
ESR1_low_EERES_high vs ESR1_high_EERES_low 0.576062582762795
ESR1_low_EERES_high vs ESR1_high_EERES_high 0.23945506571028233
ESR1_high_EERES_low vs ESR1_high_EERES_high 0.16791619933495094
                     ESR1_low_EERES_low ESR1_low_EERES_high  \
ESR1_low_EERES_low                  NaN            0.400461   
ESR1_low_EERES_high                 NaN                 NaN   
ESR1_high_EERES_low                 NaN                 NaN   
ESR1_high_EERES_high                NaN                 NaN   

                     ESR1_high_EERES_low ESR1_high_EERES_high  
ESR1_low_EERES_low               0.90227             0.148554  
ESR1_low_EERES_high             0.576063             0.239455  
ESR1_high_EERES_low                  NaN             0.167916  
ESR1_high_EERES_high                 NaN                  NaN  
 
 
 
 
  
 
 
 
 
 
  
 
 DSS
 
 
 
 
  
 
 
 
 
 
  
 
 
 
 
 
  
 
 ESR1_low_EERES_low vs ESR1_low_EERES_high 0.7676973776686257
ESR1_low_EERES_low vs ESR1_high_EERES_low 0.6525881119047849
ESR1_low_EERES_low vs ESR1_high_EERES_high 0.2873091612136399
ESR1_low_EERES_high vs ESR1_high_EERES_low 0.6895233288312068
ESR1_low_EERES_high vs ESR1_high_EERES_high 0.18451778024473142
ESR1_high_EERES_low vs ESR1_high_EERES_high 0.23209292479855362
                     ESR1_low_EERES_low ESR1_low_EERES_high  \
ESR1_low_EERES_low                  NaN            0.767697   
ESR1_low_EERES_high                 NaN                 NaN   
ESR1_high_EERES_low                 NaN                 NaN   
ESR1_high_EERES_high                NaN                 NaN   

                     ESR1_high_EERES_low ESR1_high_EERES_high  
ESR1_low_EERES_low              0.652588             0.287309  
ESR1_low_EERES_high             0.689523             0.184518  
ESR1_high_EERES_low                  NaN             0.232093  
ESR1_high_EERES_high                 NaN                  NaN  
 
 
 
 
  
 
 
 
 
 
  
 
 0.8000000000000002
PFS
 
 
 
 
  
 
 
 
 
 
  
 
 
 
 
 
  
 
 ESR1_low_EERES_low vs ESR1_low_EERES_high 0.8187971900684419
ESR1_low_EERES_low vs ESR1_high_EERES_low 0.8199606200817952
ESR1_low_EERES_low vs ESR1_high_EERES_high 0.12941587324193007
ESR1_low_EERES_high vs ESR1_high_EERES_low 0.44456380783828064
ESR1_low_EERES_high vs ESR1_high_EERES_high 0.114305437635495
ESR1_high_EERES_low vs ESR1_high_EERES_high 0.09767001442122476
                     ESR1_low_EERES_low ESR1_low_EERES_high  \
ESR1_low_EERES_low                  NaN            0.818797   
ESR1_low_EERES_high                 NaN                 NaN   
ESR1_high_EERES_low                 NaN                 NaN   
ESR1_high_EERES_high                NaN                 NaN   

                     ESR1_high_EERES_low ESR1_high_EERES_high  
ESR1_low_EERES_low              0.819961             0.129416  
ESR1_low_EERES_high             0.444564             0.114305  
ESR1_high_EERES_low                  NaN              0.09767  
ESR1_high_EERES_high                 NaN                  NaN  
 
 
 
 
  
 
 
 
 
 
  
 
 OS
 
 
 
 
  
 
 
 
 
 
  
 
 
 
 
 
  
 
 ESR1_low_EERES_low vs ESR1_low_EERES_high 0.42177466137839437
ESR1_low_EERES_low vs ESR1_high_EERES_low 0.13657526015914012
ESR1_low_EERES_low vs ESR1_high_EERES_high 0.13548597416435734
ESR1_low_EERES_high vs ESR1_high_EERES_low 0.03912416313617889
ESR1_low_EERES_high vs ESR1_high_EERES_high 0.18302732675681152
ESR1_high_EERES_low vs ESR1_high_EERES_high 0.040512113875311534
                     ESR1_low_EERES_low ESR1_low_EERES_high  \
ESR1_low_EERES_low                  NaN            0.421775   
ESR1_low_EERES_high                 NaN                 NaN   
ESR1_high_EERES_low                 NaN                 NaN   
ESR1_high_EERES_high                NaN                 NaN   

                     ESR1_high_EERES_low ESR1_high_EERES_high  
ESR1_low_EERES_low              0.136575             0.135486  
ESR1_low_EERES_high             0.039124             0.183027  
ESR1_high_EERES_low                  NaN             0.040512  
ESR1_high_EERES_high                 NaN                  NaN  
 
 
 
 
  
 
 
 
 
 
  
 
 DFS
 
 
 
 
  
 
 
 
 
 
  
 
 
 
 
 
  
 
 ESR1_low_EERES_low vs ESR1_low_EERES_high 0.5020004837486047
ESR1_low_EERES_low vs ESR1_high_EERES_low 0.2996334313582004
ESR1_low_EERES_low vs ESR1_high_EERES_high 0.17502287486628693
ESR1_low_EERES_high vs ESR1_high_EERES_low 0.7544470094259244
ESR1_low_EERES_high vs ESR1_high_EERES_high 0.2647392078480632
ESR1_high_EERES_low vs ESR1_high_EERES_high 0.39802471950693796
                     ESR1_low_EERES_low ESR1_low_EERES_high  \
ESR1_low_EERES_low                  NaN               0.502   
ESR1_low_EERES_high                 NaN                 NaN   
ESR1_high_EERES_low                 NaN                 NaN   
ESR1_high_EERES_high                NaN                 NaN   

                     ESR1_high_EERES_low ESR1_high_EERES_high  
ESR1_low_EERES_low              0.299633             0.175023  
ESR1_low_EERES_high             0.754447             0.264739  
ESR1_high_EERES_low                  NaN             0.398025  
ESR1_high_EERES_high                 NaN                  NaN  
 
 
 
 
  
 
 
 
 
 
  
 
 DSS
 
 
 
 
  
 
 
 
 
 
  
 
 
 
 
 
  
 
 ESR1_low_EERES_low vs ESR1_low_EERES_high 0.5882976889485216
ESR1_low_EERES_low vs ESR1_high_EERES_low 0.46888334939557363
ESR1_low_EERES_low vs ESR1_high_EERES_high 0.3266830382118065
ESR1_low_EERES_high vs ESR1_high_EERES_low 0.6648905724759219
ESR1_low_EERES_high vs ESR1_high_EERES_high 0.18613733602385588
ESR1_high_EERES_low vs ESR1_high_EERES_high 0.22525290636064965
                     ESR1_low_EERES_low ESR1_low_EERES_high  \
ESR1_low_EERES_low                  NaN            0.588298   
ESR1_low_EERES_high                 NaN                 NaN   
ESR1_high_EERES_low                 NaN                 NaN   
ESR1_high_EERES_high                NaN                 NaN   

                     ESR1_high_EERES_low ESR1_high_EERES_high  
ESR1_low_EERES_low              0.468883             0.326683  
ESR1_low_EERES_high             0.664891             0.186137  
ESR1_high_EERES_low                  NaN             0.225253  
ESR1_high_EERES_high                 NaN                  NaN  
 
 
 
 
  
 
 
 
 
 
  
 
 0.8500000000000002
PFS
 
 
 
 
  
 
 
 
 
 
  
 
 
 
 
 
  
 
 ESR1_low_EERES_low vs ESR1_low_EERES_high 0.11294029603681321
ESR1_low_EERES_low vs ESR1_high_EERES_low 0.9506643695471886
ESR1_low_EERES_low vs ESR1_high_EERES_high 0.17041162828681325
ESR1_low_EERES_high vs ESR1_high_EERES_low 0.09780675079570984
ESR1_low_EERES_high vs ESR1_high_EERES_high 0.5727023597783104
ESR1_high_EERES_low vs ESR1_high_EERES_high 0.21861507806463215
                     ESR1_low_EERES_low ESR1_low_EERES_high  \
ESR1_low_EERES_low                  NaN             0.11294   
ESR1_low_EERES_high                 NaN                 NaN   
ESR1_high_EERES_low                 NaN                 NaN   
ESR1_high_EERES_high                NaN                 NaN   

                     ESR1_high_EERES_low ESR1_high_EERES_high  
ESR1_low_EERES_low              0.950664             0.170412  
ESR1_low_EERES_high             0.097807             0.572702  
ESR1_high_EERES_low                  NaN             0.218615  
ESR1_high_EERES_high                 NaN                  NaN  
 
 
 
 
  
 
 
 
 
 
  
 
 OS
 
 
 
 
  
 
 
 
 
 
  
 
 
 
 
 
  
 
 ESR1_low_EERES_low vs ESR1_low_EERES_high 0.05144461909813354
ESR1_low_EERES_low vs ESR1_high_EERES_low 0.4596358987245618
ESR1_low_EERES_low vs ESR1_high_EERES_high 0.1711472454608188
ESR1_low_EERES_high vs ESR1_high_EERES_low 0.04517232602038412
ESR1_low_EERES_high vs ESR1_high_EERES_high 1.0
ESR1_high_EERES_low vs ESR1_high_EERES_high 0.1424959833799518
                     ESR1_low_EERES_low ESR1_low_EERES_high  \
ESR1_low_EERES_low                  NaN            0.051445   
ESR1_low_EERES_high                 NaN                 NaN   
ESR1_high_EERES_low                 NaN                 NaN   
ESR1_high_EERES_high                NaN                 NaN   

                     ESR1_high_EERES_low ESR1_high_EERES_high  
ESR1_low_EERES_low              0.459636             0.171147  
ESR1_low_EERES_high             0.045172                  1.0  
ESR1_high_EERES_low                  NaN             0.142496  
ESR1_high_EERES_high                 NaN                  NaN  
 
 
 
 
  
 
 
 
 
 
  
 
 DFS
 
 
 
 
  
 
 
 
 
 
  
 
 
 
 
 
  
 
 ESR1_low_EERES_low vs ESR1_low_EERES_high 0.1921521612791453
ESR1_low_EERES_low vs ESR1_high_EERES_low 0.4339500891265684
ESR1_low_EERES_low vs ESR1_high_EERES_high 0.24616030463684727
ESR1_low_EERES_high vs ESR1_high_EERES_low 0.5793521176061935
ESR1_low_EERES_high vs ESR1_high_EERES_high 0.5727023597783104
ESR1_high_EERES_low vs ESR1_high_EERES_high 0.4385780260809997
                     ESR1_low_EERES_low ESR1_low_EERES_high  \
ESR1_low_EERES_low                  NaN            0.192152   
ESR1_low_EERES_high                 NaN                 NaN   
ESR1_high_EERES_low                 NaN                 NaN   
ESR1_high_EERES_high                NaN                 NaN   

                     ESR1_high_EERES_low ESR1_high_EERES_high  
ESR1_low_EERES_low               0.43395              0.24616  
ESR1_low_EERES_high             0.579352             0.572702  
ESR1_high_EERES_low                  NaN             0.438578  
ESR1_high_EERES_high                 NaN                  NaN  
 
 
 
 
  
 
 
 
 
 
  
 
 DSS
 
 
 
 
  
 
 
 
 
 
  
 
 
 
 
 
  
 
 ESR1_low_EERES_low vs ESR1_low_EERES_high 0.18961989284919784
ESR1_low_EERES_low vs ESR1_high_EERES_low 0.8939689813389983
ESR1_low_EERES_low vs ESR1_high_EERES_high 0.3288175012242954
ESR1_low_EERES_high vs ESR1_high_EERES_low 0.2206713619198432
ESR1_low_EERES_high vs ESR1_high_EERES_high 1.0
ESR1_high_EERES_low vs ESR1_high_EERES_high 0.5351434523977505
                     ESR1_low_EERES_low ESR1_low_EERES_high  \
ESR1_low_EERES_low                  NaN             0.18962   
ESR1_low_EERES_high                 NaN                 NaN   
ESR1_high_EERES_low                 NaN                 NaN   
ESR1_high_EERES_high                NaN                 NaN   

                     ESR1_high_EERES_low ESR1_high_EERES_high  
ESR1_low_EERES_low              0.893969             0.328818  
ESR1_low_EERES_high             0.220671                  1.0  
ESR1_high_EERES_low                  NaN             0.535143  
ESR1_high_EERES_high                 NaN                  NaN  
 
 
 
 
  
 
 
 
 
 
  
 
 0.9000000000000002
PFS
 
 
 
 
  
 
 
 
 
 
  
 
 
 
 
 
  
 
 ESR1_low_EERES_low vs ESR1_low_EERES_high 0.26280234640943245
ESR1_low_EERES_low vs ESR1_high_EERES_low 0.6605960880971886
ESR1_low_EERES_low vs ESR1_high_EERES_high 0.3680748365470784
ESR1_low_EERES_high vs ESR1_high_EERES_low 0.08234868877744322
ESR1_low_EERES_high vs ESR1_high_EERES_high 0.5981614526835279
ESR1_high_EERES_low vs ESR1_high_EERES_high 0.2652894025067584
                     ESR1_low_EERES_low ESR1_low_EERES_high  \
ESR1_low_EERES_low                  NaN            0.262802   
ESR1_low_EERES_high                 NaN                 NaN   
ESR1_high_EERES_low                 NaN                 NaN   
ESR1_high_EERES_high                NaN                 NaN   

                     ESR1_high_EERES_low ESR1_high_EERES_high  
ESR1_low_EERES_low              0.660596             0.368075  
ESR1_low_EERES_high             0.082349             0.598161  
ESR1_high_EERES_low                  NaN             0.265289  
ESR1_high_EERES_high                 NaN                  NaN  
 
 
 
 
  
 
 
 
 
 
  
 
 OS
 
 
 
 
  
 
 
 
 
 
  
 
 
 
 
 
  
 
 ESR1_low_EERES_low vs ESR1_low_EERES_high 0.09933504094415792
ESR1_low_EERES_low vs ESR1_high_EERES_low 0.7970987239881288
ESR1_low_EERES_low vs ESR1_high_EERES_high 0.36722400890552753
ESR1_low_EERES_high vs ESR1_high_EERES_low 0.0732404625356491
ESR1_low_EERES_high vs ESR1_high_EERES_high 1.0
ESR1_high_EERES_low vs ESR1_high_EERES_high 0.36069833626522896
                     ESR1_low_EERES_low ESR1_low_EERES_high  \
ESR1_low_EERES_low                  NaN            0.099335   
ESR1_low_EERES_high                 NaN                 NaN   
ESR1_high_EERES_low                 NaN                 NaN   
ESR1_high_EERES_high                NaN                 NaN   

                     ESR1_high_EERES_low ESR1_high_EERES_high  
ESR1_low_EERES_low              0.797099             0.367224  
ESR1_low_EERES_high              0.07324                  1.0  
ESR1_high_EERES_low                  NaN             0.360698  
ESR1_high_EERES_high                 NaN                  NaN  
 
 
 
 
  
 
 
 
 
 
  
 
 DFS
 
 
 
 
  
 
 
 
 
 
  
 
 
 
 
 
  
 
 ESR1_low_EERES_low vs ESR1_low_EERES_high 0.380392159731479
ESR1_low_EERES_low vs ESR1_high_EERES_low 0.5453895316303584
ESR1_low_EERES_low vs ESR1_high_EERES_high 0.42437026665250965
ESR1_low_EERES_high vs ESR1_high_EERES_low 0.6322502335634566
ESR1_low_EERES_high vs ESR1_high_EERES_high 0.5981614526835279
ESR1_high_EERES_low vs ESR1_high_EERES_high 0.5049850750938457
                     ESR1_low_EERES_low ESR1_low_EERES_high  \
ESR1_low_EERES_low                  NaN            0.380392   
ESR1_low_EERES_high                 NaN                 NaN   
ESR1_high_EERES_low                 NaN                 NaN   
ESR1_high_EERES_high                NaN                 NaN   

                     ESR1_high_EERES_low ESR1_high_EERES_high  
ESR1_low_EERES_low               0.54539              0.42437  
ESR1_low_EERES_high              0.63225             0.598161  
ESR1_high_EERES_low                  NaN             0.504985  
ESR1_high_EERES_high                 NaN                  NaN  
 
 
 
 
  
 
 
 
 
 
  
 
 DSS
 
 
 
 
  
 
 
 
 
 
  
 
 
 
 
 
  
 
 ESR1_low_EERES_low vs ESR1_low_EERES_high 0.27534309137800883
ESR1_low_EERES_low vs ESR1_high_EERES_low 0.9647318460017937
ESR1_low_EERES_low vs ESR1_high_EERES_high 0.5414692209150938
ESR1_low_EERES_high vs ESR1_high_EERES_low 0.20891238174069476
ESR1_low_EERES_high vs ESR1_high_EERES_high 1.0
ESR1_high_EERES_low vs ESR1_high_EERES_high 0.5741490727971621
                     ESR1_low_EERES_low ESR1_low_EERES_high  \
ESR1_low_EERES_low                  NaN            0.275343   
ESR1_low_EERES_high                 NaN                 NaN   
ESR1_high_EERES_low                 NaN                 NaN   
ESR1_high_EERES_high                NaN                 NaN   

                     ESR1_high_EERES_low ESR1_high_EERES_high  
ESR1_low_EERES_low              0.964732             0.541469  
ESR1_low_EERES_high             0.208912                  1.0  
ESR1_high_EERES_low                  NaN             0.574149  
ESR1_high_EERES_high                 NaN                  NaN  
 
 
 
 
  
 
 
 
 
 
  
 
 0.9500000000000003
PFS
 
 
 
 
  
 
 
 
 
 
  
 
 
 
 
 
  
 
 ESR1_low_EERES_low vs ESR1_low_EERES_high 0.7792047079102058
ESR1_low_EERES_low vs ESR1_high_EERES_low 0.35092780192694895
ESR1_low_EERES_low vs ESR1_high_EERES_high 0.6722737984886651
ESR1_low_EERES_high vs ESR1_high_EERES_low 0.27087438417344717
ESR1_low_EERES_high vs ESR1_high_EERES_high 0.5929800980174267
ESR1_high_EERES_low vs ESR1_high_EERES_high 0.4860994597361773
                     ESR1_low_EERES_low ESR1_low_EERES_high  \
ESR1_low_EERES_low                  NaN            0.779205   
ESR1_low_EERES_high                 NaN                 NaN   
ESR1_high_EERES_low                 NaN                 NaN   
ESR1_high_EERES_high                NaN                 NaN   

                     ESR1_high_EERES_low ESR1_high_EERES_high  
ESR1_low_EERES_low              0.350928             0.672274  
ESR1_low_EERES_high             0.270874              0.59298  
ESR1_high_EERES_low                  NaN             0.486099  
ESR1_high_EERES_high                 NaN                  NaN  
 
 
 
 
  
 
 
 
 
 
  
 
 OS
 
 
 
 
  
 
 
 
 
 
  
 
 
 
 
 
  
 
 ESR1_low_EERES_low vs ESR1_low_EERES_high 0.27958553844043826
ESR1_low_EERES_low vs ESR1_high_EERES_low 0.4896403924994549
ESR1_low_EERES_low vs ESR1_high_EERES_high 0.7023602504442037
ESR1_low_EERES_high vs ESR1_high_EERES_low 0.07356126950907078
ESR1_low_EERES_high vs ESR1_high_EERES_high 1.0
ESR1_high_EERES_low vs ESR1_high_EERES_high 0.5254280008505965
                     ESR1_low_EERES_low ESR1_low_EERES_high  \
ESR1_low_EERES_low                  NaN            0.279586   
ESR1_low_EERES_high                 NaN                 NaN   
ESR1_high_EERES_low                 NaN                 NaN   
ESR1_high_EERES_high                NaN                 NaN   

                     ESR1_high_EERES_low ESR1_high_EERES_high  
ESR1_low_EERES_low               0.48964              0.70236  
ESR1_low_EERES_high             0.073561                  1.0  
ESR1_high_EERES_low                  NaN             0.525428  
ESR1_high_EERES_high                 NaN                  NaN  
 
 
 
 
  
 
 
 
 
 
  
 
 DFS
 
 
 
 
  
 
 
 
 
 
  
 
 
 
 
 
  
 
 ESR1_low_EERES_low vs ESR1_low_EERES_high 0.9490160473143544
ESR1_low_EERES_low vs ESR1_high_EERES_low 0.3359266542698106
ESR1_low_EERES_low vs ESR1_high_EERES_high 0.70882928304802
ESR1_low_EERES_high vs ESR1_high_EERES_low 0.39802471950693796
ESR1_low_EERES_high vs ESR1_high_EERES_high 0.5929800980174267
ESR1_high_EERES_low vs ESR1_high_EERES_high 1.0
                     ESR1_low_EERES_low ESR1_low_EERES_high  \
ESR1_low_EERES_low                  NaN            0.949016   
ESR1_low_EERES_high                 NaN                 NaN   
ESR1_high_EERES_low                 NaN                 NaN   
ESR1_high_EERES_high                NaN                 NaN   

                     ESR1_high_EERES_low ESR1_high_EERES_high  
ESR1_low_EERES_low              0.335927             0.708829  
ESR1_low_EERES_high             0.398025              0.59298  
ESR1_high_EERES_low                  NaN                  1.0  
ESR1_high_EERES_high                 NaN                  NaN  
 
 
 
 
  
 
 
 
 
 
  
 
 DSS
 
 
 
 
  
 
 
 
 
 
  
 
 
 
 
 
  
 
 ESR1_low_EERES_low vs ESR1_low_EERES_high 0.49243459049686833
ESR1_low_EERES_low vs ESR1_high_EERES_low 0.4520306548830516
ESR1_low_EERES_low vs ESR1_high_EERES_high 0.7927353036331211
ESR1_low_EERES_high vs ESR1_high_EERES_low 0.20590321073206466
ESR1_low_EERES_high vs ESR1_high_EERES_high 1.0
ESR1_high_EERES_low vs ESR1_high_EERES_high 0.6547208460185769
                     ESR1_low_EERES_low ESR1_low_EERES_high  \
ESR1_low_EERES_low                  NaN            0.492435   
ESR1_low_EERES_high                 NaN                 NaN   
ESR1_high_EERES_low                 NaN                 NaN   
ESR1_high_EERES_high                NaN                 NaN   

                     ESR1_high_EERES_low ESR1_high_EERES_high  
ESR1_low_EERES_low              0.452031             0.792735  
ESR1_low_EERES_high             0.205903                  1.0  
ESR1_high_EERES_low                  NaN             0.654721  
ESR1_high_EERES_high                 NaN                  NaN  
 
 
 
 
  
 
 
 
 
 
 
  
 
 
 
 
 In [16]: 
 
 
     q  =  0.6500000000000001 
 BRCA_ESR1_Threshold   =   q_groups  [  q  ][  'BRCA_ESR1_Threshold'  ] 
 BRCA_EERES_Threshold   =   q_groups  [  q  ][  'BRCA_EERES_Threshold'  ] 

 print  (  "PFS"  ) 

 br_pfs_es   =   brca_pfs  .  join  (  brca_esr1_es_df  ,   how  =  'inner'  ) 
 br_pfs_es   =   br_pfs_es  [  ~  br_pfs_es  .  index  .  duplicated  (  keep  =  "first"  )] 
 plotting_2groups  (  br_pfs_es  ,   "PFS"  ,   BRCA_ESR1_Threshold  ,   BRCA_EERES_Threshold  ) 
 plotting_4groups  (  br_pfs_es  ,   "PFS"  ,   BRCA_ESR1_Threshold  ,   BRCA_EERES_Threshold  ) 

 print  (  "OS"  ) 

 br_os_es   =   brca_os  .  join  (  brca_esr1_es_df  ,   how  =  'inner'  ) 
 br_os_es   =   br_os_es  [  ~  br_os_es  .  index  .  duplicated  (  keep  =  "first"  )] 
 plotting_2groups  (  br_os_es  ,   "OS"  ,   BRCA_ESR1_Threshold  ,   BRCA_EERES_Threshold  ) 
 plotting_4groups  (  br_os_es  ,   "OS"  ,   BRCA_ESR1_Threshold  ,   BRCA_EERES_Threshold  ) 

 print  (  "DFS"  ) 

 br_dfs_es   =   brca_dfs  .  join  (  brca_esr1_es_df  ,   how  =  'inner'  ) 
 br_dfs_es   =   br_dfs_es  [  ~  br_dfs_es  .  index  .  duplicated  (  keep  =  "first"  )] 
 plotting_2groups  (  br_dfs_es  ,   "DFS"  ,   BRCA_ESR1_Threshold  ,   BRCA_EERES_Threshold  ) 
 plotting_4groups  (  br_dfs_es  ,   "DFS"  ,   BRCA_ESR1_Threshold  ,   BRCA_EERES_Threshold  ) 

 print  (  "DSS"  ) 

 br_dss_es   =   brca_dss  .  join  (  brca_esr1_es_df  ,   how  =  'inner'  ) 
 br_dss_es   =   br_dss_es  [  ~  br_dss_es  .  index  .  duplicated  (  keep  =  "first"  )] 
 plotting_2groups  (  br_dss_es  ,   "DSS"  ,   BRCA_ESR1_Threshold  ,   BRCA_EERES_Threshold  ) 
 plotting_4groups  (  br_dss_es  ,   "DSS"  ,   BRCA_ESR1_Threshold  ,   BRCA_EERES_Threshold  ) 
  
 
 
 
 
 
 
 
 
 
  
 
 PFS
 
 
 
 
  
 
 
 
 
 
  
 
 
 
 
 
  
 
 ESR1_low_EERES_low vs ESR1_low_EERES_high 0.5368876878465606
ESR1_low_EERES_low vs ESR1_high_EERES_low 0.057146857297087114
ESR1_low_EERES_low vs ESR1_high_EERES_high 0.0315722762261594
ESR1_low_EERES_high vs ESR1_high_EERES_low 0.23158262422018566
ESR1_low_EERES_high vs ESR1_high_EERES_high 0.006002694865759897
ESR1_high_EERES_low vs ESR1_high_EERES_high 0.0010180778656796406
                     ESR1_low_EERES_low ESR1_low_EERES_high  \
ESR1_low_EERES_low                  NaN            0.536888   
ESR1_low_EERES_high                 NaN                 NaN   
ESR1_high_EERES_low                 NaN                 NaN   
ESR1_high_EERES_high                NaN                 NaN   

                     ESR1_high_EERES_low ESR1_high_EERES_high  
ESR1_low_EERES_low              0.057147             0.031572  
ESR1_low_EERES_high             0.231583             0.006003  
ESR1_high_EERES_low                  NaN             0.001018  
ESR1_high_EERES_high                 NaN                  NaN  
 
 
 
 
  
 
 
 
 
 
  
 
 OS
 
 
 
 
  
 
 
 
 
 
  
 
 
 
 
 
  
 
 ESR1_low_EERES_low vs ESR1_low_EERES_high 0.2010628123066087
ESR1_low_EERES_low vs ESR1_high_EERES_low 0.015692692519150134
ESR1_low_EERES_low vs ESR1_high_EERES_high 0.7097412919206219
ESR1_low_EERES_high vs ESR1_high_EERES_low 0.28172096354689413
ESR1_low_EERES_high vs ESR1_high_EERES_high 0.26900789034517814
ESR1_high_EERES_low vs ESR1_high_EERES_high 0.05672685138527434
                     ESR1_low_EERES_low ESR1_low_EERES_high  \
ESR1_low_EERES_low                  NaN            0.201063   
ESR1_low_EERES_high                 NaN                 NaN   
ESR1_high_EERES_low                 NaN                 NaN   
ESR1_high_EERES_high                NaN                 NaN   

                     ESR1_high_EERES_low ESR1_high_EERES_high  
ESR1_low_EERES_low              0.015693             0.709741  
ESR1_low_EERES_high             0.281721             0.269008  
ESR1_high_EERES_low                  NaN             0.056727  
ESR1_high_EERES_high                 NaN                  NaN  
 
 
 
 
  
 
 
 
 
 
  
 
 DFS
 
 
 
 
  
 
 
 
 
 
  
 
 
 
 
 
  
 
 ESR1_low_EERES_low vs ESR1_low_EERES_high 0.9807412957743201
ESR1_low_EERES_low vs ESR1_high_EERES_low 0.2956203112513096
ESR1_low_EERES_low vs ESR1_high_EERES_high 0.05389266596807388
ESR1_low_EERES_high vs ESR1_high_EERES_low 0.2952124313167216
ESR1_low_EERES_high vs ESR1_high_EERES_high 0.04010865202237087
ESR1_high_EERES_low vs ESR1_high_EERES_high 0.011689564885294188
                     ESR1_low_EERES_low ESR1_low_EERES_high  \
ESR1_low_EERES_low                  NaN            0.980741   
ESR1_low_EERES_high                 NaN                 NaN   
ESR1_high_EERES_low                 NaN                 NaN   
ESR1_high_EERES_high                NaN                 NaN   

                     ESR1_high_EERES_low ESR1_high_EERES_high  
ESR1_low_EERES_low               0.29562             0.053893  
ESR1_low_EERES_high             0.295212             0.040109  
ESR1_high_EERES_low                  NaN              0.01169  
ESR1_high_EERES_high                 NaN                  NaN  
 
 
 
 
  
 
 
 
 
 
  
 
 DSS
 
 
 
 
  
 
 
 
 
 
  
 
 
 
 
 
  
 
 ESR1_low_EERES_low vs ESR1_low_EERES_high 0.02009822953406045
ESR1_low_EERES_low vs ESR1_high_EERES_low 0.010451626179313573
ESR1_low_EERES_low vs ESR1_high_EERES_high 0.28143026870034427
ESR1_low_EERES_high vs ESR1_high_EERES_low 0.6570884614872321
ESR1_low_EERES_high vs ESR1_high_EERES_high 0.024712990043976382
ESR1_high_EERES_low vs ESR1_high_EERES_high 0.019547263881759785
                     ESR1_low_EERES_low ESR1_low_EERES_high  \
ESR1_low_EERES_low                  NaN            0.020098   
ESR1_low_EERES_high                 NaN                 NaN   
ESR1_high_EERES_low                 NaN                 NaN   
ESR1_high_EERES_high                NaN                 NaN   

                     ESR1_high_EERES_low ESR1_high_EERES_high  
ESR1_low_EERES_low              0.010452              0.28143  
ESR1_low_EERES_high             0.657088             0.024713  
ESR1_high_EERES_low                  NaN             0.019547  
ESR1_high_EERES_high                 NaN                  NaN  
 
 
 
 
  
 
 
 
 
 
 
  
 
 
 
 
 In [17]: 
 
 
     br_clinical_gdc   =   pd  .  read_table  (  "Data/gdc/TCGA-BRCA/clinical/nationwidechildrens.org_clinical_patient_brca.txt"  ,   skiprows  =  1  ,   header  =  0  ,   index_col  =  1  )  .  iloc  [  1  :] 

 brca_stage_group  ,   brca_age_group   =   groups_clinical  (  br_clinical_gdc  ,   "pathologic_stage"  ,   "age_at_initial_pathologic_diagnosis"  ,   brca_groups  ) 
 print  (  chi2_contingency  (  brca_stage_group  .  iloc  [:,:  4  ])) 
 print  (  chi2_contingency  (  brca_age_group  )) 
  
 
 
 
 
 
 
 
 
 
  
 
 Chi2ContingencyResult(statistic=13.824113634665348, pvalue=0.1287208429801775, dof=9, expected_freq=array([[3.73831776e-01, 1.12616822e+00, 4.81308411e-01, 1.86915888e-02],
       [3.73831776e+00, 1.12616822e+01, 4.81308411e+00, 1.86915888e-01],
       [3.55140187e+00, 1.06985981e+01, 4.57242991e+00, 1.77570093e-01],
       [7.23364486e+01, 2.17913551e+02, 9.31331776e+01, 3.61682243e+00]]))
Chi2ContingencyResult(statistic=13.883684624276736, pvalue=0.1265233654569464, dof=9, expected_freq=array([[1.28440367e-01, 9.77064220e-01, 7.52293578e-01, 1.42201835e-01],
       [1.28440367e+00, 9.77064220e+00, 7.52293578e+00, 1.42201835e+00],
       [1.28440367e+00, 9.77064220e+00, 7.52293578e+00, 1.42201835e+00],
       [2.53027523e+01, 1.92481651e+02, 1.48201835e+02, 2.80137615e+01]]))
 
 
 
 
  
 
 /tmp/ipykernel_1042853/4149498380.py:251: SettingWithCopyWarning: 
A value is trying to be set on a copy of a slice from a DataFrame

See the caveats in the documentation: https://pandas.pydata.org/pandas-docs/stable/user_guide/indexing.html#returning-a-view-versus-a-copy
  ages_tmp.loc[ages_tmp[age_colname]=="[Not Available]", age_colname] = "10000"
 
 
 
 
 
  
 
 
 
 
 In [18]: 
 
 
     br_gdc_histo_group   =   br_clinical_gdc  [[  'histological_type'  ]]  .  join  (  brca_groups  ,  how  =  'inner'  )  .  groupby  ([  'group'  ,  'histological_type'  ])[[  'ESR1'  ]]  .  count  () 

 br_gdc_histo_group_1   =   br_gdc_histo_group  .  loc  [  'ESR1_low_EERES_low'  ]  .  transpose  () 
 br_gdc_histo_group_1  .  index   =   br_gdc_histo_group_1  .  index  .  rename  (  'group'  ) 
 br_gdc_histo_group_1  .  columns   =   br_gdc_histo_group_1  .  columns  .  rename  (  ''  ) 
 br_gdc_histo_group_1  .  index   =   [  'ESR1_low_EERES_low'  ] 

 br_gdc_histo_group_2   =   br_gdc_histo_group  .  loc  [  'ESR1_low_EERES_high'  ]  .  transpose  () 
 br_gdc_histo_group_2  .  index   =   br_gdc_histo_group_2  .  index  .  rename  (  'group'  ) 
 br_gdc_histo_group_2  .  columns   =   br_gdc_histo_group_2  .  columns  .  rename  (  ''  ) 
 br_gdc_histo_group_2  .  index   =   [  'ESR1_low_EERES_high'  ] 

 br_gdc_histo_group_3   =   br_gdc_histo_group  .  loc  [  'ESR1_high_EERES_low'  ]  .  transpose  () 
 br_gdc_histo_group_3  .  index   =   br_gdc_histo_group_3  .  index  .  rename  (  'group'  ) 
 br_gdc_histo_group_3  .  columns   =   br_gdc_histo_group_3  .  columns  .  rename  (  ''  ) 
 br_gdc_histo_group_3  .  index   =   [  'ESR1_high_EERES_low'  ] 

 br_gdc_histo_group_4   =   br_gdc_histo_group  .  loc  [  'ESR1_high_EERES_high'  ]  .  transpose  () 
 br_gdc_histo_group_4  .  index   =   br_gdc_histo_group_4  .  index  .  rename  (  'group'  ) 
 br_gdc_histo_group_4  .  columns   =   br_gdc_histo_group_4  .  columns  .  rename  (  ''  ) 
 br_gdc_histo_group_4  .  index   =   [  'ESR1_high_EERES_high'  ] 

 br_gdc_histo_group   =   pd  .  concat  ([  br_gdc_histo_group_1  ,   br_gdc_histo_group_2  ,   br_gdc_histo_group_3  ,   br_gdc_histo_group_4  ])  .  map  (  lambda   x  :   0   if   np  .  isnan  (  x  )   else   x  ) 
 print  (  chi2_contingency  (  br_gdc_histo_group  )) 
 br_gdc_histo_group  *  100  /  br_gdc_histo_group  .  sum  (  axis  =  1  )  .  values  .  reshape  ((  -  1  ,  1  )) 
  
 
 
 
 
 
 
 
 
 
  
 
 Chi2ContingencyResult(statistic=11.38733063684378, pvalue=0.724685166632109, dof=15, expected_freq=array([[2.61160550e+02, 9.12706422e+01, 1.80733945e+00, 1.26513761e+01,
        1.17477064e+01, 1.53623853e+01],
       [1.32568807e+01, 4.63302752e+00, 9.17431193e-02, 6.42201835e-01,
        5.96330275e-01, 7.79816514e-01],
       [1.32568807e+01, 4.63302752e+00, 9.17431193e-02, 6.42201835e-01,
        5.96330275e-01, 7.79816514e-01],
       [1.32568807e+00, 4.63302752e-01, 9.17431193e-03, 6.42201835e-02,
        5.96330275e-02, 7.79816514e-02]]))
 
 
 
 
 Out[18]: 
 
 
 
 
 
 
  
 Infiltrating Ductal Carcinoma 
 Infiltrating Lobular Carcinoma 
 Metaplastic Carcinoma 
 Mixed Histology (please specify) 
 Mucinous Carcinoma 
 Other, specify 
 
 
 
 
 ESR1_low_EERES_low 
 65.228426 
 24.619289 
 0.507614 
 3.299492 
 2.791878 
 3.553299 
 
 
 ESR1_low_EERES_high 
 70.000000 
 15.000000 
 0.000000 
 5.000000 
 5.000000 
 5.000000 
 
 
 ESR1_high_EERES_low 
 85.000000 
 0.000000 
 0.000000 
 0.000000 
 5.000000 
 10.000000 
 
 
 ESR1_high_EERES_high 
 50.000000 
 50.000000 
 0.000000 
 0.000000 
 0.000000 
 0.000000 
 
 
 
 
 
 
 
 
  
 
 
 
 
 In [19]: 
 
 
     brca_stage_group  *  100  /  brca_stage_group  .  sum  (  axis  =  1  )  .  values  .  reshape  ((  -  1  ,  1  )) 
  
 
 
 
 
 
 
 
 
 
 Out[19]: 
 
 
 
 
 
 
  
 T1 
 T2 
 T3 
 T4 
 [Discrepancy] 
 TX 
 [Not Available] 
 
 
 
 
 ESR1_high_EERES_high 
 50.000000 
 0.000000 
 50.000000 
 0.000000 
 NaN 
 NaN 
 NaN 
 
 
 ESR1_high_EERES_low 
 10.000000 
 50.000000 
 35.000000 
 5.000000 
 NaN 
 NaN 
 NaN 
 
 
 ESR1_low_EERES_high 
 25.000000 
 45.000000 
 20.000000 
 5.000000 
 5.000000 
 NaN 
 NaN 
 
 
 ESR1_low_EERES_low 
 18.274112 
 56.345178 
 23.096447 
 0.507614 
 0.507614 
 1.015228 
 0.253807 
 
 
 
 
 
 
 
 
  
 
 
 
 
 In [21]: 
 
 
     brca_age_group  *  100  /  brca_age_group  .  sum  (  axis  =  1  )  .  values  .  reshape  ((  -  1  ,  1  )) 
  
 
 
 
 
 
 
 
 
 
 Out[21]: 
 
 
 
 
 
 
  
 21-40 
 41-60 
 61-80 
 81-100 
 
 
 
 
 ESR1_high_EERES_high 
 0.000000 
 0.000000 
 100.000000 
 0.000000 
 
 
 ESR1_high_EERES_low 
 0.000000 
 25.000000 
 60.000000 
 15.000000 
 
 
 ESR1_low_EERES_high 
 0.000000 
 50.000000 
 45.000000 
 5.000000 
 
 
 ESR1_low_EERES_low 
 7.106599 
 50.253807 
 35.786802 
 6.852792 
 
 
 
 
 
 
 
 
  
 
 
 
 
 In [22]: 
 
 
     # The Spearman of ER-related genes vs EERES 
 brca_esr1_es_df   =   brca_gdc_tpm  .  transpose  ()[[  "ESR1"  ,  "ESR2"  ,  "ESRRA"  ,  "ESRRB"  ,  "ESRRG"  ,  "GPER1"  ]]  .  groupby  (  level  =  0  )  .  mean  ()  .  join  (  brca_es  ,   how  =  'inner'  ) 

 ERs_vs_EERES_table  (  brca_esr1_es_df  ) 
  
 
 
 
 
 
 
 
 
 
 Out[22]: 
 
 
 
 
 
 
  
 R 
 p 
 
 
 
 
 ESR1 
 0.641278 
 7.04e-128 
 
 
 ESR2 
 -0.218046 
 2.99e-13 
 
 
 ESRRA 
 -0.041781 
 1.67e-01 
 
 
 ESRRB 
 0.112625 
 1.88e-04 
 
 
 ESRRG 
 0.162339 
 6.60e-08 
 
 
 GPER1 
 0.273689 
 2.89e-20 
 
 
 
 
 
 
 
 
 
 
 
 
 
  
  
 METABRIC ¶   Figure 2f-i ¶  
 
 
 
  
 
 
 
 
 In [23]: 
 
 
     br_metabric_mrna   =   pd  .  read_table  (  "Data/brca_metabric/data_mrna_agilent_microarray.txt"  ,   index_col  =  0  )  .  iloc  [:,  1  :] 
 br_metabric_mrna  .  index  .  name   =   "gene_name" 
 br_metabric_mrna   =   br_metabric_mrna  .  groupby  (  level  =  0  )  .  mean  () 
 print  (  "n:"  ,  len  (  br_metabric_mrna  .  columns  )) 
  
 
 
 
 
 
 
 
 
 
  
 
 n: 1904
 
 
 
 
 
  
 
 
 
 
 In [24]: 
 
 
     br_metabric_hallmark_es   =   gsva_py2r  (  np  .  log2  (  br_metabric_mrna  +  1  ),  hallmark_gs  ) 
  
 
 
 
 
 
 
 
 
 
  
 
 Converting df
Estimating GSVA scores for 50 gene sets.
Estimating ECDFs with Gaussian kernels
  |======================================================================| 100%

 
 
 
 
 
  
 
 
 
 
 In [28]: 
 
 
     br_metabric_clinical_survival   =   pd  .  read_table  (  "Data/brca_metabric/data_clinical_patient.txt"  ,   skiprows  =  4  ,   header  =  0  ,   index_col  =  0  ) 
 br_metabric_clinical   =   pd  .  read_table  (  "Data/brca_metabric/brca_metabric_clinical_data.tsv"  ,   index_col  =  1  )[[  "ER Status"  ,   "HER2 Status"  ,   "Hormone Therapy"  ]] 
 br_metabric_hormonal_er_index   =   set  (  br_metabric_clinical  [(  br_metabric_clinical  [  "Hormone Therapy"  ]  ==  "YES"  )]  .  index  )  .  intersection  (  br_metabric_mrna  .  columns  ) 
 br_metabric_es   =   pd  .  DataFrame  (  br_metabric_hallmark_es  [  "HALLMARK_ESTROGEN_RESPONSE_EARLY"  ]  .  values  ,   index  =  br_metabric_hallmark_es  .  index  ,   columns  =  [  "EARLY"  ]) 
 br_metabric_es  .  rename  (  columns  =  {  'EARLY'  :  "EERES"  })  .  to_excel  (  "Table S2.xlsx"  ,   sheet_name  =  "METABRIC"  ) 
 br_metabric_esr1_es_df   =   br_metabric_mrna  .  transpose  ()  .  loc  [  list  (  br_metabric_hormonal_er_index  ),[  "ESR1"  ]]  .  join  (  br_metabric_es  ,   how  =  'inner'  ) 
 print  (  "n:"  ,  len  (  br_metabric_hormonal_er_index  )) 

 br_metabric_EERES_Threshold   =   br_metabric_esr1_es_df  [  "EARLY"  ]  .  quantile  (  0.65  ) 
 br_metabric_ESR1_Threshold   =   br_metabric_esr1_es_df  [  "ESR1"  ]  .  quantile  (  0.65  ) 
 print  (  br_metabric_ESR1_Threshold  ) 
 print  (  br_metabric_EERES_Threshold  ) 
 plotting_scatter_esr1_es  (  br_metabric_esr1_es_df  ,   br_metabric_ESR1_Threshold  ,   br_metabric_EERES_Threshold  ) 

 print  (  stats  .  spearmanr  (  np  .  log2  (  br_metabric_esr1_es_df  [  "ESR1"  ]  +  1  ),   br_metabric_esr1_es_df  [  "EARLY"  ])) 
 print  (  stats  .  pearsonr  (  np  .  log2  (  br_metabric_esr1_es_df  [  "ESR1"  ]  +  1  ),   br_metabric_esr1_es_df  [  "EARLY"  ])) 

 br_metabric_groups   =   grouping4  (  br_metabric_esr1_es_df  ,   br_metabric_ESR1_Threshold  ,   br_metabric_EERES_Threshold  ) 
  
 
 
 
 
 
 
 
 
 
  
 
 n: 1174
11.132513727
0.15338301706278723
 
 
 
 
  
 
 
 
 
 
  
 
 SignificanceResult(statistic=0.4274871958216788, pvalue=2.378334466592854e-53)
PearsonRResult(statistic=0.5753535687958771, pvalue=1.9694227883792115e-104)
 
 
 
 
 
  
 
 
 
 
 In [29]: 
 
 
     br_metabric_rfs   =   br_metabric_clinical_survival  [[  "RFS_STATUS"  ,   "RFS_MONTHS"  ]] 
 br_metabric_rfs  [  "RFS_STATUS"  ]   =   [  str  (  s  )[  0  ]   if   str  (  s  )[  0  ]   !=   "n"   else   np  .  nan   for   s   in   br_metabric_rfs  [  "RFS_STATUS"  ]] 
 br_metabric_rfs   =   br_metabric_rfs  .  dropna  () 
 br_metabric_os   =   br_metabric_clinical_survival  [[  "OS_STATUS"  ,   "OS_MONTHS"  ]] 
 br_metabric_os  [  "OS_STATUS"  ]   =   [  str  (  s  )[  0  ]   for   s   in   br_metabric_os  [  "OS_STATUS"  ]] 
 br_metabric_os   =   br_metabric_os  .  dropna  () 

 # br_metabric_esr1_es_os = br_metabric_os.join(br_metabric_esr1_es_df, how='inner') 
 # plotting_2groups(br_metabric_esr1_es_os, "OS", br_metabric_ESR1_Threshold, br_metabric_EERES_Threshold) 
 # plotting_4groups(br_metabric_esr1_es_os, "OS", br_metabric_ESR1_Threshold, br_metabric_EERES_Threshold) 

 br_metabric_esr1_es_rfs   =   br_metabric_rfs  .  join  (  br_metabric_esr1_es_df  ,   how  =  'inner'  ) 
 plotting_2groups  (  br_metabric_esr1_es_rfs  ,   "RFS"  ,   br_metabric_ESR1_Threshold  ,   br_metabric_EERES_Threshold  ) 
 plotting_4groups  (  br_metabric_esr1_es_rfs  ,   "RFS"  ,   br_metabric_ESR1_Threshold  ,   br_metabric_EERES_Threshold  ) 
  
 
 
 
 
 
 
 
 
 
  
 
 /tmp/ipykernel_1042853/4203505307.py:2: SettingWithCopyWarning: 
A value is trying to be set on a copy of a slice from a DataFrame.
Try using .loc[row_indexer,col_indexer] = value instead

See the caveats in the documentation: https://pandas.pydata.org/pandas-docs/stable/user_guide/indexing.html#returning-a-view-versus-a-copy
  br_metabric_rfs["RFS_STATUS"] = [str(s)[0] if str(s)[0] != "n" else np.nan for s in br_metabric_rfs["RFS_STATUS"]]
/tmp/ipykernel_1042853/4203505307.py:5: SettingWithCopyWarning: 
A value is trying to be set on a copy of a slice from a DataFrame.
Try using .loc[row_indexer,col_indexer] = value instead

See the caveats in the documentation: https://pandas.pydata.org/pandas-docs/stable/user_guide/indexing.html#returning-a-view-versus-a-copy
  br_metabric_os["OS_STATUS"] = [str(s)[0] for s in br_metabric_os["OS_STATUS"]]
 
 
 
 
  
 
 
 
 
 
  
 
 
 
 
 
  
 
 ESR1_low_EERES_low vs ESR1_low_EERES_high 0.036359895073894845
ESR1_low_EERES_low vs ESR1_high_EERES_low 0.1965837436290443
ESR1_low_EERES_low vs ESR1_high_EERES_high 0.0007778738276053565
ESR1_low_EERES_high vs ESR1_high_EERES_low 0.4487430284158661
ESR1_low_EERES_high vs ESR1_high_EERES_high 0.15632356713275672
ESR1_high_EERES_low vs ESR1_high_EERES_high 0.0418002535004614
                     ESR1_low_EERES_low ESR1_low_EERES_high  \
ESR1_low_EERES_low                  NaN             0.03636   
ESR1_low_EERES_high                 NaN                 NaN   
ESR1_high_EERES_low                 NaN                 NaN   
ESR1_high_EERES_high                NaN                 NaN   

                     ESR1_high_EERES_low ESR1_high_EERES_high  
ESR1_low_EERES_low              0.196584             0.000778  
ESR1_low_EERES_high             0.448743             0.156324  
ESR1_high_EERES_low                  NaN               0.0418  
ESR1_high_EERES_high                 NaN                  NaN  
 
 
 
 
  
 
 
 
 
 
 
 
 
 
 
 
  
  
 Figure 4 ¶  
 
 
 
  
 
 
 
 
 In [30]: 
 
 
     brca_deseq2_res   =   deseq2_py2r  (  brca_gtex_gdc_gc  ,   brca_gtex_gdc_pheno_df  ,   "~type"  ,   [  "type"  ,  "Cancer"  ,  "Normal"  ]) 
 ov_deseq2_res   =   deseq2_py2r  (  ov_gtex_gdc_gc  ,   ov_gtex_gdc_pheno_df  ,   "~type"  ,   [  "type"  ,  "Cancer"  ,  "Normal"  ]) 
 ucec_deseq2_res   =   deseq2_py2r  (  ucec_gtex_gdc_gc  ,   ucec_gtex_gdc_pheno_df  ,   "~type"  ,   [  "type"  ,  "Cancer"  ,  "Normal"  ]) 
 cesc_deseq2_res   =   deseq2_py2r  (  cesc_gtex_gdc_gc  ,   cesc_gtex_gdc_pheno_df  ,   "~type"  ,   [  "type"  ,  "Cancer"  ,  "Normal"  ]) 
  
 
 
 
 
 
 
 
 
 
  
 
 Converting df
 
 
 
 
  
 
 R[write to console]: estimating size factors

R[write to console]: estimating dispersions

R[write to console]: gene-wise dispersion estimates

R[write to console]: mean-dispersion relationship

R[write to console]: final dispersion estimates

R[write to console]: fitting model and testing

R[write to console]: -- replacing outliers and refitting for 5674 genes
-- DESeq argument 'minReplicatesForReplace' = 7 
-- original counts are preserved in counts(dds)

R[write to console]: estimating dispersions

R[write to console]: fitting model and testing

 
 
 
 
  
 
 Converting df
 
 
 
 
  
 
 R[write to console]: estimating size factors

R[write to console]: estimating dispersions

R[write to console]: gene-wise dispersion estimates

R[write to console]: mean-dispersion relationship

R[write to console]: final dispersion estimates

R[write to console]: fitting model and testing

R[write to console]: -- replacing outliers and refitting for 2579 genes
-- DESeq argument 'minReplicatesForReplace' = 7 
-- original counts are preserved in counts(dds)

R[write to console]: estimating dispersions

R[write to console]: fitting model and testing

 
 
 
 
  
 
 Converting df
 
 
 
 
  
 
 R[write to console]: estimating size factors

R[write to console]: estimating dispersions

R[write to console]: gene-wise dispersion estimates

R[write to console]: mean-dispersion relationship

R[write to console]: final dispersion estimates

R[write to console]: fitting model and testing

R[write to console]: -- replacing outliers and refitting for 4019 genes
-- DESeq argument 'minReplicatesForReplace' = 7 
-- original counts are preserved in counts(dds)

R[write to console]: estimating dispersions

R[write to console]: fitting model and testing

 
 
 
 
  
 
 Converting df
 
 
 
 
  
 
 R[write to console]: estimating size factors

R[write to console]: estimating dispersions

R[write to console]: gene-wise dispersion estimates

R[write to console]: mean-dispersion relationship

R[write to console]: final dispersion estimates

R[write to console]: fitting model and testing

R[write to console]: -- replacing outliers and refitting for 2706 genes
-- DESeq argument 'minReplicatesForReplace' = 7 
-- original counts are preserved in counts(dds)

R[write to console]: estimating dispersions

R[write to console]: fitting model and testing

 
 
 
 
 
  
 
 
 
 
 In [31]: 
 
 
     def   ploting_log2fc_deseq2  (  dds_res  ,   genes  ,   title  ): 
     tmp   =   dds_res  .  loc  [  genes  ,[  "log2FoldChange"  ,   "lfcSE"  ,   "padj"  ]]  .  reset_index  () 
     tmp   =   tmp  .  rename  (  columns  =  {  'index'  :  'ERs'  }) 
     fig  ,   ax   =   plt  .  subplots  (  figsize  =  (  6  ,   4  )) 
     sns  .  barplot  (  data  =  tmp  ,   x  =  'ERs'  ,   y  =  'log2FoldChange'  ,   ax  =  ax  ) 
     x_coords   =   [  p  .  get_x  ()   +   0.5   *   p  .  get_width  ()   for   p   in   ax  .  patches  ] 
     y_coords   =   [  p  .  get_height  ()   for   p   in   ax  .  patches  ] 
     ax  .  set_ylim  ((  -  5  ,   6.5  )) 
     ax  .  set_title  (  title  ,   fontdict  =  {  'fontweight'   :   'bold'  }) 
     ax  .  bar_label  (  ax  .  containers  [  -  1  ],   labels  =  [  f  "padj:  {  p  :  .2e  }  "   for   p   in   tmp  [  'padj'  ]],   label_type  =  'edge'  ,   fontsize  =  7  ,   padding  =  16  ) 
     ax  .  errorbar  (  x  =  x_coords  ,   y  =  y_coords  ,   yerr  =  tmp  [  "lfcSE"  ],   fmt  =  "none"  ,   c  =  "k"  ) 

 ERs   =   [  "ESR1"  ,   "ESR2"  ,   "ESRRA"  ,   "ESRRB"  ,   "ESRRG"  ,   "GPER1"  ] 
 ploting_log2fc_deseq2  (  brca_deseq2_res  ,   ERs  ,   "BRCA"  ) 
 ploting_log2fc_deseq2  (  ov_deseq2_res  ,   ERs  ,   "OV"  ) 
 ploting_log2fc_deseq2  (  ucec_deseq2_res  ,   ERs  ,   "UCEC"  ) 
 ploting_log2fc_deseq2  (  cesc_deseq2_res  ,   ERs  ,   "CESC"  ) 
  
 
 
 
 
 
 
 
 
 
  
 
 /home/oscar/.local/lib/python3.10/site-packages/seaborn/_oldcore.py:1498: FutureWarning: is_categorical_dtype is deprecated and will be removed in a future version. Use isinstance(dtype, CategoricalDtype) instead
  if pd.api.types.is_categorical_dtype(vector):
/home/oscar/.local/lib/python3.10/site-packages/seaborn/_oldcore.py:1498: FutureWarning: is_categorical_dtype is deprecated and will be removed in a future version. Use isinstance(dtype, CategoricalDtype) instead
  if pd.api.types.is_categorical_dtype(vector):
/home/oscar/.local/lib/python3.10/site-packages/seaborn/_oldcore.py:1498: FutureWarning: is_categorical_dtype is deprecated and will be removed in a future version. Use isinstance(dtype, CategoricalDtype) instead
  if pd.api.types.is_categorical_dtype(vector):
/home/oscar/.local/lib/python3.10/site-packages/seaborn/_oldcore.py:1498: FutureWarning: is_categorical_dtype is deprecated and will be removed in a future version. Use isinstance(dtype, CategoricalDtype) instead
  if pd.api.types.is_categorical_dtype(vector):
/home/oscar/.local/lib/python3.10/site-packages/seaborn/_oldcore.py:1498: FutureWarning: is_categorical_dtype is deprecated and will be removed in a future version. Use isinstance(dtype, CategoricalDtype) instead
  if pd.api.types.is_categorical_dtype(vector):
/home/oscar/.local/lib/python3.10/site-packages/seaborn/_oldcore.py:1498: FutureWarning: is_categorical_dtype is deprecated and will be removed in a future version. Use isinstance(dtype, CategoricalDtype) instead
  if pd.api.types.is_categorical_dtype(vector):
/home/oscar/.local/lib/python3.10/site-packages/seaborn/_oldcore.py:1498: FutureWarning: is_categorical_dtype is deprecated and will be removed in a future version. Use isinstance(dtype, CategoricalDtype) instead
  if pd.api.types.is_categorical_dtype(vector):
/home/oscar/.local/lib/python3.10/site-packages/seaborn/_oldcore.py:1498: FutureWarning: is_categorical_dtype is deprecated and will be removed in a future version. Use isinstance(dtype, CategoricalDtype) instead
  if pd.api.types.is_categorical_dtype(vector):
/home/oscar/.local/lib/python3.10/site-packages/seaborn/_oldcore.py:1498: FutureWarning: is_categorical_dtype is deprecated and will be removed in a future version. Use isinstance(dtype, CategoricalDtype) instead
  if pd.api.types.is_categorical_dtype(vector):
/home/oscar/.local/lib/python3.10/site-packages/seaborn/_oldcore.py:1498: FutureWarning: is_categorical_dtype is deprecated and will be removed in a future version. Use isinstance(dtype, CategoricalDtype) instead
  if pd.api.types.is_categorical_dtype(vector):
/home/oscar/.local/lib/python3.10/site-packages/seaborn/_oldcore.py:1498: FutureWarning: is_categorical_dtype is deprecated and will be removed in a future version. Use isinstance(dtype, CategoricalDtype) instead
  if pd.api.types.is_categorical_dtype(vector):
/home/oscar/.local/lib/python3.10/site-packages/seaborn/_oldcore.py:1498: FutureWarning: is_categorical_dtype is deprecated and will be removed in a future version. Use isinstance(dtype, CategoricalDtype) instead
  if pd.api.types.is_categorical_dtype(vector):
 
 
 
 
  
 
 
 
 
 
  
 
 
 
 
 
  
 
 
 
 
 
  
 
 
 
 
 
 
  
 
 
 
 
 In [114]: 
 
 
     brca_gtex_gdc_tmm 
  
 
 
 
 
 
 
 
 
 
 Out[114]: 
 
 
 
 
 
 
  
 NAME 
 GTEX-1117F-2826-SM-5GZXL 
 GTEX-111YS-1926-SM-5GICC 
 GTEX-1122O-1226-SM-5H113 
 GTEX-117XS-1926-SM-5GICO 
 GTEX-117YX-1426-SM-5H12H 
 GTEX-1192X-2326-SM-5987X 
 GTEX-11DXW-0626-SM-5N9ER 
 GTEX-11DXY-2326-SM-5GICW 
 GTEX-11DXZ-1926-SM-5GZZL 
 ... 
 TCGA-AC-A5EH 
 TCGA-A1-A0SD 
 TCGA-A2-A0SU 
 TCGA-E9-A1NI 
 TCGA-B6-A0RQ 
 TCGA-A2-A0CX 
 TCGA-A2-A25F 
 TCGA-AC-A23G 
 TCGA-E2-A15D 
 TCGA-OL-A5D7 
 
 
 
 
 5S_rRNA 
 None 
 0.003827 
 0.001981 
 0.002036 
 0.003792 
 0.000000 
 0.002901 
 0.003889 
 0.001373 
 0.005507 
 ... 
 0.000000 
 0.001755 
 0.007500 
 0.002686 
 0.000000 
 0.000000 
 0.000000 
 0.003857 
 0.000000 
 0.000000 
 
 
 5_8S_rRNA 
 None 
 0.000000 
 0.000000 
 0.000000 
 0.000000 
 0.000000 
 0.000000 
 0.000000 
 0.000000 
 0.000000 
 ... 
 0.000000 
 0.002633 
 0.000000 
 0.000000 
 0.000000 
 0.000000 
 0.000000 
 0.000000 
 0.000000 
 0.000000 
 
 
 7SK 
 None 
 0.000000 
 0.000000 
 0.000000 
 0.000000 
 0.000000 
 0.000000 
 0.002593 
 0.000000 
 0.000000 
 ... 
 0.000000 
 0.000000 
 0.000000 
 0.000000 
 0.000000 
 0.005977 
 0.001900 
 0.000000 
 0.000000 
 0.000000 
 
 
 A1BG 
 None 
 6.001498 
 5.181340 
 5.602321 
 3.640725 
 1.674599 
 7.363725 
 7.218174 
 3.646930 
 2.713682 
 ... 
 0.211380 
 0.347529 
 0.112498 
 0.024170 
 0.274054 
 0.167352 
 0.226107 
 0.485959 
 0.284629 
 0.071593 
 
 
 A1BG-AS1 
 None 
 1.775954 
 2.963030 
 1.661153 
 0.788824 
 0.699833 
 2.521612 
 2.209010 
 1.010595 
 0.881066 
 ... 
 1.648760 
 1.563882 
 0.922483 
 1.111843 
 2.062616 
 1.004115 
 0.784724 
 2.950462 
 1.632871 
 1.121616 
 
 
 ... 
 ... 
 ... 
 ... 
 ... 
 ... 
 ... 
 ... 
 ... 
 ... 
 ... 
 ... 
 ... 
 ... 
 ... 
 ... 
 ... 
 ... 
 ... 
 ... 
 ... 
 ... 
 
 
 ZYX 
 None 
 435.169854 
 202.309954 
 412.422032 
 286.858795 
 265.011602 
 276.031384 
 292.382703 
 309.329985 
 186.556815 
 ... 
 513.736837 
 154.366164 
 155.652193 
 101.830294 
 257.120246 
 198.605558 
 225.973774 
 351.139743 
 194.821014 
 203.418199 
 
 
 ZYXP1 
 None 
 0.000000 
 0.000000 
 0.000000 
 0.000000 
 0.000000 
 0.000000 
 0.000000 
 0.000000 
 0.000000 
 ... 
 0.000000 
 0.000000 
 0.000000 
 0.000000 
 0.000000 
 0.000000 
 0.000000 
 0.000000 
 0.000000 
 0.000000 
 
 
 ZZEF1 
 None 
 103.903488 
 140.688446 
 131.214828 
 158.963158 
 99.351232 
 101.514204 
 92.389512 
 99.214077 
 124.001162 
 ... 
 58.002546 
 65.319698 
 32.151920 
 55.229580 
 76.605275 
 43.365212 
 115.952876 
 71.192923 
 76.490280 
 56.629679 
 
 
 ZZZ3 
 None 
 48.828515 
 60.591578 
 44.443995 
 42.839198 
 27.243483 
 55.104175 
 44.180201 
 46.948734 
 37.039995 
 ... 
 41.324700 
 67.246906 
 73.168680 
 37.560949 
 65.830630 
 39.495186 
 74.269426 
 41.896567 
 52.132036 
 20.905014 
 
 
 snoZ196 
 None 
 0.000000 
 0.000000 
 0.000000 
 0.000000 
 0.000000 
 0.000000 
 0.000000 
 0.000000 
 0.000000 
 ... 
 0.042276 
 0.000000 
 0.000000 
 0.000000 
 0.000000 
 0.000000 
 0.026601 
 0.000000 
 0.014980 
 0.000000 
 
 
 
 36004 rows × 1555 columns 
 
 
 
 
 
  
 
 
 
 
 In [32]: 
 
 
     brca_gtex_gdc_pheno 
  
 
 
 
 
 
 
 
 
 
 Out[32]: 
 
 array(['Normal', 'Normal', 'Normal', ..., 'Cancer', 'Cancer', 'Cancer'],
      dtype='<U6') 
 
 
 
 
  
 
 
 
 
 In [33]: 
 
 
     brca_gsea_hallmark   =   gsea  (  brca_gtex_gdc_tmm  ,   brca_gtex_gdc_pheno  ,   hallmark_gs  ) 
 ov_gsea_hallmark   =   gsea  (  ov_gtex_gdc_tmm  ,   ov_gtex_gdc_pheno  ,   hallmark_gs  ) 
 ucec_gsea_hallmark   =   gsea  (  ucec_gtex_gdc_tmm  ,   ucec_gtex_gdc_pheno  ,   hallmark_gs  ) 
 cesc_gsea_hallmark   =   gsea  (  cesc_gtex_gdc_tmm  ,   cesc_gtex_gdc_pheno  ,   hallmark_gs  ) 
  
 
 
 
 
 
 
 
 
 
  
 
 2023-12-18 11:47:22,713 [WARNING] Input data contains NA, filled NA with 0
/home/oscar/.local/lib/python3.10/site-packages/gseapy/gsea.py:116: FutureWarning: DataFrame.groupby with axis=1 is deprecated. Do `frame.T.groupby(...)` without axis instead.
  df_std = df.groupby(by=cls_dict, axis=1).std(numeric_only=True)
2023-12-18 11:48:00,435 [WARNING] Input data contains NA, filled NA with 0
/home/oscar/.local/lib/python3.10/site-packages/gseapy/gsea.py:116: FutureWarning: DataFrame.groupby with axis=1 is deprecated. Do `frame.T.groupby(...)` without axis instead.
  df_std = df.groupby(by=cls_dict, axis=1).std(numeric_only=True)
2023-12-18 11:48:18,561 [WARNING] Input data contains NA, filled NA with 0
/home/oscar/.local/lib/python3.10/site-packages/gseapy/gsea.py:116: FutureWarning: DataFrame.groupby with axis=1 is deprecated. Do `frame.T.groupby(...)` without axis instead.
  df_std = df.groupby(by=cls_dict, axis=1).std(numeric_only=True)
2023-12-18 11:48:40,330 [WARNING] Input data contains NA, filled NA with 0
/home/oscar/.local/lib/python3.10/site-packages/gseapy/gsea.py:116: FutureWarning: DataFrame.groupby with axis=1 is deprecated. Do `frame.T.groupby(...)` without axis instead.
  df_std = df.groupby(by=cls_dict, axis=1).std(numeric_only=True)
 
 
 
 
 
  
 
 
 
 
 In [34]: 
 
 
     brca_gsea_c6   =   gsea  (  brca_gtex_gdc_tmm  ,   brca_gtex_gdc_pheno  ,   c6_gs  ) 
 ov_gsea_c6   =   gsea  (  ov_gtex_gdc_tmm  ,   ov_gtex_gdc_pheno  ,   c6_gs  ) 
 ucec_gsea_c6   =   gsea  (  ucec_gtex_gdc_tmm  ,   ucec_gtex_gdc_pheno  ,   c6_gs  ) 
 cesc_gsea_c6   =   gsea  (  cesc_gtex_gdc_tmm  ,   cesc_gtex_gdc_pheno  ,   c6_gs  ) 
  
 
 
 
 
 
 
 
 
 
  
 
 2023-12-18 11:48:53,858 [WARNING] Input data contains NA, filled NA with 0
/home/oscar/.local/lib/python3.10/site-packages/gseapy/gsea.py:116: FutureWarning: DataFrame.groupby with axis=1 is deprecated. Do `frame.T.groupby(...)` without axis instead.
  df_std = df.groupby(by=cls_dict, axis=1).std(numeric_only=True)
2023-12-18 11:49:44,843 [WARNING] Input data contains NA, filled NA with 0
/home/oscar/.local/lib/python3.10/site-packages/gseapy/gsea.py:116: FutureWarning: DataFrame.groupby with axis=1 is deprecated. Do `frame.T.groupby(...)` without axis instead.
  df_std = df.groupby(by=cls_dict, axis=1).std(numeric_only=True)
2023-12-18 11:50:15,921 [WARNING] Input data contains NA, filled NA with 0
/home/oscar/.local/lib/python3.10/site-packages/gseapy/gsea.py:116: FutureWarning: DataFrame.groupby with axis=1 is deprecated. Do `frame.T.groupby(...)` without axis instead.
  df_std = df.groupby(by=cls_dict, axis=1).std(numeric_only=True)
2023-12-18 11:50:50,355 [WARNING] Input data contains NA, filled NA with 0
/home/oscar/.local/lib/python3.10/site-packages/gseapy/gsea.py:116: FutureWarning: DataFrame.groupby with axis=1 is deprecated. Do `frame.T.groupby(...)` without axis instead.
  df_std = df.groupby(by=cls_dict, axis=1).std(numeric_only=True)
 
 
 
 
 
  
 
 
 
 
 In [30]: 
 
 
     brca_c6_es   =   gsva_py2r  (  np  .  log2  (  brca_gdc_tpm  +  1  ),  c6_gs  ) 
 ov_c6_es   =   gsva_py2r  (  np  .  log2  (  ov_gdc_tpm  +  1  ),  c6_gs  ) 
 ucec_c6_es   =   gsva_py2r  (  np  .  log2  (  ucec_gdc_tpm  +  1  ),  c6_gs  ) 
 cesc_c6_es   =   gsva_py2r  (  np  .  log2  (  cesc_gdc_tpm  +  1  ),  c6_gs  ) 
  
 
 
 
 
 
 
 
 
 
  
 
 Converting df
Estimating GSVA scores for 189 gene sets.
Estimating ECDFs with Gaussian kernels
  |======================================================================| 100%

Converting df
Estimating GSVA scores for 189 gene sets.
Estimating ECDFs with Gaussian kernels
  |======================================================================| 100%

Converting df
Estimating GSVA scores for 189 gene sets.
Estimating ECDFs with Gaussian kernels
  |======================================================================| 100%

Converting df
Estimating GSVA scores for 189 gene sets.
Estimating ECDFs with Gaussian kernels
  |======================================================================| 100%

 
 
 
 
 
  
 
 
 
 
 In [35]: 
 
 
     plotting_gsea  (  brca_gsea_hallmark  ,   "HALLMARK_ESTROGEN_RESPONSE_EARLY"  ) 
 plotting_gsea  (  ov_gsea_hallmark  ,   "HALLMARK_ESTROGEN_RESPONSE_EARLY"  ) 
 plotting_gsea  (  ucec_gsea_hallmark  ,   "HALLMARK_ESTROGEN_RESPONSE_EARLY"  ) 
 plotting_gsea  (  cesc_gsea_hallmark  ,   "HALLMARK_ESTROGEN_RESPONSE_EARLY"  ) 
  
 
 
 
 
 
 
 
 
 
  
 
 /home/oscar/.local/lib/python3.10/site-packages/gseapy/plot.py:602: FutureWarning: The 'method' keyword in Series.replace is deprecated and will be removed in a future version.
  df[self.colname].replace(
/home/oscar/.local/lib/python3.10/site-packages/gseapy/plot.py:602: FutureWarning: The 'method' keyword in Series.replace is deprecated and will be removed in a future version.
  df[self.colname].replace(
/home/oscar/.local/lib/python3.10/site-packages/gseapy/plot.py:602: FutureWarning: The 'method' keyword in Series.replace is deprecated and will be removed in a future version.
  df[self.colname].replace(
/home/oscar/.local/lib/python3.10/site-packages/gseapy/plot.py:602: FutureWarning: The 'method' keyword in Series.replace is deprecated and will be removed in a future version.
  df[self.colname].replace(
 
 
 
 
  
 
 
 
 
 
  
 
 
 
 
 
  
 
 
 
 
 
  
 
 
 
 
 
  
 
 
 
 
 
  
 
 
 
 
 
  
 
 
 
 
 
  
 
 
 
 
 
  
 
 
 
 
 
  
 
 
 
 
 
  
 
 
 
 
 
  
 
 
 
 
 
 
  
 
 
 
 
 In [36]: 
 
 
     plotting_gsea  (  brca_gsea_hallmark  ,   "HALLMARK_ESTROGEN_RESPONSE_LATE"  ) 
 plotting_gsea  (  ov_gsea_hallmark  ,   "HALLMARK_ESTROGEN_RESPONSE_LATE"  ) 
 plotting_gsea  (  ucec_gsea_hallmark  ,   "HALLMARK_ESTROGEN_RESPONSE_LATE"  ) 
 plotting_gsea  (  cesc_gsea_hallmark  ,   "HALLMARK_ESTROGEN_RESPONSE_LATE"  ) 
  
 
 
 
 
 
 
 
 
 
  
 
 /home/oscar/.local/lib/python3.10/site-packages/gseapy/plot.py:602: FutureWarning: The 'method' keyword in Series.replace is deprecated and will be removed in a future version.
  df[self.colname].replace(
/home/oscar/.local/lib/python3.10/site-packages/gseapy/plot.py:602: FutureWarning: The 'method' keyword in Series.replace is deprecated and will be removed in a future version.
  df[self.colname].replace(
/home/oscar/.local/lib/python3.10/site-packages/gseapy/plot.py:602: FutureWarning: The 'method' keyword in Series.replace is deprecated and will be removed in a future version.
  df[self.colname].replace(
/home/oscar/.local/lib/python3.10/site-packages/gseapy/plot.py:602: FutureWarning: The 'method' keyword in Series.replace is deprecated and will be removed in a future version.
  df[self.colname].replace(
 
 
 
 
  
 
 
 
 
 
  
 
 
 
 
 
  
 
 
 
 
 
  
 
 
 
 
 
  
 
 
 
 
 
  
 
 
 
 
 
  
 
 
 
 
 
  
 
 
 
 
 
  
 
 
 
 
 
  
 
 
 
 
 
  
 
 
 
 
 
  
 
 
 
 
 
 
 
 
 
 
 
  
  
 Figure 5 ¶  
 
 
 
  
 
 
 
 
 In [33]: 
 
 
     brca_pheno_bestes   =   np  .  full  (  len  (  brca_es  ),   "High EERES"  ) 
 brca_pheno_bestes  [  brca_es  [  "EARLY"  ]  <=  brca_bestes  ]   =   "Low EERES" 
 brca_sortq   =   np  .  argsort  (  brca_pheno_bestes  )[::  -  1  ] 
 brca_pheno_bestes   =   brca_pheno_bestes  [  brca_sortq  ] 
 brca_gdc_tmm   =   genecount_to_tmm_for_gsea  (  brca_gdc_genecount  .  iloc  [:,  brca_sortq  ]) 
 brca_gsea_bestes_c6   =   gsea  (  brca_gdc_tmm  ,   brca_pheno_bestes  ,   c6_gs  ) 
 brca_gsea_bestes_c3   =   gsea  (  brca_gdc_tmm  ,   brca_pheno_bestes  ,   c3_gs  ) 
 brca_gsea_bestes_hm   =   gsea  (  brca_gdc_tmm  ,   brca_pheno_bestes  ,   hallmark_gs  ) 
  
 
 
 
 
 
 
 
 
 
  
 
 2023-05-30 21:44:49,557 [WARNING] Input data contains NA, filled NA with 0
2023-05-30 21:45:54,428 [WARNING] Input data contains NA, filled NA with 0
2023-05-30 21:47:09,522 [WARNING] Input data contains NA, filled NA with 0
 
 
 
 
 
  
 
 
 
 
 In [34]: 
 
 
     brca_pheno_bestes_df   =   pd  .  DataFrame  (  brca_pheno_bestes  ,   index  =  brca_es  .  iloc  [  brca_sortq  ]  .  index  ,   columns  =  [  "EERES"  ]) 
 brca_eeres_deseq2_res   =   deseq2_py2r  (  brca_gdc_genecount  .  iloc  [:,  brca_sortq  ],   brca_pheno_bestes_df  ,   "~EERES"  ,   [  "EERES"  ,   "High EERES"  ,   "Low EERES"  ]) 
  
 
 
 
 
 
 
 
 
 
  
 
 Converting df
 
 
 
 
  
 
 R[write to console]:   Note: levels of factors in the design contain characters other than
  letters, numbers, '_' and '.'. It is recommended (but not required) to use
  only letters, numbers, and delimiters '_' or '.', as these are safe characters
  for column names in R. [This is a message, not a warning or an error]

R[write to console]: estimating size factors

R[write to console]:   Note: levels of factors in the design contain characters other than
  letters, numbers, '_' and '.'. It is recommended (but not required) to use
  only letters, numbers, and delimiters '_' or '.', as these are safe characters
  for column names in R. [This is a message, not a warning or an error]

R[write to console]: estimating dispersions

R[write to console]: gene-wise dispersion estimates

R[write to console]: mean-dispersion relationship

R[write to console]:   Note: levels of factors in the design contain characters other than
  letters, numbers, '_' and '.'. It is recommended (but not required) to use
  only letters, numbers, and delimiters '_' or '.', as these are safe characters
  for column names in R. [This is a message, not a warning or an error]

R[write to console]: final dispersion estimates

R[write to console]:   Note: levels of factors in the design contain characters other than
  letters, numbers, '_' and '.'. It is recommended (but not required) to use
  only letters, numbers, and delimiters '_' or '.', as these are safe characters
  for column names in R. [This is a message, not a warning or an error]

R[write to console]: fitting model and testing

R[write to console]:   Note: levels of factors in the design contain characters other than
  letters, numbers, '_' and '.'. It is recommended (but not required) to use
  only letters, numbers, and delimiters '_' or '.', as these are safe characters
  for column names in R. [This is a message, not a warning or an error]

R[write to console]: -- replacing outliers and refitting for 11378 genes
-- DESeq argument 'minReplicatesForReplace' = 7 
-- original counts are preserved in counts(dds)

R[write to console]: estimating dispersions

R[write to console]:   Note: levels of factors in the design contain characters other than
  letters, numbers, '_' and '.'. It is recommended (but not required) to use
  only letters, numbers, and delimiters '_' or '.', as these are safe characters
  for column names in R. [This is a message, not a warning or an error]

R[write to console]: fitting model and testing

R[write to console]:   Note: levels of factors in the design contain characters other than
  letters, numbers, '_' and '.'. It is recommended (but not required) to use
  only letters, numbers, and delimiters '_' or '.', as these are safe characters
  for column names in R. [This is a message, not a warning or an error]

 
 
 
 
 
  
 
 
 
 
 In [35]: 
 
 
     ov_pheno_bestes   =   np  .  full  (  len  (  ov_es  ),   "High EERES"  ) 
 ov_pheno_bestes  [  ov_es  [  "EARLY"  ]  <=  ov_bestes  ]   =   "Low EERES" 
 ov_sortq   =   np  .  argsort  (  ov_pheno_bestes  )[::  -  1  ] 
 ov_pheno_bestes   =   ov_pheno_bestes  [  ov_sortq  ] 
 ov_gdc_tmm   =   genecount_to_tmm_for_gsea  (  ov_gdc_genecount  .  iloc  [:,  ov_sortq  ]) 
 ov_gsea_bestes_c6   =   gsea  (  ov_gdc_tmm  ,   ov_pheno_bestes  ,   c6_gs  ) 
 ov_gsea_bestes_c3   =   gsea  (  ov_gdc_tmm  ,   ov_pheno_bestes  ,   c3_gs  ) 
 ov_gsea_bestes_hm   =   gsea  (  ov_gdc_tmm  ,   ov_pheno_bestes  ,   hallmark_gs  ) 
  
 
 
 
 
 
 
 
 
 
  
 
 2023-05-30 21:54:16,545 [WARNING] Input data contains NA, filled NA with 0
2023-05-30 21:54:59,956 [WARNING] Input data contains NA, filled NA with 0
2023-05-30 21:55:53,786 [WARNING] Input data contains NA, filled NA with 0
 
 
 
 
 
  
 
 
 
 
 In [36]: 
 
 
     ov_pheno_bestes_df   =   pd  .  DataFrame  (  ov_pheno_bestes  ,   index  =  ov_es  .  iloc  [  ov_sortq  ]  .  index  ,   columns  =  [  "EERES"  ]) 
 ov_pheno_bestes_df 
 ov_eeres_deseq2_res   =   deseq2_py2r  (  ov_gdc_genecount  .  iloc  [:,  ov_sortq  ],   ov_pheno_bestes_df  ,   "~EERES"  ,   [  "EERES"  ,   "High EERES"  ,   "Low EERES"  ]) 
  
 
 
 
 
 
 
 
 
 
  
 
 Converting df
 
 
 
 
  
 
 R[write to console]:   Note: levels of factors in the design contain characters other than
  letters, numbers, '_' and '.'. It is recommended (but not required) to use
  only letters, numbers, and delimiters '_' or '.', as these are safe characters
  for column names in R. [This is a message, not a warning or an error]

R[write to console]: estimating size factors

R[write to console]:   Note: levels of factors in the design contain characters other than
  letters, numbers, '_' and '.'. It is recommended (but not required) to use
  only letters, numbers, and delimiters '_' or '.', as these are safe characters
  for column names in R. [This is a message, not a warning or an error]

R[write to console]: estimating dispersions

R[write to console]: gene-wise dispersion estimates

R[write to console]: mean-dispersion relationship

R[write to console]:   Note: levels of factors in the design contain characters other than
  letters, numbers, '_' and '.'. It is recommended (but not required) to use
  only letters, numbers, and delimiters '_' or '.', as these are safe characters
  for column names in R. [This is a message, not a warning or an error]

R[write to console]: final dispersion estimates

R[write to console]:   Note: levels of factors in the design contain characters other than
  letters, numbers, '_' and '.'. It is recommended (but not required) to use
  only letters, numbers, and delimiters '_' or '.', as these are safe characters
  for column names in R. [This is a message, not a warning or an error]

R[write to console]: fitting model and testing

R[write to console]:   Note: levels of factors in the design contain characters other than
  letters, numbers, '_' and '.'. It is recommended (but not required) to use
  only letters, numbers, and delimiters '_' or '.', as these are safe characters
  for column names in R. [This is a message, not a warning or an error]

R[write to console]: -- replacing outliers and refitting for 5278 genes
-- DESeq argument 'minReplicatesForReplace' = 7 
-- original counts are preserved in counts(dds)

R[write to console]: estimating dispersions

R[write to console]:   Note: levels of factors in the design contain characters other than
  letters, numbers, '_' and '.'. It is recommended (but not required) to use
  only letters, numbers, and delimiters '_' or '.', as these are safe characters
  for column names in R. [This is a message, not a warning or an error]

R[write to console]: fitting model and testing

R[write to console]:   Note: levels of factors in the design contain characters other than
  letters, numbers, '_' and '.'. It is recommended (but not required) to use
  only letters, numbers, and delimiters '_' or '.', as these are safe characters
  for column names in R. [This is a message, not a warning or an error]

 
 
 
 
 
  
 
 
 
 
 In [37]: 
 
 
     ucec_pheno_bestes   =   np  .  full  (  len  (  ucec_es  ),   "High EERES"  ) 
 ucec_pheno_bestes  [  ucec_es  [  "EARLY"  ]  <=  ucec_bestes  ]   =   "Low EERES" 
 ucec_sortq   =   np  .  argsort  (  ucec_pheno_bestes  )[::  -  1  ] 
 ucec_pheno_bestes   =   ucec_pheno_bestes  [  ucec_sortq  ] 
 ucec_gdc_tmm   =   genecount_to_tmm_for_gsea  (  ucec_gdc_genecount  .  iloc  [:,  ucec_sortq  ]) 
 ucec_gsea_bestes_c6   =   gsea  (  ucec_gdc_tmm  ,   ucec_pheno_bestes  ,   c6_gs  ) 
 ucec_gsea_bestes_c3   =   gsea  (  ucec_gdc_tmm  ,   ucec_pheno_bestes  ,   c3_gs  ) 
 ucec_gsea_bestes_hm   =   gsea  (  ucec_gdc_tmm  ,   ucec_pheno_bestes  ,   hallmark_gs  ) 
  
 
 
 
 
 
 
 
 
 
  
 
 2023-05-30 21:58:12,638 [WARNING] Input data contains NA, filled NA with 0
2023-05-30 21:59:02,953 [WARNING] Input data contains NA, filled NA with 0
2023-05-30 22:00:01,337 [WARNING] Input data contains NA, filled NA with 0
 
 
 
 
 
  
 
 
 
 
 In [38]: 
 
 
     ucec_pheno_bestes_df   =   pd  .  DataFrame  (  ucec_pheno_bestes  ,   index  =  ucec_es  .  iloc  [  ucec_sortq  ]  .  index  ,   columns  =  [  "EERES"  ]) 
 ucec_pheno_bestes_df 
 ucec_eeres_deseq2_res   =   deseq2_py2r  (  ucec_gdc_genecount  .  iloc  [:,  ucec_sortq  ],   ucec_pheno_bestes_df  ,   "~EERES"  ,   [  "EERES"  ,   "High EERES"  ,   "Low EERES"  ]) 
  
 
 
 
 
 
 
 
 
 
  
 
 Converting df
 
 
 
 
  
 
 R[write to console]:   Note: levels of factors in the design contain characters other than
  letters, numbers, '_' and '.'. It is recommended (but not required) to use
  only letters, numbers, and delimiters '_' or '.', as these are safe characters
  for column names in R. [This is a message, not a warning or an error]

R[write to console]: estimating size factors

R[write to console]:   Note: levels of factors in the design contain characters other than
  letters, numbers, '_' and '.'. It is recommended (but not required) to use
  only letters, numbers, and delimiters '_' or '.', as these are safe characters
  for column names in R. [This is a message, not a warning or an error]

R[write to console]: estimating dispersions

R[write to console]: gene-wise dispersion estimates

R[write to console]: mean-dispersion relationship

R[write to console]:   Note: levels of factors in the design contain characters other than
  letters, numbers, '_' and '.'. It is recommended (but not required) to use
  only letters, numbers, and delimiters '_' or '.', as these are safe characters
  for column names in R. [This is a message, not a warning or an error]

R[write to console]: final dispersion estimates

R[write to console]:   Note: levels of factors in the design contain characters other than
  letters, numbers, '_' and '.'. It is recommended (but not required) to use
  only letters, numbers, and delimiters '_' or '.', as these are safe characters
  for column names in R. [This is a message, not a warning or an error]

R[write to console]: fitting model and testing

R[write to console]:   Note: levels of factors in the design contain characters other than
  letters, numbers, '_' and '.'. It is recommended (but not required) to use
  only letters, numbers, and delimiters '_' or '.', as these are safe characters
  for column names in R. [This is a message, not a warning or an error]

R[write to console]: -- replacing outliers and refitting for 8291 genes
-- DESeq argument 'minReplicatesForReplace' = 7 
-- original counts are preserved in counts(dds)

R[write to console]: estimating dispersions

R[write to console]:   Note: levels of factors in the design contain characters other than
  letters, numbers, '_' and '.'. It is recommended (but not required) to use
  only letters, numbers, and delimiters '_' or '.', as these are safe characters
  for column names in R. [This is a message, not a warning or an error]

R[write to console]: fitting model and testing

R[write to console]:   Note: levels of factors in the design contain characters other than
  letters, numbers, '_' and '.'. It is recommended (but not required) to use
  only letters, numbers, and delimiters '_' or '.', as these are safe characters
  for column names in R. [This is a message, not a warning or an error]

 
 
 
 
 
  
 
 
 
 
 In [39]: 
 
 
     cesc_pheno_bestes   =   np  .  full  (  len  (  cesc_es  ),   "High EERES"  ) 
 cesc_pheno_bestes  [  cesc_es  [  "EARLY"  ]  <=  cesc_bestes  ]   =   "Low EERES" 
 cesc_sortq   =   np  .  argsort  (  cesc_pheno_bestes  )[::  -  1  ] 
 cesc_pheno_bestes   =   cesc_pheno_bestes  [  cesc_sortq  ] 
 cesc_gdc_tmm   =   genecount_to_tmm_for_gsea  (  cesc_gdc_genecount  .  iloc  [:,  cesc_sortq  ]) 
 cesc_gsea_bestes_c6   =   gsea  (  cesc_gdc_tmm  ,   cesc_pheno_bestes  ,   c6_gs  ) 
 cesc_gsea_bestes_c3   =   gsea  (  cesc_gdc_tmm  ,   cesc_pheno_bestes  ,   c3_gs  ) 
 cesc_gsea_bestes_hm   =   gsea  (  cesc_gdc_tmm  ,   cesc_pheno_bestes  ,   hallmark_gs  ) 
  
 
 
 
 
 
 
 
 
 
  
 
 2023-05-30 22:03:33,299 [WARNING] Input data contains NA, filled NA with 0
2023-05-30 22:04:13,490 [WARNING] Input data contains NA, filled NA with 0
2023-05-30 22:05:03,477 [WARNING] Input data contains NA, filled NA with 0
 
 
 
 
 
  
 
 
 
 
 In [40]: 
 
 
     cesc_pheno_bestes_df   =   pd  .  DataFrame  (  cesc_pheno_bestes  ,   index  =  cesc_es  .  iloc  [  cesc_sortq  ]  .  index  ,   columns  =  [  "EERES"  ]) 
 cesc_pheno_bestes_df 
 cesc_eeres_deseq2_res   =   deseq2_py2r  (  cesc_gdc_genecount  .  iloc  [:,  cesc_sortq  ],   cesc_pheno_bestes_df  ,   "~EERES"  ,   [  "EERES"  ,   "High EERES"  ,   "Low EERES"  ]) 
  
 
 
 
 
 
 
 
 
 
  
 
 Converting df
 
 
 
 
  
 
 R[write to console]:   Note: levels of factors in the design contain characters other than
  letters, numbers, '_' and '.'. It is recommended (but not required) to use
  only letters, numbers, and delimiters '_' or '.', as these are safe characters
  for column names in R. [This is a message, not a warning or an error]

R[write to console]: estimating size factors

R[write to console]:   Note: levels of factors in the design contain characters other than
  letters, numbers, '_' and '.'. It is recommended (but not required) to use
  only letters, numbers, and delimiters '_' or '.', as these are safe characters
  for column names in R. [This is a message, not a warning or an error]

R[write to console]: estimating dispersions

R[write to console]: gene-wise dispersion estimates

R[write to console]: mean-dispersion relationship

R[write to console]:   Note: levels of factors in the design contain characters other than
  letters, numbers, '_' and '.'. It is recommended (but not required) to use
  only letters, numbers, and delimiters '_' or '.', as these are safe characters
  for column names in R. [This is a message, not a warning or an error]

R[write to console]: final dispersion estimates

R[write to console]:   Note: levels of factors in the design contain characters other than
  letters, numbers, '_' and '.'. It is recommended (but not required) to use
  only letters, numbers, and delimiters '_' or '.', as these are safe characters
  for column names in R. [This is a message, not a warning or an error]

R[write to console]: fitting model and testing

R[write to console]:   Note: levels of factors in the design contain characters other than
  letters, numbers, '_' and '.'. It is recommended (but not required) to use
  only letters, numbers, and delimiters '_' or '.', as these are safe characters
  for column names in R. [This is a message, not a warning or an error]

R[write to console]: -- replacing outliers and refitting for 6626 genes
-- DESeq argument 'minReplicatesForReplace' = 7 
-- original counts are preserved in counts(dds)

R[write to console]: estimating dispersions

R[write to console]:   Note: levels of factors in the design contain characters other than
  letters, numbers, '_' and '.'. It is recommended (but not required) to use
  only letters, numbers, and delimiters '_' or '.', as these are safe characters
  for column names in R. [This is a message, not a warning or an error]

R[write to console]: fitting model and testing

R[write to console]:   Note: levels of factors in the design contain characters other than
  letters, numbers, '_' and '.'. It is recommended (but not required) to use
  only letters, numbers, and delimiters '_' or '.', as these are safe characters
  for column names in R. [This is a message, not a warning or an error]

 
 
 
 
 
  
 
 
 
 
 In [41]: 
 
 
     plotting_gsea  (  brca_gsea_bestes_c6  ,   "MEK_UP.V1_DN"  ) 
 plotting_gsea  (  ov_gsea_bestes_c6  ,   "MEK_UP.V1_UP"  ) 
 plotting_gsea  (  ucec_gsea_bestes_c6  ,   "MEK_UP.V1_UP"  ) 
 plotting_gsea  (  cesc_gsea_bestes_c6  ,   "MEK_UP.V1_UP"  ) 
  
 
 
 
 
 
 
 
 
 
  
 
 
 
 
 
  
 
 
 
 
 
  
 
 
 
 
 
  
 
 
 
 
 
  
 
 
 
 
 
  
 
 
 
 
 
  
 
 
 
 
 
  
 
 
 
 
 
  
 
 
 
 
 
  
 
 
 
 
 
  
 
 
 
 
 
  
 
 
 
 
 
 
  
 
 
 
 
 In [42]: 
 
 
     plotting_gsea  (  brca_gsea_bestes_c6  ,   "MEK_UP.V1_UP"  ) 
 plotting_gsea  (  ov_gsea_bestes_c6  ,   "MEK_UP.V1_DN"  ) 
 plotting_gsea  (  ucec_gsea_bestes_c6  ,   "MEK_UP.V1_DN"  ) 
 plotting_gsea  (  cesc_gsea_bestes_c6  ,   "MEK_UP.V1_DN"  ) 
  
 
 
 
 
 
 
 
 
 
  
 
 
 
 
 
  
 
 
 
 
 
  
 
 
 
 
 
  
 
 
 
 
 
  
 
 
 
 
 
  
 
 
 
 
 
  
 
 
 
 
 
  
 
 
 
 
 
  
 
 
 
 
 
  
 
 
 
 
 
  
 
 
 
 
 
  
 
 
 
 
 
 
  
 
 
 
 
 In [43]: 
 
 
     # CCLE 

 ccle_log2tpm   =   pd  .  read_csv  (  "Data/CCLE/OmicsExpressionProteinCodingGenesTPMLogp1.csv"  ,   index_col  =  0  ) 
 ccle_log2tpm  .  columns   =   [  c  .  split  (  "("  )[  0  ][:  -  1  ]   for   c   in   ccle_log2tpm  .  columns  ] 
  
 
 
 
 
  
 
 
 
 
 In [44]: 
 
 
     ccle_model   =   pd  .  read_csv  (  "Data/CCLE/Model.csv"  ,   index_col  =  0  )[[  "COSMICID"  ]] 
 ccle_model   =   ccle_model  .  reset_index  ()  .  set_index  (  "COSMICID"  ) 
  
 
 
 
 
  
 
 
 
 
 In [90]: 
 
 
     ccle_selected_genes   =   ccle_log2tpm  [[  "MALL"  ,   "NT5C2"  ,   "CDC42BPB"  ,   "MYO5A"  ,   "TNFRSF21"  ,   "PRKCH"  ,   "PCDH1"  ,   "SCAMP4"  ,   "MPZL2"  ,   "TTC9"  ,   "INAVA"  ,   "EPHA2"  ,   "EGFR"  ,   "MAPK1"  ,   "RAF1"  ,   "MAP2K1"  ]] 
  
 
 
 
 
  
 
 
 
 
 In [46]: 
 
 
     ccle_ic50   =   pd  .  read_excel  (  "Data/CCLE/GDSC2_fitted_dose_response_24Jul22.xlsx"  ,   index_col  =  3  )[[  "DRUG_NAME"  ,   "DRUG_ID"  ,   "LN_IC50"  ,   "AUC"  ]] 
  
 
 
 
 
  
 
 
 
 
 In [47]: 
 
 
     def   plotting_scatter_spearman  (  x  ,   y  ,   xlabel  ,   ylabel  ):   
     from   scipy.stats   import   spearmanr 
     plt  .  scatter  (  x  ,   y  ) 
     plt  .  xlabel  (  xlabel  ,   weight  =  "bold"  ,   fontsize  =  12  ,   labelpad  =  4  ) 
     plt  .  ylabel  (  ylabel  ,   weight  =  "bold"  ,   fontsize  =  12  ,   labelpad  =  4  ) 
     sr  ,   sp   =   spearmanr  (  x  ,   y  ) 
     plt  .  title  (  f  "Spearman R=  {  sr  :  .3f  }  , p=  {  sp  :  .3e  }  "  ,   weight  =  "bold"  ) 
     plt  .  show  () 
    
 ccle_model_ic50   =   ccle_model  .  join  (  ccle_ic50  ,   how  =  'inner'  )  .  reset_index  ()  .  set_index  (  "ModelID"  ) 
 ccle_model_ic50_esr1_eeres   =   ccle_model_ic50  .  join  (  ccle_selected_genes  ,   how  =  "inner"  ) 
 for   did   in   [  1199  ,  1200  ,  1816  ,  1372  ]: 
     ccle_model_ic50_esr1_eeres_tamoxifen   =   ccle_model_ic50_esr1_eeres  [  ccle_model_ic50_esr1_eeres  [  "DRUG_ID"  ]  ==  did  ] 
     for   g   in   [  "MALL"  ,   "NT5C2"  ,   "CDC42BPB"  ,   "MYO5A"  ,   "TNFRSF21"  ,   "PRKCH"  ,   "PCDH1"  ,   "SCAMP4"  ,   "MPZL2"  ,   "TTC9"  ,   "INAVA"  ,   "EPHA2"  ]: 
         print  (  g  ) 
         print  (  did  ,   ccle_model_ic50_esr1_eeres_tamoxifen  [  "DRUG_NAME"  ][  0  ]) 
         dn   =   ccle_model_ic50_esr1_eeres_tamoxifen  [  "DRUG_NAME"  ][  0  ] 
         s  ,   p   =   spearmanr  (  ccle_model_ic50_esr1_eeres_tamoxifen  [  g  ],   ccle_model_ic50_esr1_eeres_tamoxifen  [  "LN_IC50"  ]) 
         plotting_scatter_spearman  (  ccle_model_ic50_esr1_eeres_tamoxifen  [  g  ]  .  values  ,   ccle_model_ic50_esr1_eeres_tamoxifen  [  "LN_IC50"  ]  .  values  ,   f  "log2[  {  g  }  +1]"  ,   f  "  {  dn  }   LN_IC50"  ) 
         plt  .  show  () 
  
 
 
 
 
 
 
 
 
 
  
 
 MALL
1199 Tamoxifen
 
 
 
 
  
 
 
 
 
 
  
 
 NT5C2
1199 Tamoxifen
 
 
 
 
  
 
 
 
 
 
  
 
 CDC42BPB
1199 Tamoxifen
 
 
 
 
  
 
 
 
 
 
  
 
 MYO5A
1199 Tamoxifen
 
 
 
 
  
 
 
 
 
 
  
 
 TNFRSF21
1199 Tamoxifen
 
 
 
 
  
 
 
 
 
 
  
 
 PRKCH
1199 Tamoxifen
 
 
 
 
  
 
 
 
 
 
  
 
 PCDH1
1199 Tamoxifen
 
 
 
 
  
 
 
 
 
 
  
 
 SCAMP4
1199 Tamoxifen
 
 
 
 
  
 
 
 
 
 
  
 
 MPZL2
1199 Tamoxifen
 
 
 
 
  
 
 
 
 
 
  
 
 TTC9
1199 Tamoxifen
 
 
 
 
  
 
 
 
 
 
  
 
 INAVA
1199 Tamoxifen
 
 
 
 
  
 
 
 
 
 
  
 
 EPHA2
1199 Tamoxifen
 
 
 
 
  
 
 
 
 
 
  
 
 MALL
1200 Fulvestrant
 
 
 
 
  
 
 
 
 
 
  
 
 NT5C2
1200 Fulvestrant
 
 
 
 
  
 
 
 
 
 
  
 
 CDC42BPB
1200 Fulvestrant
 
 
 
 
  
 
 
 
 
 
  
 
 MYO5A
1200 Fulvestrant
 
 
 
 
  
 
 
 
 
 
  
 
 TNFRSF21
1200 Fulvestrant
 
 
 
 
  
 
 
 
 
 
  
 
 PRKCH
1200 Fulvestrant
 
 
 
 
  
 
 
 
 
 
  
 
 PCDH1
1200 Fulvestrant
 
 
 
 
  
 
 
 
 
 
  
 
 SCAMP4
1200 Fulvestrant
 
 
 
 
  
 
 
 
 
 
  
 
 MPZL2
1200 Fulvestrant
 
 
 
 
  
 
 
 
 
 
  
 
 TTC9
1200 Fulvestrant
 
 
 
 
  
 
 
 
 
 
  
 
 INAVA
1200 Fulvestrant
 
 
 
 
  
 
 
 
 
 
  
 
 EPHA2
1200 Fulvestrant
 
 
 
 
  
 
 
 
 
 
  
 
 MALL
1816 Fulvestrant
 
 
 
 
  
 
 
 
 
 
  
 
 NT5C2
1816 Fulvestrant
 
 
 
 
  
 
 
 
 
 
  
 
 CDC42BPB
1816 Fulvestrant
 
 
 
 
  
 
 
 
 
 
  
 
 MYO5A
1816 Fulvestrant
 
 
 
 
  
 
 
 
 
 
  
 
 TNFRSF21
1816 Fulvestrant
 
 
 
 
  
 
 
 
 
 
  
 
 PRKCH
1816 Fulvestrant
 
 
 
 
  
 
 
 
 
 
  
 
 PCDH1
1816 Fulvestrant
 
 
 
 
  
 
 
 
 
 
  
 
 SCAMP4
1816 Fulvestrant
 
 
 
 
  
 
 
 
 
 
  
 
 MPZL2
1816 Fulvestrant
 
 
 
 
  
 
 
 
 
 
  
 
 TTC9
1816 Fulvestrant
 
 
 
 
  
 
 
 
 
 
  
 
 INAVA
1816 Fulvestrant
 
 
 
 
  
 
 
 
 
 
  
 
 EPHA2
1816 Fulvestrant
 
 
 
 
  
 
 
 
 
 
  
 
 MALL
1372 Trametinib
 
 
 
 
  
 
 
 
 
 
  
 
 NT5C2
1372 Trametinib
 
 
 
 
  
 
 
 
 
 
  
 
 CDC42BPB
1372 Trametinib
 
 
 
 
  
 
 
 
 
 
  
 
 MYO5A
1372 Trametinib
 
 
 
 
  
 
 
 
 
 
  
 
 TNFRSF21
1372 Trametinib
 
 
 
 
  
 
 
 
 
 
  
 
 PRKCH
1372 Trametinib
 
 
 
 
  
 
 
 
 
 
  
 
 PCDH1
1372 Trametinib
 
 
 
 
  
 
 
 
 
 
  
 
 SCAMP4
1372 Trametinib
 
 
 
 
  
 
 
 
 
 
  
 
 MPZL2
1372 Trametinib
 
 
 
 
  
 
 
 
 
 
  
 
 TTC9
1372 Trametinib
 
 
 
 
  
 
 
 
 
 
  
 
 INAVA
1372 Trametinib
 
 
 
 
  
 
 
 
 
 
  
 
 EPHA2
1372 Trametinib
 
 
 
 
  
 
 
 
 
 
 
 
 
 
 
 
  
  
 Figure 6 ¶  
 
 
 
  
 
 
 
 
 In [83]: 
 
 
     def   ploting_log2fc_deseq2_mek  (  dds_res  ,   genes  ,   title  ,   xlabel  ): 
     tmp2   =   dds_res  .  loc  [  genes  ,[  "log2FoldChange"  ,   "lfcSE"  ,   "padj"  ]]  .  reset_index  () 
     tmp2   =   tmp2  .  rename  (  columns  =  {  'gene_name'  :  'MEK1/2'  }) 
     fig  ,   ax   =   plt  .  subplots  (  figsize  =  (  6  ,   4  )) 
     sns  .  barplot  (  data  =  tmp2  ,   x  =  'MEK1/2'  ,   y  =  'log2FoldChange'  ,   ax  =  ax  ) 
     ax  .  set_title  (  title  ) 
     ax  .  set_xlabel  (  xlabel  ) 
 #     ax.set_ylim((-2,3)) 
     ax  .  bar_label  (  ax  .  containers  [  -  1  ],   labels  =  [  f  "padj:  {  p  :  .2e  }  "   for   p   in   tmp2  [  'padj'  ]],   label_type  =  'center'  ,   fontsize  =  6  ,   padding  =  6  ) 
     x_coords   =   [  p  .  get_x  ()   +   0.5   *   p  .  get_width  ()   for   p   in   ax  .  patches  ] 
     y_coords   =   [  p  .  get_height  ()   for   p   in   ax  .  patches  ] 
     ax  .  errorbar  (  x  =  x_coords  ,   y  =  y_coords  ,   yerr  =  tmp2  [  "lfcSE"  ],   fmt  =  "none"  ,   c  =  "k"  ) 

 brca_mek_genes   =   [  "MAP2K1"  ,   "MAP2K2"  ,   "GREB1"  ,   "TTC39A"  ,   "ANXA9"  ,   "MYB"  ,   "PGR"  ] 
 ov_mek_genes   =   [  "MAP2K1"  ,   "MAP2K2"  ,   "NT5C2"  ,   "CDC42BPB"  ,   "MYO5A"  ,   "PRKCH"  ,   "PCDH1"  ] 
 ucec_mek_genes   =   [  "MAP2K1"  ,   "MAP2K2"  ,   "TNFRSF21"  ,   "SCAMP4"  ,   "NT5C2"  ,   "MPZL2"  ,   "TTC9"  ] 
 cesc_mek_genes   =   [  "MAP2K1"  ,   "MAP2K2"  ,   "MALL"  ,   "NT5C2"  ,   "INAVA"  ,   "CDC42BPB"  ,   "EPHA2"  ] 
 all_mek_genes   =   [  "MAP2K1"  ,   "MAP2K2"  ,   "GREB1"  ,   "TTC39A"  ,   "ANXA9"  ,   "MYB"  ,   "PGR"  ,   "NT5C2"  ,   "CDC42BPB"  ,   "MYO5A"  ,   "PRKCH"  ,   "PCDH1"  ,   "TNFRSF21"  ,   "SCAMP4"  ,   "MPZL2"  ,   "TTC9"  ,   "MALL"  ,   "INAVA"  ,   "EPHA2"  ] 
 selected_mek_genes   =   [  'MALL'  ,   'TNFRSF21'  ,   'EPHA2'  ,   'PCDH1'  ,   'MPZL2'  ,   'INAVA'  ]     
 ploting_log2fc_deseq2_mek  (  brca_eeres_deseq2_res  ,   selected_mek_genes  ,   "BRCA"  ,   ""  ) 
 ploting_log2fc_deseq2_mek  (  ov_eeres_deseq2_res  ,   selected_mek_genes  ,   "OV"  ,   ""  ) 
 ploting_log2fc_deseq2_mek  (  ucec_eeres_deseq2_res  ,   selected_mek_genes  ,   "UCEC"  ,   ""  ) 
 ploting_log2fc_deseq2_mek  (  cesc_eeres_deseq2_res  ,   selected_mek_genes  ,   "CESC"  ,   ""  ) 
  
 
 
 
 
 
 
 
 
 
  
 
 
 
 
 
  
 
 
 
 
 
  
 
 
 
 
 
  
 
 
 
 
 
 
  
 
 
 
 
 In [97]: 
 
 
     def   ploting_log2fc_deseq2_mek  (  dds_res  ,   genes  ,   title  ,   xlabel  ): 
     tmp2   =   dds_res  .  loc  [  genes  ,[  "log2FoldChange"  ,   "lfcSE"  ,   "padj"  ]]  .  reset_index  () 
     tmp2   =   tmp2  .  rename  (  columns  =  {  'gene_name'  :  'MEK1/2'  }) 
     fig  ,   ax   =   plt  .  subplots  (  figsize  =  (  6  ,   4  )) 
     sns  .  barplot  (  data  =  tmp2  ,   x  =  'MEK1/2'  ,   y  =  'log2FoldChange'  ,   ax  =  ax  ) 
     ax  .  set_title  (  title  ) 
     ax  .  set_xlabel  (  xlabel  ) 
 #     ax.set_ylim((-2,3)) 
     ax  .  bar_label  (  ax  .  containers  [  -  1  ],   labels  =  [  f  "padj:  {  p  :  .2e  }  "   for   p   in   tmp2  [  'padj'  ]],   label_type  =  'center'  ,   fontsize  =  6  ,   padding  =  20  ) 
     x_coords   =   [  p  .  get_x  ()   +   0.5   *   p  .  get_width  ()   for   p   in   ax  .  patches  ] 
     y_coords   =   [  p  .  get_height  ()   for   p   in   ax  .  patches  ] 
     ax  .  errorbar  (  x  =  x_coords  ,   y  =  y_coords  ,   yerr  =  tmp2  [  "lfcSE"  ],   fmt  =  "none"  ,   c  =  "k"  ) 
    
 gene_related_to_er_and_mek   =   [  "EGFR"  ,   "RAF1"  ,   "MAPK1"  ,   "MAP2K1"  ] 
 ploting_log2fc_deseq2_mek  (  brca_eeres_deseq2_res  ,   gene_related_to_er_and_mek  ,   ""  ,   ""  ) 
 ploting_log2fc_deseq2_mek  (  ov_eeres_deseq2_res  ,   gene_related_to_er_and_mek  ,   ""  ,   ""  ) 
 ploting_log2fc_deseq2_mek  (  ucec_eeres_deseq2_res  ,   gene_related_to_er_and_mek  ,   ""  ,   ""  ) 
 ploting_log2fc_deseq2_mek  (  cesc_eeres_deseq2_res  ,   gene_related_to_er_and_mek  ,   ""  ,   ""  ) 
  
 
 
 
 
 
 
 
 
 
  
 
 
 
 
 
  
 
 
 
 
 
  
 
 
 
 
 
  
 
 
 
 
 
 
  
 
 
 
 
 In [52]: 
 
 
     def   finding_best_score_for_survival  (  es_dfs_df  ,   es_dss_df  ,   es_df  ): 
     bestes  =  None 
     bestp  =  1 
     sp   =   [] 

     for   i   in   np  .  arange  (  es_df  [  "EARLY"  ]  .  quantile  (  0.1  ),   es_df  [  "EARLY"  ]  .  quantile  (  0.9  ),   0.01  ): 
         results_dfs   =   km  .  fit  (  es_dfs_df  [  f  "DFS_MONTHS"  ],   es_dfs_df  [  f  "DFS_STATUS"  ],   (  es_dfs_df  [  "EARLY"  ]  >  i  )  .  apply  (  lambda   x  :   "High EERES"   if   x   else   "Low EERES"  )) 
         results_dss   =   km  .  fit  (  es_dss_df  [  f  "DSS_MONTHS"  ],   es_dss_df  [  f  "DSS_STATUS"  ],   (  es_dss_df  [  "EARLY"  ]  >  i  )  .  apply  (  lambda   x  :   "High EERES"   if   x   else   "Low EERES"  )) 
         p   =   (  results_dfs  [  'logrank_P'  ]  +  results_dss  [  'logrank_P'  ])  /  2 
         if   p  <  bestp  : 
             bestes   =   i 
             bestp   =   (  results_dfs  [  'logrank_P'  ]  +  results_dss  [  'logrank_P'  ])  /  2 
     print  (  "EERES Threshold"  ,   bestes  ) 
     print  (  "n"  ,   len  (  es_df  )) 
     print  (  "n >threshold"  ,   (  es_df  [  "EARLY"  ]  >  bestes  )  .  sum  ()) 
     print  (  "n <=threshold"  ,   (  es_df  [  "EARLY"  ]  <=  bestes  )  .  sum  ()) 
     results   =   km  .  fit  (  es_dfs_df  [  f  "DFS_MONTHS"  ],   es_dfs_df  [  f  "DFS_STATUS"  ],   (  es_dfs_df  [  "EARLY"  ]  >  bestes  )  .  apply  (  lambda   x  :   "High EERES"   if   x   else   "Low EESRS"  )) 
     km  .  plot  (  results  ,   full_ylim  =  True  ,   y_percentage  =  True  ,   fontsize  =  15  ) 
     plt  .  show  () 
     results   =   km  .  fit  (  es_dss_df  [  f  "DSS_MONTHS"  ],   es_dss_df  [  f  "DSS_STATUS"  ],   (  es_dss_df  [  "EARLY"  ]  >  bestes  )  .  apply  (  lambda   x  :   "High EERES"   if   x   else   "Low EESRS"  )) 
     km  .  plot  (  results  ,   full_ylim  =  True  ,   y_percentage  =  True  ,   fontsize  =  15  ) 
     plt  .  show  () 
     return   bestes 

 def   roc_curve_testing  (  X  ,   y  ,   title  ,   genes  ): 
     from   sklearn.preprocessing   import   StandardScaler 
     X_train  ,   X_test  ,   y_train  ,   y_test   =   train_test_split  (  X  ,   y  ,   test_size  =  0.2  ,   random_state  =  0  ) 
     print  (  len  (  y_test  )) 
     clf   =   LogisticRegression  (  random_state  =  0  )  .  fit  (  X_train  ,   y_train  ) 
     print  (  pd  .  DataFrame  ({  "Gene"  :  selected_mek_genes  ,   "Coef"  :  clf  .  coef_  [  0  ]})) 
     test_score   =   clf  .  predict_proba  (  X_test  ) 
     test_acc   =   clf  .  score  (  X_test  ,   y_test  ) 
     import   matplotlib.pyplot   as   plt 
     from   sklearn.metrics   import   RocCurveDisplay 
    

     RocCurveDisplay  .  from_predictions  ( 
         y_test  , 
         test_score  [:,  1  ], 
         name  =  "Low EERES vs High EERES"  , 
         color  =  "darkorange"  , 
         pos_label  =  1 
     ) 
     plt  .  plot  ([  0  ,   1  ],   [  0  ,   1  ],   "k--"  ,   label  =  "chance level (AUC = 0.5)"  ) 
     plt  .  axis  (  "square"  ) 
     plt  .  xlabel  (  "False Positive Rate"  ) 
     plt  .  ylabel  (  "True Positive Rate"  ) 
     plt  .  title  (  title  ) 
     plt  .  legend  () 
     plt  .  show  () 
    
 selected_mek_genes   =   [  'MALL'  ,   'TNFRSF21'  ,   'EPHA2'  ,   'PCDH1'  ,   'MPZL2'  ,   'INAVA'  ] 
 all_mek_genes   =   set  (  brca_mek_genes  +  ov_mek_genes  +  ucec_mek_genes  +  cesc_mek_genes  ) 

 brca_mek_y   =   pd  .  DataFrame  (  brca_pheno_bestes  ,   index  =  brca_es  .  iloc  [  brca_sortq  ]  .  index  ,   columns  =  [  "EERES"  ]) 
 brca_mek_X   =   brca_gdc_tpm  .  iloc  [:,  brca_sortq  ]  .  loc  [  selected_mek_genes  ]  .  transpose  () 
 roc_curve_testing  (  brca_mek_X  ,   brca_mek_y  .  applymap  (  lambda   x  :   0   if   x  ==  "Low EERES"   else   1  ),   "BRCA"  ,   selected_mek_genes  ) 

 ov_mek_y   =   pd  .  DataFrame  (  ov_pheno_bestes  ,   index  =  ov_es  .  iloc  [  ov_sortq  ]  .  index  ,   columns  =  [  "EERES"  ]) 
 ov_mek_X   =   ov_gdc_tpm  .  iloc  [:,  ov_sortq  ]  .  loc  [  selected_mek_genes  ]  .  transpose  () 
 roc_curve_testing  (  ov_mek_X  ,   ov_mek_y  .  applymap  (  lambda   x  :   0   if   x  ==  "Low EERES"   else   1  ),   "OV"  ,   selected_mek_genes  ) 

 ucec_mek_y   =   pd  .  DataFrame  (  ucec_pheno_bestes  ,   index  =  ucec_es  .  iloc  [  ucec_sortq  ]  .  index  ,   columns  =  [  "EERES"  ]) 
 ucec_mek_X   =   ucec_gdc_tpm  .  iloc  [:,  ucec_sortq  ]  .  loc  [  selected_mek_genes  ]  .  transpose  () 
 roc_curve_testing  (  ucec_mek_X  ,   ucec_mek_y  .  applymap  (  lambda   x  :   0   if   x  ==  "Low EERES"   else   1  ),   "UCEC"  ,   selected_mek_genes  ) 

 cesc_mek_y   =   pd  .  DataFrame  (  cesc_pheno_bestes  ,   index  =  cesc_es  .  iloc  [  cesc_sortq  ]  .  index  ,   columns  =  [  "EERES"  ]) 
 cesc_mek_X   =   cesc_gdc_tpm  .  iloc  [:,  cesc_sortq  ]  .  loc  [  selected_mek_genes  ]  .  transpose  () 
 roc_curve_testing  (  cesc_mek_X  ,   cesc_mek_y  .  applymap  (  lambda   x  :   0   if   x  ==  "Low EERES"   else   1  ),   "CESC"  ,   selected_mek_genes  ) 
  
 
 
 
 
 
 
 
 
 
  
 
 219
       Gene      Coef
0      MALL -0.092818
1  TNFRSF21 -0.008771
2     EPHA2 -0.006848
3     PCDH1  0.029096
4     MPZL2 -0.003317
5     INAVA -0.036396
 
 
 
 
  
 
 /home/oscar/.local/lib/python3.10/site-packages/sklearn/utils/validation.py:1141: DataConversionWarning: A column-vector y was passed when a 1d array was expected. Please change the shape of y to (n_samples, ), for example using ravel().
  y = column_or_1d(y, warn=True)
 
 
 
 
  
 
 
 
 
 
  
 
 76
       Gene      Coef
0      MALL  0.075162
1  TNFRSF21  0.009379
2     EPHA2 -0.000068
3     PCDH1  0.019487
4     MPZL2  0.027253
5     INAVA  0.010130
 
 
 
 
  
 
 /home/oscar/.local/lib/python3.10/site-packages/sklearn/utils/validation.py:1141: DataConversionWarning: A column-vector y was passed when a 1d array was expected. Please change the shape of y to (n_samples, ), for example using ravel().
  y = column_or_1d(y, warn=True)
 
 
 
 
  
 
 
 
 
 
  
 
 112
       Gene      Coef
0      MALL  0.068889
1  TNFRSF21  0.011314
2     EPHA2  0.000472
3     PCDH1  0.017722
4     MPZL2  0.014948
5     INAVA -0.011358
 
 
 
 
  
 
 /home/oscar/.local/lib/python3.10/site-packages/sklearn/utils/validation.py:1141: DataConversionWarning: A column-vector y was passed when a 1d array was expected. Please change the shape of y to (n_samples, ), for example using ravel().
  y = column_or_1d(y, warn=True)
 
 
 
 
  
 
 
 
 
 
  
 
 61
       Gene      Coef
0      MALL  0.151171
1  TNFRSF21  0.009883
2     EPHA2  0.003833
3     PCDH1  0.002883
4     MPZL2 -0.000882
5     INAVA  0.020528
 
 
 
 
  
 
 /home/oscar/.local/lib/python3.10/site-packages/sklearn/utils/validation.py:1141: DataConversionWarning: A column-vector y was passed when a 1d array was expected. Please change the shape of y to (n_samples, ), for example using ravel().
  y = column_or_1d(y, warn=True)
 
 
 
 
  
 
 
 
 
 
 
 
 
 
 
